# Synthesis of Glycosidic (β-1′′→6, 3′ and 4′) Site Isomers of Neomycin B and Their Effect on RNA and DNA Triplex Stability

**DOI:** 10.3390/molecules24030580

**Published:** 2019-02-06

**Authors:** Lotta Granqvist, Ville Tähtinen, Pasi Virta

**Affiliations:** Department of Chemistry, University of Turku, 20014 Turku, Finland; ltgran@hotmail.com (L.G.); vpotah@utu.fi (V.T.)

**Keywords:** aminoglycosides, DNA- and RNA-triple helices, groove binders

## Abstract

Glycosidic (β-1′′→6, 3′ and 4′) site isomers of neomycin B (i.e., neobiosamine (β-1′′→6, 3′ and 4′) neamines) have been synthesized in a straightforward manner. Peracetylated neomycin azide was used as a common starting material to obtain neobiosamine glycosyl donor and 6, 3′,4′-tri-*O*-acetyl neamine azide that after simple protecting group manipulation was converted to three different glycosyl acceptors (i.e., 5,6,4′-, 5,3′,4′- and 5,6,3′-tri-*O*-acetyl neamine azide). Glycosylation between the neobiosamine glycosyl donor and the neamine-derived acceptors gave the protected pseudo-tetrasaccharides, which were converted, via global deprotection (deacetylation and reduction of the azide groups), to the desired site isomers of neomycin. The effect of these aminoglycosides on the RNA and DNA triplex stability was studied by UV-melting profile analysis.

## 1. Introduction

**Scheme 1:**
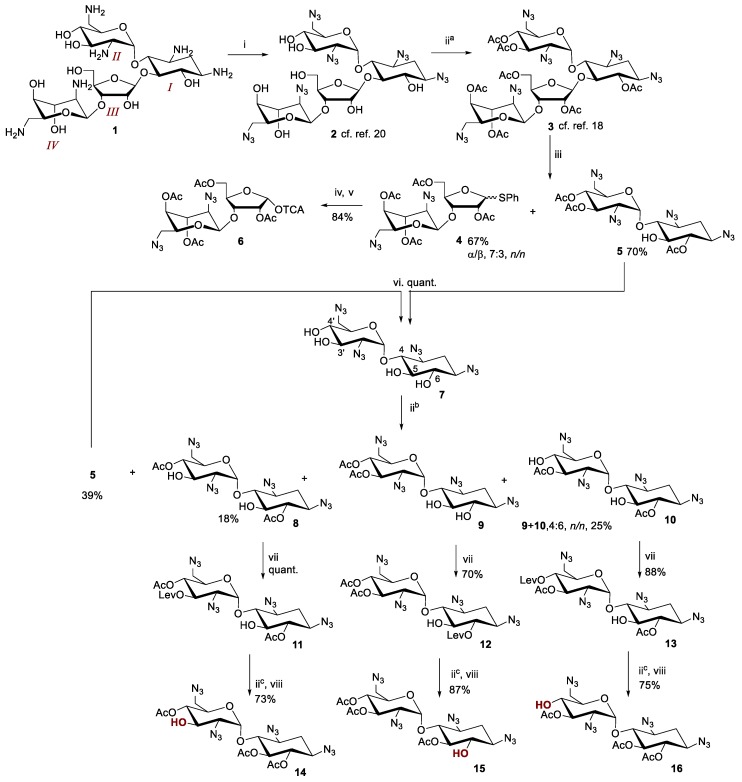
*Reagents and conditions:* i) CuSO_4_, TfN_3_, dichloromethane, H_2_O, ii^a-c^) Ac_2_O (a: 10 equiv., b: 2.5 equiv., c: 5 equiv.), DMAP, pyridine, overnight at r.t. iii) PhSH, BF_3_ · Et_2_O, DCM, iv) *N*-bromosuccinimide, trifluoroacetic acid, H_2_O, MeCN, dichloromethane, for 1h at 0 °C–r.t., v) CCl_3_CN, 1.8-diazabicyclo(5.4.0)undec-7-ene, DCM, for 1h at 0 °C, vi) 0.1 mol L^−1^ NaOMe/MeOH, for 1h at r.t., vii) Lev_2_O, 4-(*N,N*-dimethylamino)pyridine, pyridine, overnight at r.t., viii) NH_2_NH_2_·AcOH, MeOH, dichloromethane, for 3 h at r.t.

Among the small molecular ligands that target nucleic acids, aminoglycosides (AGs) deserve special attention [1]. Their binding to a variety of nucleic acid targets has been extensively studied. Binding to the ribosomal decoding site is the basis of AGs’ bactericidal effect [2,3,4,5]. The continuous increase of antibiotic resistant infectious diseases maintains the interest around this RNA target, and a significant effort has been paid to optimization and modification of existing AG-based lead compounds to provide new potential antibacterial drugs [6,7]. Relatively high binding affinities have also been reported for other structurally resembling binding sites in ribozymes [7,8] and important regions of HIV RNAs (Trans Activation Response element (TAR), Reverse Response Element (RRE) and Dimerization Initiation Site (DIS)) [9,10,11,12]). In these RNA targets, the binding sites are bulges and internal loops, which, in contrast to canonical double helices, are able to form appropriate hydrogen bonds and electrostatic interactions with AGs. AGs can also act as groove binders that stabilize DNA- and RNA-triple helices and their hybrids [13,14,15,16,17]. Among the AGs studied for the triplex recognition, neomycin B has shown to be the most effective groove binder. The binding occurs to the Watson–Hoogsteen groove, in which favorable contacts with rings II and IV (cf. Scheme 1), together with the appropriate shape complementarity, may take place [14,17]. In each case, however, electrostatic interactions dominate AGs’ affinity to nucleic acids, which makes the binding promiscuous and may disturb detailed characterization of the binding motifs. Conformational adaption of the nucleic acid targets (in particular RNA) disturbs modelling and discovery of specific ligands even further [1]. Chemically synthesized structural analogs of the known AG-ligands are hence important tools that may be used for the exclusion analysis of the binding requirements.

**Scheme 2:**
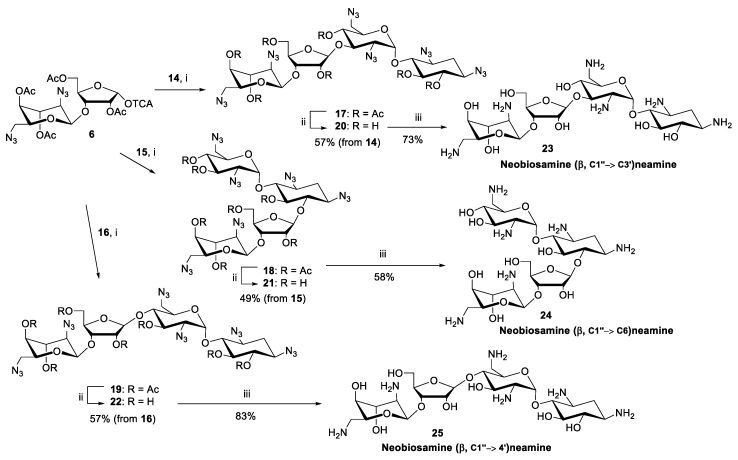
*Reagents and conditions:* i) TMSOTf, DCM, 2 h at −20 °C; ii) 0.1 mol L^−1^ NaOMe/MeOH, 1 h at r.t.; iii) 1: Me_3_P, toluene, water, dioxane, 4 h at r.t. under N_2_, 2: conc. ammonia, overnight at r.t.

In the present study, a straightforward synthesis of glycosidic (β-1″→6, 3′ and 4′) site isomers of neomycin B is described (Note: C6–, C3′– and C4′–substituted neamines are not neomycin class AGs, i.e., 4,5-disubstituted deoxystreptamines, and the correct names for these compounds are neobiosamine(β-C1″→C6, 3′ and 4′)neamines). The key steps of the synthesis (Scheme 1) were (1) acid-catalyzed thiolysis of neomycin azide [18], which gave useful intermediates for both glycosyl donor and the acceptors, and (2) selective diacetylation of neamine azide, which after simple protecting group manipulation afforded the glycosyl acceptors. Glycosylation between the neobiosamine donor and triacetylated neamine acceptors resulted in the azide masked pseudo-tetrasaccharides, which, via global deprotection, were converted to the desired site isomers of neomycin B (Scheme 2). In order to evaluate how the changed glycosidic connection (β-1″→3′,4′ or 6) between the neamine and neobiosamine cores affects the groove binding, the stability of RNA and DNA triple helix models in the presence of these structural analogs (and neomycin B) was studied by UV-melting profile analysis.

## 2. Results and Discussion

### 2.1. Synthesis of Glycosyl Donor 6 and Acceptors ***14**–**16***

Acid-catalyzed degradation of neomycin B azide (**3**) has been studied in detail by Wu et al. [18]. Various conditions by using different Lewis acids (SnCl_4_, TMSOTf and BF_3_·OEt_2_) and thiols (propylenedithiol, EtSH and TolSH) have been evaluated for the selective cleavage. According to this data, **3** was cleaved with thiophenol in the presence of BF_3_·OEt_2_, to yield an anomeric mixture of phenylthio 2,5,3′4′-tetra-*O*-acetyl 2′,6′-diazidoneobiosamine (**4**) and 6,3′,4′-tri-*O*-acetyl 1,3,2′,6′-tetraazido neamine (**5**) in high yields (67% and 70%, respectively, iii in Scheme 1).

The anomeric mixture of phenylthio 2,5,3′4′-tetra-*O*-acetyl 2′,6′-diazidoneobiosamine (**4**) may be used as such as a glycosyl donor, but the mixture of the donor makes the control and monitoring of the reaction (cf. Scheme 2) complex. Therefore, the phenylthioglycoside (**4**) was hydrolyzed and the resulted hemiacetal converted to pure α-anomer of tricloroacetimidate **6**. Overall, this leaving group conversion (from **4** to **6**) could be carried out in 86% yield (iv and v/Scheme 1).

The 5-OH group of neamine (**7**) is less nucleophilic than the 6-, 3′- and 4′-OH groups. For this reason, selective triacetylation of *N*-protected neamines (e.g., **5** from **7**) may be readily carried out as described previously in several studies [19,20]. On using a smaller excess of acetic anhydride, diacetylated neamines (**8**–**10**) may also be obtained, albeit the yield and ratio of the products (**8**, **9**, **10**, **5** and monoacetylated neamines) are more sensitive to reaction conditions (temperature, reaction time, and the addition rate and excess of acetic anhydride). Overnight reaction in pyridine in the presence of a catalytic amount of 4-(*N,N*-dimethylamino)pyridine (DMAP) and 2.5 equiv. of acetic anhydride (slowly added to the reaction mixture) resulted in the desired diacetylated neamines **8**, **9** and **10** in 18%, 10% and 15% yield, respectively (**9** and **10** isolated as a mixture (4:6, *n*/*n*)) (ii^b^/Scheme 1). The triacetylated neamine **5** was obtained as the major product (39% yield), but it could be readily converted back to the starting material (**7**) and the reaction repeated. It may be worth mentioning that a smaller excess of acetic anhydride did not markedly improve the yields of **8**–**10**. Moreover, the increased ratio of monoacetylated neamines disturbed the chromatographic isolation of the diacetylated products. Thus, the slight ‘overacetylation’ was beneficial. The modest reactivity of the 5-OH group of the diacetylated neamines (**8**, **9** and **10**) was next utilized for the selective levulinoylation of the 3′-, 6- and 4′-OH groups to obtain **11**–**13** (70% – quant., vii/Scheme 1). The reaction was carried out in pyridine using 1.5 equiv. of levulinic anhydride in the presence of a catalytic amount of DMAP. The remained free 5-OH group of **11**–**13** was finally acetylated using an excess of acetic anhydride (5 equiv. ii^c^/Scheme 1) and subsequent removal of the levulinoyl group with hydrazine hydrate (viii/Scheme 1) gave the desired glycosyl acceptors **14**–**16** in 73–87% yield.

### 2.2. Glycosylation and Global Deprotection

**Figure 1:**
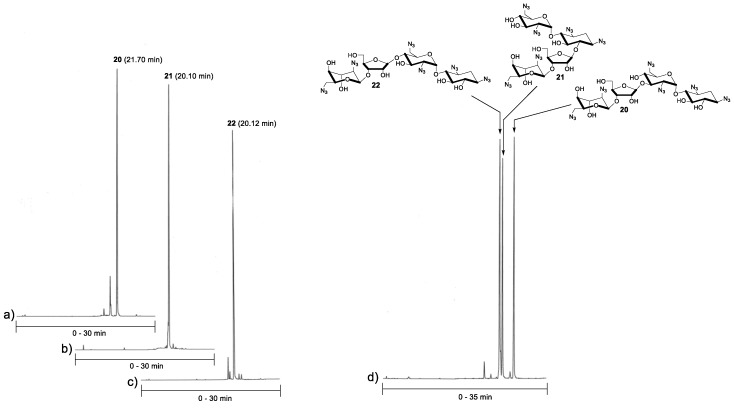
RP HPLC profiles of the azide-masked neobiosamine neamines (**20**–**22**). Conditions: An analytical RP HPLC (C18, 250 × 4.6 mm, 5 µm) column, detection at λ = 220 nm, gradient elutions from 10% to 80% (a–c) and from 10% to 60% (d) acetonitrile in H_2_O over 30 min (a–c) and 35 min (d).

A standard procedure using TMSOTf as a catalyst was applied for the glycosylation between acceptors **14**–**16** and donor **6** (1.5 equiv.). Each reaction was performed at −20 °C (for 2 h) in dry dichloromethane under nitrogen. For the purification reasons (the fully protected pseudo-tetrasaccharides **17**–**19** remained contaminated by traces of donor **6**, despite a laborious chromatographic purification), the glycosylation was followed by subsequent deacetylation (0.1 mol L^−1^ NaOMe in MeOH) and the neomycin azides **20**–**22** could be isolated in acceptable yields (49–69%). The purity of the products (**20**–**22**) was confirmed by reversed phase-high performance liquid chromatography (RP HPLC) analysis (Figure 1). Finally, the azide masks were removed by Staudinger reaction using trimethylphosphine and aqueous ammonia, followed by elution through an ion exchange resin, to give the glycosidic (β-C1″→C6, C3′ and C4′) site isomers of neomycin B (**23**–**25**) in 58–83% yields.

### 2.3. The Effect of ***23***–***25*** on DNA and RNA Triplex Stability

**Figure 2:**
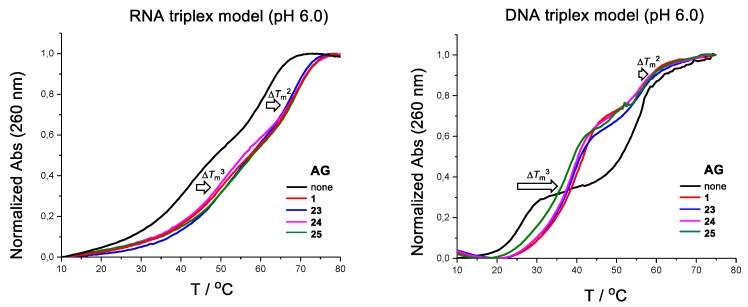
Representative melting profiles of the DNA and RNA triplex models in the presence of aminoglycosides (AGs) (2 μmol oligonucleotides in the presence of 5 eq. 1, 23–25, 10 mmol L^−1^ sodium cacodylate, 0.1 mol L^–1^ NaCl, pH 6.0.

The effect of the glycosidic site isomers of neomycin B (**23**–**25**) and neomycin (**1**) on the stability of simple DNA and RNA triple helices was studied by UV-melting profile experiments (Table 1). The DNA triple helix is consisted of a purine rich region of c-Myc promoter 1 [21], used as a model in several previous studies [22,23,24,25,26,27]. The formation of this triple helix, consisting of CH^+^•G-C-triplets, requires slightly acidic pH. The intramolecular RNA triplex is also a known model (in fact, this model may exist as a mixture with its dimer [28]). The measurements were carried out using 2 µmol L^−1^ of the oligonucleotides in a mixture of 10 mmol L^−1^ sodium cacodylate and 0.1 mol L^−1^ NaCl at pH 6.0 (both RNA and DNA model) and at pH 7.0 (RNA model only) in the presence of 5 and 10 eq. of the AGs (**1** and **23**–**25**). The temperature was changed at a rate of 0.2 °C min^−1^. The *T*_m_^3^- and *T*_m_^2^-values (triplex and duplex melting points) were extracted from the first and second inflection points of the biphasic melting curves at λ = 260 nm, respectively (Figure 2). Consistent with the previous findings (affinity of neomycin follows the trend: DNA duplex < DNA triplex < RNA duplex ~ RNA triplex [17]), AGs stabilized both the triplex and the duplex on the RNA model at pH 6.0, whereas basically only the triplex on the DNA model (Δ*T*_m_^3^ vs. Δ*T*_m_^2^ = +11.1−18.6 °C vs. +0.7−2.3 °C). A slight selectivity of the stabilization on the RNA triplex vs. duplex may be observed at pH 7.0 (Δ*T*_m_^3^ vs. Δ*T*_m_^2^ = +6.9−7.8 °C vs. +1.1−1.9 °C). Most importantly, AGs’ effect on the triplex (both on RNA and DNA model) did not show a marked difference when AGs are compared to each other. Only a slight variation was observed. For example, on the DNA model, the stability of the triplex increased by Δ*T*_m_ = +18.6 °C, +17.4 °C, +15.8 °C and +13.9 °C in the presence of 10 equiv. of neomycin B (**1**), the C1″→C4′ (**25**), C1″ →C3′ (**23**) and C1″→C6 (**24**) site isomers, respectively. On the RNA triplex, the effect with the C1″→C3′ (**23**) and C1″→C6 (**24**) site isomers was, in fact, slightly higher compared to that with neomycin B (**1**) (Δ*T*_m_^3^ = +11.3 and +10.6 °C vs. +9.3 °C). Probably, the carbohydrate core and spatial orientation of the amino groups on it do not play a marked enough role to result in discrimination between the affinities of the glycosidic site isomers (**1**, **23**–**25**). This may be an unexpected observation as, e.g., previous studies with kanamycin class aminoglycosides (kanamycin = a 4,6-disubstituted deoxystreptamine consisted of neamine and α-d-glucos-3-amine at C6) show a modest or no effect on the poly U-A-U DNA triplex [14]. The effect of **24** (a 4,6-disubstituted deoxystreptamine) on the DNA triplex was the smallest (Δ*T*_m_^3^ = +13.9 °C) of the isomers (**23**–**25**), but still comparable to neomycin B (**1**, Δ*T*_m_^3^ = +18.6 °C), the most effective aminoglycosidic DNA triplex groove binder to date. The biosamine moiety, or the l-neosamine (Ring IV in Scheme 1), hence plays a marked role in the groove binding despite the glycosidic site on the neamine core.

## 3. Materials and Methods

### General Remarks 

Pyridine, methanol and dichloromethane were dried over 3Å molecular sieves. Nuclear magnetic resonance (NMR) spectra were recorded using a 500 MHz instrument. The chemical shifts for ^1^H and ^13^C-NMR resonances are given in parts of million from the residual signal of the deuterated solvents (CD_3_OD and CD_3_Cl_3_). Mass spectra were recorded using electrospray ionization (ESI-TOF). The NMR spectral data for all new compounds are showed in the Supplementary Materials.

*2,5,3′,4′-Tetra-O-acetyl-2′,6′-diazido-1-phenylthio-α/β-neobiosamine* (**4**). Peracetylated neomycin azide (**3**, 5.8 g, 5.6 mmol, synthesized according to the literature [18,20]) and PhSH (0.64 mL, 6.2 mmol) were dissolved in dry dichloromethane (50 mL). The mixture was cooled down to 0 °C, and BF_3_·Et_2_O (2.1 mL, 17 mmol) was slowly added under nitrogen. The mixture was stirred first at 0 °C for 20 min, and allowed then to warm up to room temperature. After overnight reaction, triethylamine (1.5 mL) was added, the mixture was diluted with dichloromethane (150 mL), and washed with water (20 mL). The organic phase was dried over Na_2_SO_4_, filtered and evaporated to dryness. The residue was purified by silica gel chromatography (30-40% EtOAc in petroleum ether) to give an anomeric mixture of **4** (2.3 g, 67%, α/β = 7:3, *v*/*v*, pure fractions used for the NMR characterization) and 1,3,2′,6′-tetra azido-6,3′,4′-tri-*O*-acetyl neamine (**5**, 2.2 g, 70%). **4**: *β-anomer:* ^1^H (500 MHz, CDCl_3_): *δ* 7.52–7.50 (m, 2H), 7.34–7.28 (m, 3H), 5.74 (d, 1H, *J* = 4.7 Hz), 5.48 (t, 1H, *J* = 4.2 Hz), 5.06 (t, 1H, *J* = 2.7 Hz), 5.03 (d, 1H, *J* = 1.5 Hz), 4.73 (b, 1H), 4.50–4.47 (m, 3H), 4.26 (m, 1H), 4.14 (m, 1H), 3.64 (dd, 1H, *J* = 13.1 and 8.6 Hz), 3.42 (b, 1H), 3.22 (dd, 1H, *J* = 13.1 and 3.7 Hz), 2.20, 2.20, 2.18 and 2.09 (4 × s, 3H each); ^13^C (125 MHz, CDCl_3_): *δ* 170.7, 170.2, 169.9, 168.5, 134.5, 131.5, 129.1, 127.5, 98.5, 89.1, 78.4, 75.8, 73.9, 72.6, 68.8, 65.8, 63.0, 56.4, 50.7, 20.8, 20.8, 20.7, 20.6; HRMS (ESI-TOF) calculated for C_25_H_30_N_6_KO_11_S [M + K]^+^: 661.1330, observed: 661.1332. *α-anomer:* ^1^H (500 MHz, CDCl_3_): *δ* 7.54–7.52 (m, 2H), 7.38–7.33 (m, 3H), 5.43 (d, 1H, *J* = 2.9 Hz), 5.26 (dd, 1H, *J* = 5.1 and 3.0 Hz), 5.05 (dd, 1H, *J* = 2.9 and 2.8 Hz), 4.92 (d, 1H, *J* = 1.8 Hz), 4.73 (t, 1H, *J* = 2.0 Hz), 4.51 (dd, 1H, *J* = 12.1 and 2.6 Hz), 4.45 (dd, 1H, *J* = 7.3 and 5.2 Hz), 4.31 (m, 1H), 4.23 (dd, 1H, *J* = 12.1 and 5.1 Hz), 4.11 (ddd, 1H, *J* = 8.1, 4.4 and 1.8 Hz), 3.61 (dd, 1H, *J* = 13.0 and 8.2 Hz), 3.34 (dd, 1H, *J* = 2.1 and 2.0 Hz), 3.29 (dd, 1H, *J* = 13.0 and 4.4 Hz), 2.19, 2.18, 2.13 and 2.10 (4 × s, each 3H); ^13^C (125 MHz, CDCl_3_): *δ* 170.7, 170.3, 169.8, 168.6, 132.9, 132.2, 129.1, 128.3, 98.9, 88.7, 80.1, 76.5, 75.0, 73.5, 68.7, 65.7, 63.2, 56.4, 50.6, 20.9, 20.8, 20.7, 20.7; HRMS (ESI-TOF) calculated for C_25_H_30_N_6_NaO_11_S [M + Na]^+^: 645.1591, observed: 645.1563.

*2,5,3′,4′-Tetra-O-acetyl-2′,6′-diazido-neobiosamine trichloroacetimidate* (**6**). *N*-bromosuccinimide (NBS) (0.77 g, 4.3 mmol) and trifluoroacetic acid (0.30 mL) were added to a two-phase mixture of **4** (1.4 g, 2.2 mmol, as an anomeric mixture) in dichloromethane (15 mL), acetonitrile (7.5 mL) and H_2_O (15 mL) at 0 °C. The mixture was allowed to warm up to ambient temperature, stirred for 1 h, poured to saturated NaHCO_3_ and extracted with dichloromethane. The organic layers were combined, dried over Na_2_SO_4_, filtered and evaporated to dryness. The residue was purified by silica column chromatography (5% MeOH in DCM) to give an anomeric mixture of the neobiosamine hemiacetal (1.1 g, 98%) as a white foam. ^1^H-NMR (500 MHz, CDCl_3_) δ: 5.52 (dd, 0.25H, *J* = 10.3 and 4.1 Hz), 5.41 (d, 1H, *J* = 2.5 Hz), 5.23 (d, 1H, *J* = 4.5 Hz), 5.08 (dd, 0.25 H, *J* = 3.2 and 3.1 Hz), 5.05–5.02 (m, 1.25H), 5,00 (d, 0.25H, *J* = 2.7 Hz), 4.99 (d, 1H, *J* = 1.8 Hz), 4.75 (t, 0.25H, *J* = 2,3 Hz), 4.72 (dd, 1H, *J* = 1.9 and 1.8 Hz), 4.67 (dd, 1H, *J* = 7.5 and 4.5 Hz), 4.53 (m, 0.25H), 4.50 (dd, 1H, *J* = 11.6 and 2.3 Hz), 4.46 (t, 0.25H, *J* = 5.2 Hz), 4.38 (dd, 0.25H, *J* = 12.2 and 2.8 Hz), 4.31 (m, 1H), 4.28–4.21 (m, 1.25H), 4.15 (ddd, 1H, *J* = 8.3, 4.1 and 1.8 Hz), 4.11 (ddd, 0.25H, *J* = 8.5, 4.0 and 2.0 Hz), 3.66–3.60 (m, 1.25H), 3.46 (t, 0.25H, *J* = 2.4 Hz), 3.42 (d, 0.25H, *J* = 4.1 Hz), 3.39 (d, 1H, *J* = 3.0 Hz), 3.36 (t, 1H, *J* = 1.9 Hz), 3.29 (dd, 0.25H, *J* = 13.1 and 4.1 Hz), 3.24 (dd, 1H, *J* = 13.1 and 4.1 Hz), 2.20, 2.18, 2.17, 2.13, 2.11 and 2.11 (each s, 15H); ^13^C-NMR (125 MHz, CDCl_3_) δ: 171.0, 170.3, 169.9, 168.6, 100.2, 99.1, 98.6, 95.8, 79.3, 79.1, 76.4, 76.2, 74.4, 73.8, 73.5, 71.9, 68.8, 68.7, 65.8, 65.8, 64.5, 63.4, 50.8, 20.9, 20.8, 20.7, 20.7; MS (ESI-tOF): calculated for C_19_H_26_N_6_NaO_12_ [M + Na]^+^: 553.1506, observed 553.1488. The hemiacetal (1.0 g, 1.9 mmol) was dissolved in a mixture of dichloromethane (1.0 mL) and trichloroacetonitrile (1.9 mL, 19 mmol). The mixture was cooled down to 0 °C and 1.8-diazabicyclo(5.4.0)undec-7-ene (DBU) (56 μL, 0.38 mmol) was added. The mixture was stirred for 1 h at 0 °C and eluted as such (without a work up) through a silica gel column (1% Et_3_N, 50% EtOAc in petroleum ether) to give 1.2 g (91%, 84% overall yield from **4**) of the trichloroacetimidate product (**6**) as a white foam. ^1^H-NMR (500 MHz, CDCl_3_): *δ* 8.36 (s, 1H), 6.36 (s, 1H), 5.47 (d, 1H, *J* = 4.6 Hz), 5.06–5.04 (m, 2H) 4.73 (dd, 1H, *J* = 1.9 and 1.8 Hz), 4.68 (dd, 1H, *J* = 8.1 and 4.5 Hz), 4.53 (dd, 1H, *J* = 12.1 and 2.8 Hz), 4.45 (m, 1H), 4.22 (dd, 1H, *J* = 12.2 and 6.4 Hz), 4.19 (ddd, 1H, *J* = 8.6, 3.7 and 1,8 Hz), 3.63 (dd, 1H, *J* = 13.1 and 8,6 Hz), 3.35 (dd, 1H, *J* = 2.1 and 1.9 Hz), 3.21 (dd, 1H, *J* = 13.1 and 3.7 Hz), 2.20, 2.19, 2.18, 2.11 (each s, each 3H); ^13^C-NMR (125 MHz, CDCl_3_): *δ* 170.8, 170.1, 169.8, 168.6, 160.3, 102.7, 98.4, 90.6, 80.2, 75.3, 73.9, 73.0, 68.7, 65.8, 64.0, 56.3, 50.7, 20.8, 20.8, 20.7, 20.6; MS (ESI-TOF): calculated for C_21_H_26_Cl_3_N_7_NaO_12_ [M + Na]^+^: 696.0603, observed 696.0631.

*Synthesis of diacetylated neamine azides* (**8**, **9** and **10**). Acetic anhydride (2.3 mL, 24 mmol) was slowly added to a mixture of neamine azide (**7**, 4.0 g, 9.3 mmol) in pyridine (20 mL). The mixture was stirred overnight at ambient temperature, concentrated, poured to saturated NaHCO_3_ and extracted with ethyl acetate. The organic layers were combined, washed with brine, dried over Na_2_SO_3_, filtered and evaporated to dryness. The residue was purified by silica gel chromatography (40% EtOAc in petroleum ether) to give three fractions of products. The first eluting compound was 6,3′,4′-tri-*O*-acetyl-1,3,2′,6′-tetra-azido-neamine (**5**, 2.0 g, 39%, the main product). The second fraction was a mixture of 3′,4′-di-*O*-acetyl- (**9**) and 6, 3′-di-*O*-acetyl- (**10**) 1,3,2′,6′-tetra-azidoneamines (**9**:**10**, 4:6, *n/n*, 1.3 g, 25%) and the third fraction was pure 6,4′-di-*O*-acetyl-1,3,2′,6′-tetra-azidoneamine (**8**, 0.87 g, 18%). Each product was obtained as a white foam. Compound **8**: ^1^H (500 MHz, CDCl_3_): *δ* 5.32 (d, 1H, *J* = 3.4 Hz), 4.96 (t, 1H, *J* = 10.0 Hz), 4.91 (t, 1H, *J* = 9.7 Hz), 4.24 (ddd, 1H, *J* = 10.0, 5.0 and 2.8Hz), 4.12 (dd, 1H, *J* = 9.0 and 8.6 Hz), 3.86 (d, 1H, *J* = 3.3 Hz), 3.67 (ddd, 1H, *J* = 12.2, 9.5 and 2.9 Hz), 3.60 (dd, 1H, *J* = 10.1 and 3.7 Hz), 3.54 (ddd, 1H, *J* = 12.5, 10.0 and 4.6 Hz), 3.46–3.41 (m, 2H), 3.38–3.33 (m, 2H), 3.03 (b, 1H), 2.40 (ddd, 1H, *J* = 13.4, 4.6 and 4.5 Hz), 2.20 (s, 3H), 2.18 (s, 3H), 1.63 (ddd, 1H, *J* = 12.6 Hz, each); ^13^C (125 MHz, CDCl_3_): *δ* 171.4, 170.5, 98.9, 83.7, 74.9, 74.4, 72.3, 71.7, 69.5, 64.2, 58.6, 58.0, 51.0, 32.0, 20.9. A mixture of **9** + **10**: ^1^H (500 MHz, CDCl_3_): *δ* 5.51 (dd, 0.4H, *J* = 10.3 and 9.4 Hz), 5.36 (d, 0.4H, *J* = 5.0 Hz), 5.34 (d, 0.6H, *J* = 3.7 Hz), 5.30 (dd, 0.6H, *J* = 10.1 and 9.7 Hz), 5.07 (dd, 0.4H, *J* = 9.9 and 9.6 Hz), 4.94 (dd, 0.6H, *J* = 9.8 and 9.7 Hz), 4.37 (ddd, 0.4H, *J* = 10.2, 4.9 and 2.7 Hz), 4.17 (ddd, 0.6H, *J* = 10.1, 4.3 and 2.8 Hz), 3.77 (d, 0.4H, *J* = 2.7 Hz), 3.72–3.62 (m, 3.4H), 3.59–3.52 (m, 1.6H), 3.47–3.31 (m, 3.2H), 2.98 (b, 0.4H), 2.78 (d, 0.6H, *J* = 5.6 Hz), 2.43–2.36 (m, 1H), 2.22 (s, 1.8H), 2.20 (s, 1.8 H), 2.12 (s, 1.2 H), 2.08 (s, 1.2 H), 1.66–1.55 (m, 1H); ^13^C (125 MHz, CDCl_3_): *δ* 172.1, 170.5, 170.2, 169.8, 99.0, 98.6, 83.7, 83.2, 75.9, 75.4, 74.9, 74.8, 74.4, 71.9, 71.3, 69.9, 69.5, 69.3, 61.72, 61.71, 59.7, 58.6, 58.4, 57.9, 53.5, 50.9, 50.8, 32.0, 31.9, 20.9, 20.8, 20.7, 20.6.

*6,4′-Di-O-acetyl-3′-O-levulinyl-1,3,2′,6′-tetra-azidoneamine* (**11**). Freshly prepared levulinic anhydride (63 mg, 0.29 mmol) was added to a mixture of **8** (0.10 g, 0.20 mmol) in pyridine (2.0 mL) and a catalytic amount of 4-(*N,N*-dimethylamino)pyridine was added. The mixture was stirred overnight at ambient temperature, poured to saturate NaHCO_3_, and the product was extracted with dichloromethane. The organic layer was separated, dried over Na_2_SO_4_ and evaporated to dryness. The residue was purified by silica gel chromatography (5% MeOH in DCM) to give 120 mg (quant.) of the product (**11**) as a white foam. ^1^H-NMR (500 MHz, CDCl_3_): *δ* 5.52 (dd, 1H, *J* = 10.1 and 9.9 Hz), 5.43 (d, 1H, *J* = 3.6 Hz), 5.07 (t, 1H, *J* = 9.8 Hz), 4.94 (dd, 1H, *J* = 10.0 & 9.9 Hz), 4.34 (ddd, 1H, *J* = 7.9, 5.0 and 2.7 Hz), 3.88 (b, 1H), 3.69 (t, 1H, *J* = 9.5 Hz), 3.61 (dd, 1H, *J* = 10.6 and 3.6 Hz), 3.54 (ddd, 1H, *J* = 12.4, 10.2 and 4.4 Hz), 3.47 (dd, 1H, *J* = 9.8 and 9.2 Hz), 3.43–3.35 (m, 2H), 3.32 (dd, 1H, *J* = 13.5 and 5.2 Hz), 2.82 (m, 1H), 2.71 (m, 1H), 2.62 (m, 1H), 2.54 (m, 1H), 2.41 (ddd, 1H, *J* = 13.4, 4.5 and 4.5 Hz), 2.19 (s, 3H), 2.17 (s, 3H), 2.13 (s, 3H), 1.63 (ddd, 1H, *J* = 12.6 Hz. each); ^13^C (125 MHz, CDCl_3_): *δ* 206.0, 171.9, 170.5, 170.0, 98.8, 83.2, 75.1, 74.4, 71.0, 69.6, 68.9, 61.8, 58.3, 57.9, 50.9, 37.6, 31.9, 29.7, 27.9, 20.8, 20.7; HRMS (ESI-TOF) calculated for C_21_H_28_N_12_NaO_10_ [M + Na]: 631.1949, observed: 631.1924.

*3′,4′-Di-O-acetyl-6-O-levulinyl-1,3,2′,6′-tetra-azidoneamine* (**12**) *and 6,3′-Di-O-acetyl-4′-levulinyl-1,3,2′,6′-tetra-azidoneamine* (**13**). The mixture of **9** and **10** (1:2, *n/n*, 0.38 g, 0.74 mmol) was treated as described for **11** (from **8**) above. 0.11 g (70%) of **12** and 0.27g (88%) of **13** were obtained as white foams. **12**: ^1^H-NMR (500 MHz, CDCl_3_): *δ* 5.57 (d, 1H, *J* = 3.7 Hz), 5.48 (dd, 1H, *J* = 11.1 and 9.3 Hz), 5.04 (dd, 1H, *J* = 10.1 and 9.4 Hz), 4.94 (t, 1H, *J* = 9.9 Hz), 4.39 (ddd, 1H, *J* = 10.1, 5.0 and 2.8 Hz), 3.87 (d, 1H, *J* = 3.1 Hz), 3.76 (ddd, 1H, *J* = 9.4, 9.4 and 3.0 Hz), 3.53–3.42 (m, 2H), (t, 1H, *J* = 7.0 Hz), 3.50 (dd, 1H, *J* = 10.7 and 3.7 Hz), 3.39 (dd, 1H, *J* = 13.5 and 2.9 Hz), 3.31 (dd, 1H, *J* = 13.4 and 5.1 Hz), 2.93 (m, 1H), 2.81 (m, 1H), 2.66 (m, 1H), 2.57 (m, 1H), 2.40 (ddd, 1H, *J* = 13.3, 4.5 and 4.5 Hz), 2.21 (s, 3H), 2.10 (s, 3H), 2.06 (s, 3H), 1.64 (ddd, 1H, *J* = 12.5 Hz, each); ^13^C (125 MHz, CDCl_3_): *δ* 207.8, 172.3, 170.2, 169.7, 98.4, 81.4, 75.8, 74.7, 70.6, 69.4, 69.3, 61.2, 58.5, 57.7, 50.8, 38.4, 31.9, 29.8, 28.1, 20.7, 20.6; HRMS (ESI-TOF) calculated for C_21_H_28_N_12_NaO_10_ [M + Na]: 631.1949, observed: 631.1970. **13**: ^1^H-NMR (500 MHz, CDCl_3_): *δ* 5.52 (dd, 1H, *J* = 10.3 and 9.6 Hz), 5.38 (d, 1H, *J* = 3.6 Hz), 5.07 (t, 1H, *J* = 9.8 Hz), 4.94 (t, 1H, *J* = 9.8 Hz), 4.32 (ddd, 1H, *J* = 10.2, 4.9 and 2.8 Hz), 3.69 (m, 1H), 3.64 (dd, 1H, *J* = 10.6 and 3.7 Hz), 3.55 (ddd, 1H, *J* = 12.6, 10.1 and 4.6 Hz), 3.48–3.36 (m, 3H), 2.76 (m, 2H), 2.51 (m, 2H), 2.41 (ddd, 1H, *J* = 13.5, 4.6 and 4.5 Hz), 2.18 (s, 6H), 2.14 (s, 3H), 1.63 (ddd, 1H, *J* = 12.5 Hz, each); ^13^C (125 MHz, CDCl_3_): *δ* 206.2, 171.6, 170.3, 98.8, 83.5, 75.0, 74.4, 70.7, 69.6, 69.2, 61.8, 58.3, 57.9, 50.7, 37.7, 31.9, 29.6, 27.8, 20.7, 20.7; HRMS (ESI-TOF) calculated for C_21_H_28_N_12_NaO_10_ [M + Na]: 631.1949, observed: 631.1961.

*5,6,4′-Tri-O-acetyl-1,3,2′,6′-tetra-azidoneamine* (**14**). Acetic anhydride (85 μL, 0.90 mmol) was added to a mixture of **11** (0.11 g, 0.18 mmol) and a catalytic amount of 4-(*N,N*-dimethylamino)pyridine in pyridine (2.0 mL). The mixture was stirred at ambient temperature for 3 h, poured to saturated NaHCO_3_, and the product was extracted with dichloromethane. The organic layers were combined, dried over Na_2_SO_4_, and evaporated to dryness. The residue was dissolved in a mixture dichloromethane (6.0 mL) and methanol (1.0 mL) and hydrazine acetate (34 mg, 0.36 mmol) was added. The mixture was stirred at ambient temperature for 3 h, poured to 10% aqueous KH_2_PO4, and the product was extracted with dichloromethane. The organic layers were combined, washed with brine, dried over Na_2_SO_4_, and evaporated to dryness. The residue was purified by silica gel chromatography (2% MeOH in DCM) to give 73 mg (73%) of the product **14** as a white foam. ^1^H-NMR (500 MHz, CDCl_3_): *δ* 5.15 (dd, 1H,m *J* = 9.8 Hz, both), 5.14 (d, 1H, *J* = 3.3 Hz), 4.95 (t, 1H, *J* = 10.0 Hz), 4.90 (dd, 1H, *J* = 9.7 and 9.6 Hz), 4.32 (m, 1H), 4.07 (t, 1H, *J* = 9.7 Hz), 3.69–3.64 (m, 2H), 3.51 (m, 1H), 3.39–3.24 (m, 4H), 2.44 (ddd, 1H, *J* = 13.3, 4.4 and 4.4 Hz), 2.15 (s, 3H), 2.10 (s, 3H), 2.09 (s, 3H), 1.64 (ddd, 1H, *J* = 12.8 Hz, each); ^13^C (125 MHz, CDCl_3_): *δ* 171.0, 169.9, 169.8, 98.9, 78.6, 74.1, 73.4, 72.1, 69.7, 69.6, 62.9, 58.7, 57.7, 51.0, 31.8, 20.9, 20.7, 20.5; HRMS (ESI-TOF) calculated for C_18_H_24_N_12_NaO_9_ [M + Na]: 575.1687, observed: 575.1680.

*5,3′,4′-Tri-O-acetyl-1,3,2′,6′-tetra-azidoneamine* (**15**). Compound **15** was synthesized from **12** as described for **14** above. **12** (0.13 g, 0.22 mmol) gave 98 mg (87%) of the product (**15**) as a white foam. ^1^H-NMR (500 MHz, CDCl_3_): *δ* 5.46 (dd, 1H, *J* = 10.7 and 9.3 Hz), 5.26 (d, 1H, *J* = 3.8 Hz), 5.09–5.04 (m, 2H), 4.48 (ddd, 1H, *J* = 10.2, 4.8 and 2.8 Hz), 3.63–3.53 (m, 3H), 3.48 (m, 1H), 3.42–3.30 (m, 3H), 2.94 (d, 1H, *J* = 6.3 Hz), 2.41 (ddd, 1H, *J* = 13.3, 4.4 and 4.4 Hz), 2.20 (s, 3H), 2.10 (s, 3H), 2.08 (s, 3H), 1.55 (ddd, 1H, *J* = 12.3 Hz, each); ^13^C (125 MHz, CDCl_3_): *δ* 171.2, 170.0, 169.8, 98.7, 78.7, 76.2, 75.6, 69.8, 69.5, 69.3, 60.7, 60.3, 58.6, 50.7, 31.6, 20.9, 20.6, 20.6; HRMS (ESI-TOF) calculated for C_18_H_24_N_12_NaO_9_ [M + Na]: 575.1687, observed: 575.1668.

*5,6,3′-Tri-O-acetyl-1,3,2′,6′-tetra-azidoneamine* (**16**). Compound **16** was synthesized from **13** as described for **14** above. **13** (0.28 g, 0.47 mmol) gave 98 mg (75%) of the product (**16**) as a white foam. ^1^H-NMR (500 MHz, CDCl_3_): δ 5.28 (dd, 1H, *J* = 10.6 and 9.3 Hz), 5.18 (d, 1H, *J* = 3.8 Hz), 5.15 (t, 1H, *J* = 9.8 Hz), 4.95 (t, 1H, *J* = 10.1 Hz), 4.29 (m, 1H), 3.74 (ddd, 1H, *J* = 14.6, 10.1 and 4.6 Hz), 3.67 (t, 1H, *J* = 9.8 Hz), 3.67–3.54 (m, 4H), 3.34 (dd, 1H, *J* = 10.7 and 3.8 Hz), 3.09 (d, 1H, *J* = 6.8 Hz), 2.47 (ddd, 1H, *J* = 13.4, 4.6 and 4.6 Hz), 2.19 (s, 3H), 2.10 (s, 6H), 1.64 (ddd, 1H, *J* = 12.6 Hz, each); ^13^C (125 MHz, CDCl_3_): *δ* 172.0, 169.9, 169.6, 98.9, 78.5, 74.1, 73.6, 73.1, 72.0, 69.9, 60.7, 58.5, 57.6, 50.8, 49.2, 31.9, 21.0, 20.6, 20.5; HRMS (ESI-TOF) calculated for C_18_H_24_N_12_NaO_9_ [M + Na]: 575.1687, observed: 575.1676.

*1,3,2′,6′,2′′′,6′′′ Hexa-azido-neobiosamine-(β-1′′→3′) neamine* (**20**). To a mixture of **14** (70 mg, 0.13 mmol) and **6** (0.13 g, 0.19 mmol) in dry DCM (2.0 mL), trimethylsilyl trifluoromethanesulfonate (14 µL, 0.075 mmol) was added at −20 °C under N_2_. The resulting mixture was stirred for 2 h, then quenched with Et_3_N (100 µL) and purified by silica gel chromatography (10% EtOAc/DCM) to obtain peracetylated product (**17,** contaminated by traces from **6**). The residue **17** was then dissolved in dry MeOH (2 mL) and sodium methoxide solution (2 mL of 0.2 mol L^−1^ solution in MeOH) was added. After 1 h, the reaction solution was neutralized with a strong cation-exchange resin (Dowex H^+^ 50W), filtered and evaporated to dryness. The residue was purified by silica gel chromatography (10% MeOH/DCM) to obtain 56 mg (57%) of the product **20** as a white foam. ^1^H-NMR (500 MHz, CD_3_OH): δ 5.64 (d, 1H, *J* = 3.7 Hz), 5.13 (s, 1H), 5.21 (d, 1H, *J* = 1.8 Hz), 4.64 (dd, 1H, *J* = 7.7 and 4.3 Hz), 4.27 (d, 1H, *J* = 4.3 Hz), 4.23-4.17 (m, 2H), 4.02 (ddd, 1H *J* = 8.3, 4.7 and 1.9 Hz), 3.94 (t, 1H, *J* = 3.4 Hz), 3.85-3.78 (m, 2H), 3.74 (dd, 1H, *J* = 12.1 and 3.7 Hz), 3.68-3.64 (m, 2H), 3.54-3.34 (m, 10H), 3.26 (t, 1H, *J* = 9.4 Hz), 2.26 (ddd, 1H, *J* = 12.9, 4.4 and 4.4 Hz), 1.42 (ddd, 1H, *J* = 12.3 Hz, each); ^13^C (125 MHz, CD_3_OH): *δ* 110.0, 99.7, 99.3, 83.3, 81.3, 81.2, 78.0, 77.9, 76.3, 75.8, 74.0, 73.1, 71.2, 71.1, 69.7, 64.8, 61.9, 61.9, 61.9, 60.8, 52.8, 52.5, 33.2; HRMS (ESI-TOF) calculated for C_23_H_34_N_18_NaO_13_ [M + Na]: 793.2450, observed: 793.2427.

*1,3,2′,6′,2′′′,6′′′ Hexa-azido-neobiosamine-(β-1′′→6) neamine* (**21**). Compound **21** was synthesized from **15** (94 mg, 0.17 mmol) as described for **20** (from **14**) above. 64 mg (49%) of **21** was obtained as a white foam. ^1^H-NMR (500 MHz, CD_3_OH): δ 5.56 (d, 1H, *J* = 3.8 Hz), 5.46 (d, 1H, *J* = 1.0 Hz), 5.10 (d, 1H, *J* = 1.8 Hz), 4.46 (dd, 1H, *J* = 7.3 and 4.5 Hz), 4.24 (dd, 1H, *J* = 4.5 Hz and 1.0 Hz), 4.20–4.13 (m, 2H), 4.00 (ddd, 1H, *J* = 8.3, 4.7 and 1.9 Hz), 3.94 (t, 1H, *J* = 3.4 Hz), 3.87-3.83 (m, 2H), 3.78 (dd, 1H, *J* = 11.9 and 6.4 Hz), 3.68-3.61 (m, 3H), 3.55–3.61 (m, 7H), 3.40–3.35 (m, 2H), 3.18 (dd, 1H, *J* = 10.5 and 3.8 Hz), 2.32 (m, 1H), 1.51 (m, 1H); ^13^C (125 MHz, CD_3_OH): *δ* 109.2, 100.2, 99.8, 83.6, 81.8, 81.7, 78.2, 77.6, 75.7, 74.6, 73.4, 72.7, 72.6, 71.3, 69.7, 65.0, 64.4, 61.9, 60.7, 60.3, 52.7, 52.5, 33.2; HRMS (ESI-TOF) calculated for C_23_H_34_N_18_NaO_13_ [M + Na]: 793.2450, observed: 793.2478.

*1,3,2′,6′,2′′′,6′′′ Hexa-azido-neobiosamine-(β-1′′→4′) neamine* (**22**). Compound **22** was synthesized from **16** (70 mg, 0.13 mmol) as described for **20** (from **14**) above. 67 mg (69%) of **22** was obtained as a white foam. ^1^H-NMR (500 MHz, CD_3_OH): *δ* 5.63 (d, 1H, *J* = 3.8 Hz), 5.13 (d, 1H, *J* = 1.8 Hz), 4.99 (d, 1H, *J* = 1.1 Hz), 4.57-4.55 (m, 1H), 4.19-4.16 (m, 3H), 4.03 (ddd, 1H, *J* = 8.5, 4.4 and 1.9 Hz), 3.96-3.91 (m, 2H), 3.82 (dd, 1H, *J* = 12.1 and 2.5 Hz), 3.72 (dd, 1H, *J* = 12.1 and 4.1 Hz), 3.69-3.63 (m, 2H), 3.57 (dd, 1H, *J* = 13.4 and 2.4 Hz), 3.52-3.38 (m, 7H), 3.36 (dd, 1H, *J* = 13.0 and 4.5 Hz), 3.26 (t, 1H, *J* = 9.4 Hz), 3.19 (dd, 1H, *J* = 10.5 and 3.8 Hz), 2.24 (ddd, 1H, *J* = 12.8, 4.3 and 4.3 Hz), 1.41 (ddd, 1H, *J* = 12.3 Hz, each); ^13^C (125 MHz, CD_3_OH): *δ* 110.0, 99.7, 99.5, 83.7, 81.3, 81.2, 78.0, 78.0, 76.4, 75.8, 74.6, 71.9, 71.3, 70.9, 69.7, 64.3, 62.1, 61.9, 61.9, 61.0, 52.5, 52.1, 33.3; HRMS (ESI-TOF) calculated for C_23_H_34_N_18_NaO_13_ [M + Na]: 793.2450, observed: 793.2467.

RP HPLC analysis of **20**–**23**. The purity of the isolated products (**20**–**23**) was further confirmed by an RP HPLC analysis. An aliquot of each compound was dissolved in a mixture of acetonitrile and water (1:9, *v/v*) and injected to an analytical RP (C18, 250 × 4.6 mm, 5 µm) column. Gradient elutions from 10% to 80% acetonitrile in H_2_O over 30 min. (chromatograms a–c/Figure 1: purified products **20**-**23**) and from 10% to 60% acetonitrile over 35 min. (chromatogram d/Figure 1: a mixture of **20**–**23**, flow rate of 1.0 mL min^−1^, and detection at λ = 220 nm were used.

*Neobiosamine (β-1′′→3′) neamine* (**23**). Trimethylphosphine (1 mol L^−1^ Me_3_P in toluene, 0.99 mL, 1.0 mmol) was added to a mixture of **20** (51 mg, 0.066 mmol) in water-dioxane (1:4, *v*/*v*, 2.0 mL). The mixture was stirred at ambient temperature for 4 h under nitrogen and then concentrated ammonia (1.0 mL) was added. After overnight reaction the mixture was evaporated to dryness. The crude product was purified by an ion exchange chromatography (Dowex 1 × 2 200 anion exchanger resin, activated by 2 mol L^−1^ NaOH, eluent carbon dioxide free H_2_O) to give 30 mg (73%) of the product (**23**) as a yellowish powder. ^1^H-NMR (500 MHz, D_2_O): *δ* 5.36 (d, 1H, *J* = 3.9 Hz), 5.24 (d, 1H, *J* = 0.8 Hz), 4.99 (d, 1H, *J* = 1.8 Hz), 4.62 (dd, 1H, *J* = 7.1 and 4.7 Hz), 4.40 (m, 1H), 4.23 (m, 1H), 4.05 (dd, 1H, *J* = 3.2 Hz, both), 3.97 (m, 1H), 3.90 (dd, 1H, *J* = 12.5 and 2.8 Hz), 3.86–3.78 (m, 2H), 3.71–3.66 (m, 2H), 3.54 (t, 1H, *J* = 9.2 Hz), 3.42 (t, 1H, *J* = 9.5 Hz), 3.32 (t, 1H, *J* = 9.3 Hz), 3.17 (t, 1H, *J* = 9.6 Hz), 3.08–3.01 (m, 3H), 2.94–2.84 (m, 4H), 2.75 (m, 1H), 2.00 (ddd, 1H, *J* = 12.9, 4.1 and 4.1 Hz), 1.23 (ddd, 1H, ddd, *J* = 12.1 Hz, each); ^13^C (125 MHz, D_2_O): *δ* 108.4, 100.3, 99.1, 86.2, 83.2, 81.5, 77.4, 76.0, 75.6, 75.2, 73.1, 72.2, 70.6, 69.8, 68.5, 60.3, 54.6, 52.6, 50.3, 49.3, 41.5, 41.1, 35.0; HRMS (ESI-TOF) calculated for C_23_H_47_N_6_O_13_ [M + H]: 615.3196, observed: 615.3221.

*Neobiosamine (β-1′′→6) neamine* (**24**). **24** was synthesized as described for **23** above. 61 mg (0.079 mmol) of **21** gave 29 mg (58%) of **24** as a yellowish powder. ^1^H-NMR (500 MHz, D_2_O): *δ* 5.34 (d, 1H, *J* = 3.9 Hz), 5.25 (s, 1H), 4.98 (d, 1H, *J* = 1.8 Hz), 4.62 (dd, 1H, *J* = 7.4 and 4.7 Hz), 4.38 (m, 1H), 4.20 (m, 1H), 4.04 (t, 1H, *J* = 3.2 Hz), 3.99 (m, 1H), 3.90 (dd, 1H, *J* = 12.6 and 2.6 Hz), 3.82–3.74 (m, 2H), 3.68–3.57 (m, 3H), 3.35–3.29 (m, 3H), 3.10–3.03 (m, 3H), 2.94 (dd, 1H, *J* = 13.5 and 4.1 Hz), 2.88–2.75 (m, 4H), 2.03 (ddd, 1H, *J* = 12.9, 4.1 and 4.1 Hz), 1.26 (ddd, 1H, *J* = 12.4 Hz, each); ^13^C (125 MHz, D_2_O): *δ* 108.8, 100.4, 98.9, 85.9, 85.8, 81.3, 75.9, 75.4, 75.0, 73.4, 73.0, 72.4, 71.4, 70.5, 68.5, 60.2, 55.2, 52.6, 49.1, 48.9, 41.4, 41.0, 36.1; HRMS (ESI-TOF) calculated for C_23_H_47_N_6_O_13_ [M + H]: 615.3196, observed: 615.3192.

*Neobiosamine (β-1′′→4′) neamine* (**25**). **25** was synthesized as described for **23** above. 51 mg (0.066 mmol) of **22** gave 32 mg (83%) of **25** as a yellowish powder. ^1^H-NMR (500 MHz, D_2_O): *δ* 5.31 (d, 1H, *J* = 3.9 Hz), 5.09 (s, 1H), 4.97 (d, 1H, *J* = 1.6 Hz), 4.60 (dd, 1H, *J* = 7.0 and 4.6 Hz), 4.31 (m, 1H), 4.23 (m, 1H), 4.04 (m, 1H, 3.96-3.88 (m, 2H), 3.85–3.79 (m, 2H), 3.68–3.64 (m, 2H), 3.52 (dd, 1H, *J* = 9.2 Hz, both), 3.42 (t, 1H, *J* = 9.4 Hz), 3.30 (t, 1H, *J* = 9.2 Hz), 3.16 (t, 1H, *J* = 9.6 Hz), 3.04 -2.98 (m, 3H), 2.90–2.81 (m, 4H), 2.72 (m, 1H), 1.99 (m, 1H), 1.22 (ddd, 1H, *J* = 12.4 Hz, each); ^13^C (125 MHz, D_2_O): *δ* 108.1, 100.3, 99.2, 86.9, 81.5, 80.5, 77.4, 75.9, 75.7, 75.5, 73.0, 72.1, 71.9, 70.6, 68.5, 60.2, 54.9, 52.7, 50.3, 49.3, 41.2, 41.1, 35.5; HRMS (ESI-TOF) calculated for C_23_H_47_N_6_O_13_ [M + H]: 615.3196, observed: 615.3202.

*UV measurements.* Melting curves (absorbance vs. temperature) were measured at 260 nm on a Perkin-Elmer Lambda 35 UV/Vis spectrophotometer equipped with a multiple cell holder and a Peltier temperature controller. An internal thermometer was additionally used to verify the validity of the target temperature. The temperature was changed from 10 to 80 °C at a rate of 0.2 °C min. *T*_m_ values were determined as the maximum of the first derivate of the melting curve. 

## 4. Conclusions

A straightforward synthesis of glycosidic (1′′→6, 3′ and 4′) site isomers (**23**–**25**) of neomycin B has been described. The synthesis includes (1) acid-catalyzed thiolysis of neomycin azide (**3**) to obtain useful intermediates (**4** and **5**) for both the glycosyl donor (**6**) and the acceptors (**14**–**16**), and (2) selective diacetylation of neamine azide (**7**), which after simple protecting group manipulation (i.e., levulinoylation, acetylation and delevulinoylation) afforded the glycosyl acceptors (**14**–**16**). Glycosylation between the neobiosamine donor (**6**) and triacetylated neamine azide acceptors (**14**–**16**) was carried out in standard condition to give the azide masked pseudo tetrasaccharides (**21**–**22**) in acceptable yields. The azide masks were removed by Staudinger reaction to obtain the desired glycosidic site isomers of neomycin B (**23**–**25**). The potential of these aminoglycosides (**23**–**25**) to stabilize DNA and RNA triple helix models was studied by UV-melting profile analysis. In each case a marked stabilization was observed. The variable neobiosamine site (6-O, 3′-O or 4′-O) on the neamine core did not show marked difference in the stability compared to neomycin B. It may be concluded that the carbohydrate core and spatial orientation of the amino groups (i.e., spatial orientation of l and d-neosamines provided by the variable glycoside site between neobioasamine and neamine) on it do not play a marked enough role to result in discrimination between the affinities of the site isomers.

## Figures and Tables

**Table 1:** UV-melting experiments (Tm/°C).

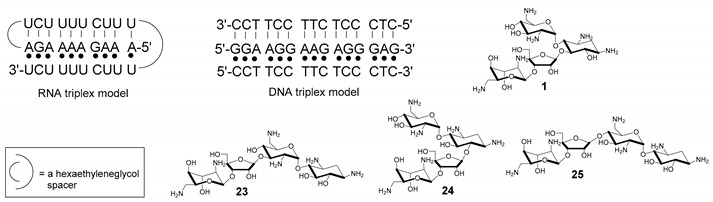		
	RNA Triplex Model	
**AG**	***T*_m_^3^(°C) pH 6.0**	***T*_m_^2^(°C) pH 6.0**
none	41.9	60.1
**1** (5 eq)	51.2 (+9.3)	68.0 (+7.9)
**23** (5 eq)	53.2 (+11.3)	66.7 (+6.6)
**24** (5 eq)	52.5 (+10.6)	67.7 (+7.6)
**25** (5 eq)	51.4 (+9.5)	68.0 (+7.9)
**AG**	***T*_m_^3^(°C) pH 7.0**	***T*_m_^2^(°C) pH 7.0**
none	22.6	60.1
**1** (5 eq)	29.4 (+6.9)	62.0 (+1.9)
**23** (5 eq)	30.4 (+7.8)	61.2 (+1.1)
**24** (5 eq)	30.4 (+7.8)	61.6 (+1.5)
**25** (5 eq)	29.4 (+6.9)	61.9 (+1.8)
**DNA Triplex Model**		
**AG**	***T*_m_^3^(°C) pH 6.0**	***T*_m_^2^(°C) pH 6.0**
none	25.5	54.6
**1** (5 eq)	40.1 (+14.6)	56.3 (+1.7)
**23** (5 eq)	38.1 (+12.6)	55.5 (+0.9)
**24** (5 eq)	36.6 (+11.1)	56.3 (+1.7)
**25** (5 eq)	38.6 (+13.1)	55.3 (+0.7)
**1** (10 eq)	44.1 (+18.6)	56.3 (+1.7)
**23** (10 eq)	41.3 (+15.8)	55.9 (+1.3)
**24** (10 eq)	39.4 (+13.9)	56.9 (+2.3)
**25** (10 eq)	42.9 (+17.4)	56.5 (+1.9)

Δ*T*_m_-values in parentheses. Error limits for each *T*_m_-value (an average of three temperature ramps) were less than 1 °C. *T*_m_^3^ and *T*_m_^2^ correspond to melting values of the triplex and duplex, respectively.

## Supplementary Materials

The NMR spectral data for all new compounds are available online.
