# Supplementary material for: Synthesis of Glycosidic (β-1′′→6, 3′ and 4′) Site Isomers of Neomycin B and Their Effect on RNA and DNA Triplex Stability
Source: Molecules. 2019 Feb 6;24(3):580. doi: 10.3390/molecules24030580 (PMC6385478; doi:10.3390/molecules24030580)
Supplement: Supplementary file 1 [file molecules-24-00580-s001.pdf]

Supporting Information to:

Synthesis of glycosidic (1''→3',4' and 6) site-isomers of neomycin B and their effect on RNA- and DNA-triplex stability

Lotta Granqvist, Ville Tähtinen and Pasi Virta*

Department of Chemistry, University of Turku, 20014 Turku, Finland

Contents

NMR spectra of **4**	**S2-S5**
NMR spectra of **6**	**S6-S7**
NMR spectra of **8**	**S8-S9**
NMR spectra of **9**+**10**	**S10-S12**
NMR spectra of **11**	**S13-S14**
NMR spectra of **12**	**S15-S16**
NMR spectra of **13**	**S17-S18**
NMR spectra of **14**	**S19-S20**
NMR spectra of **15**	**S21-S22**
NMR spectra of **16**	**S23-S24**
NMR spectra of **20**	**S25-S26**
NMR spectra of **21**	**S27-S28**
NMR spectra of **22**	**S29-S30**
NMR spectra of **23**	**S31-S32**
NMR spectra of **24**	**S33-S34**
NMR spectra of **25**	**S35-S36**

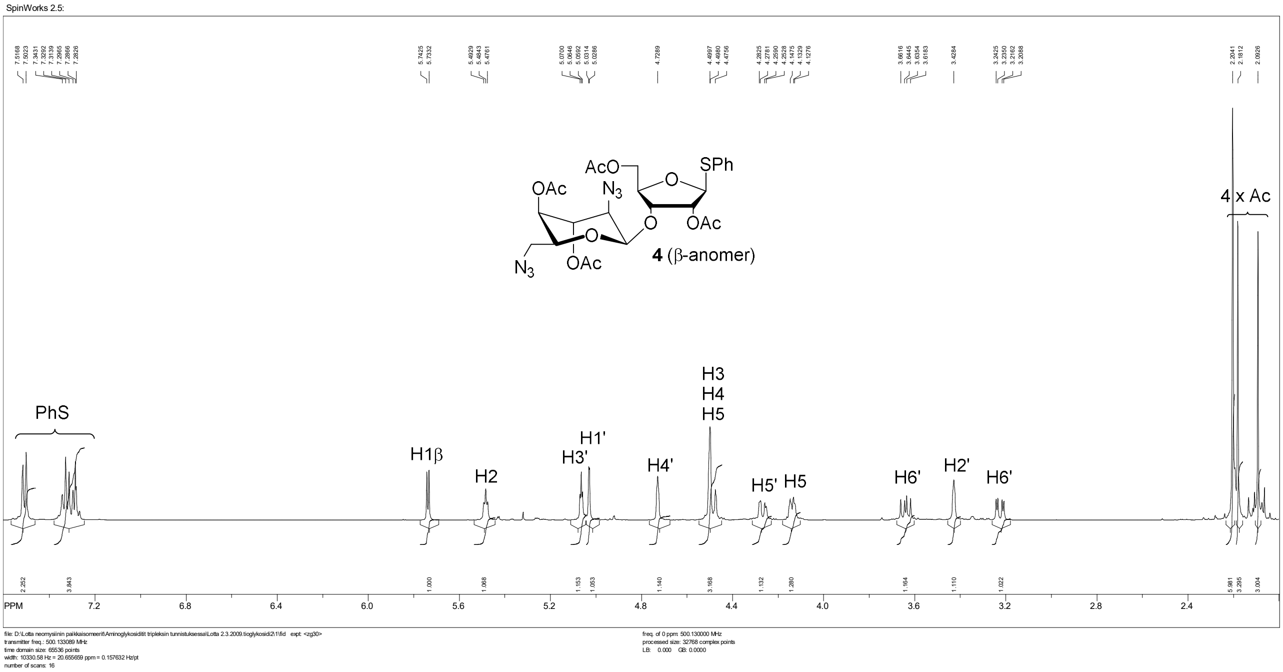

Figure S1. ^{1}H NMR (500 MHz, $CDCl_3$) spectrum of **4** (β-anomer).

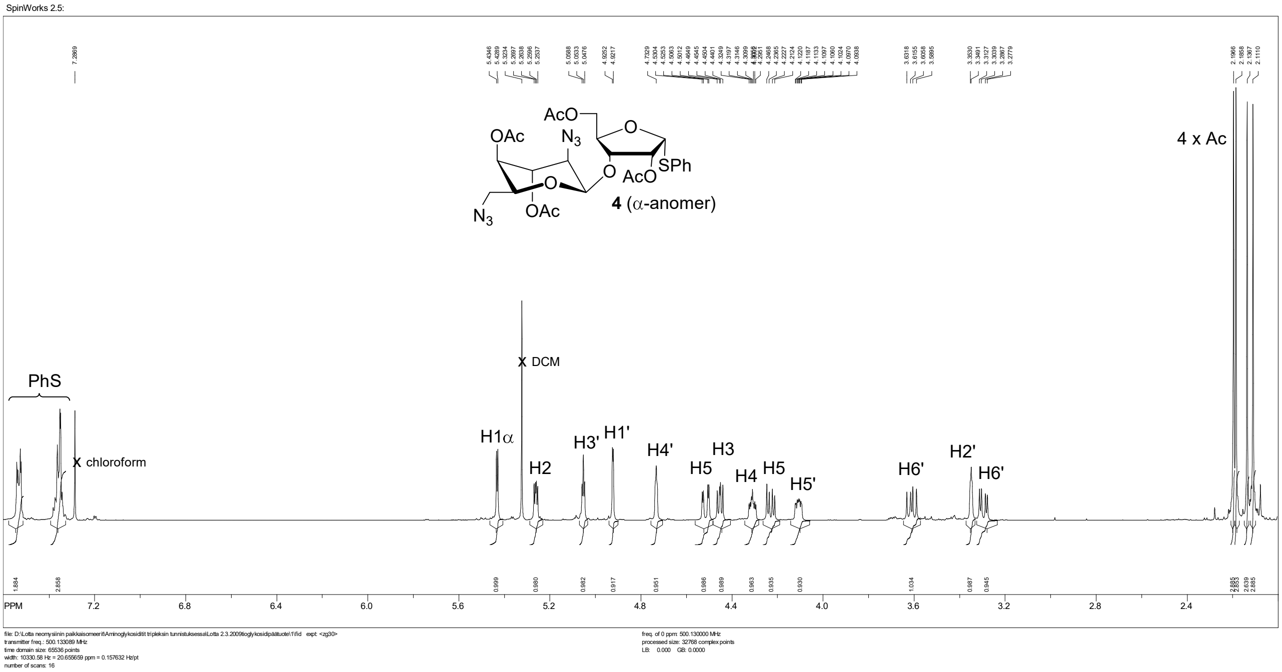

Figure S2. ^{1}H NMR (500 MHz, $CDCl_3$) spectrum of **4** (α-anomer).

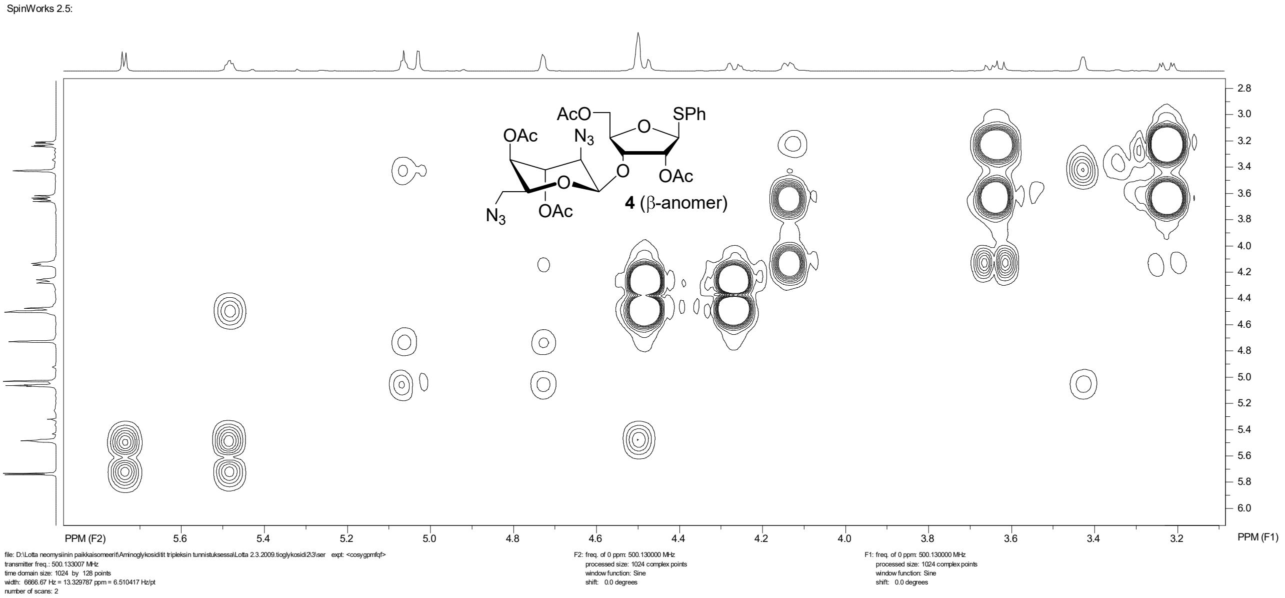

Figure S3. COSY spectrum of **4** (β-anomer).

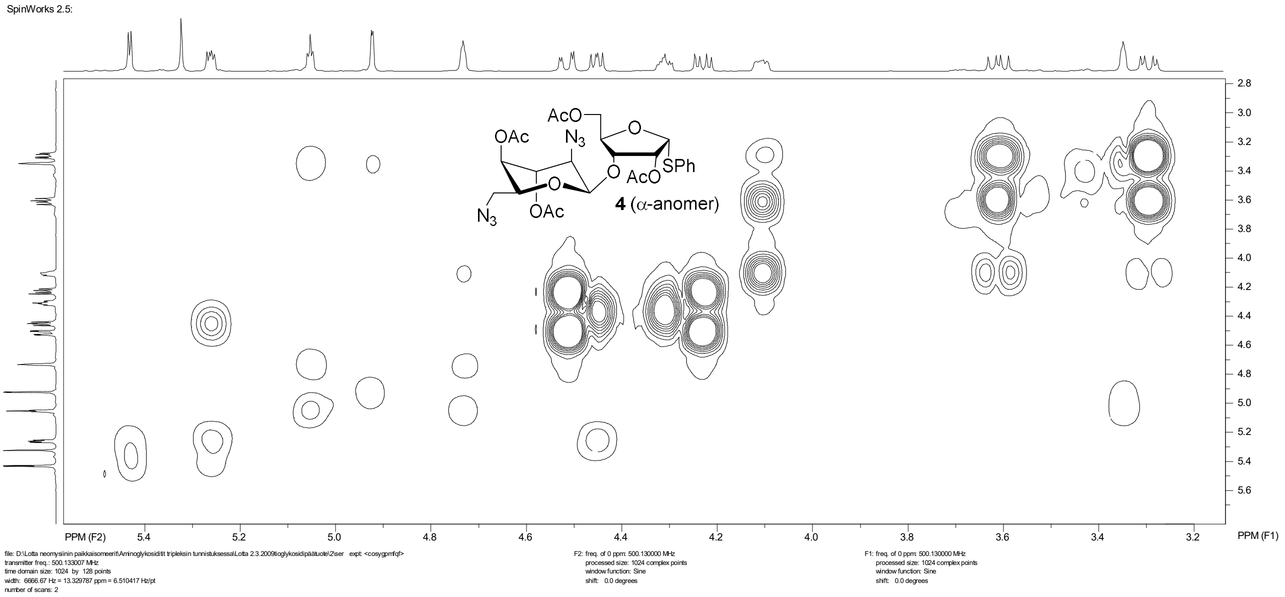

Figure S4. COSY spectrum of **4** (α-anomer).

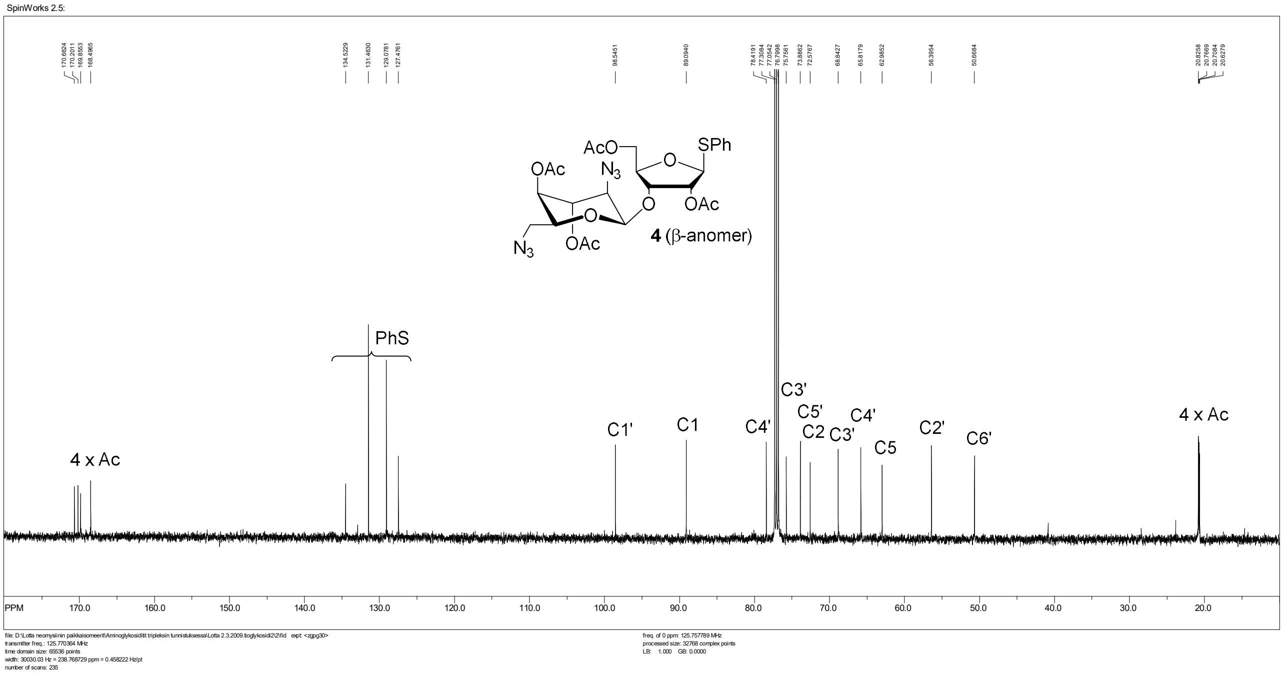

Figure S5. ^{13}C NMR (125 MHz, $CDCl_3$) spectrum of **4** (β-anomer).

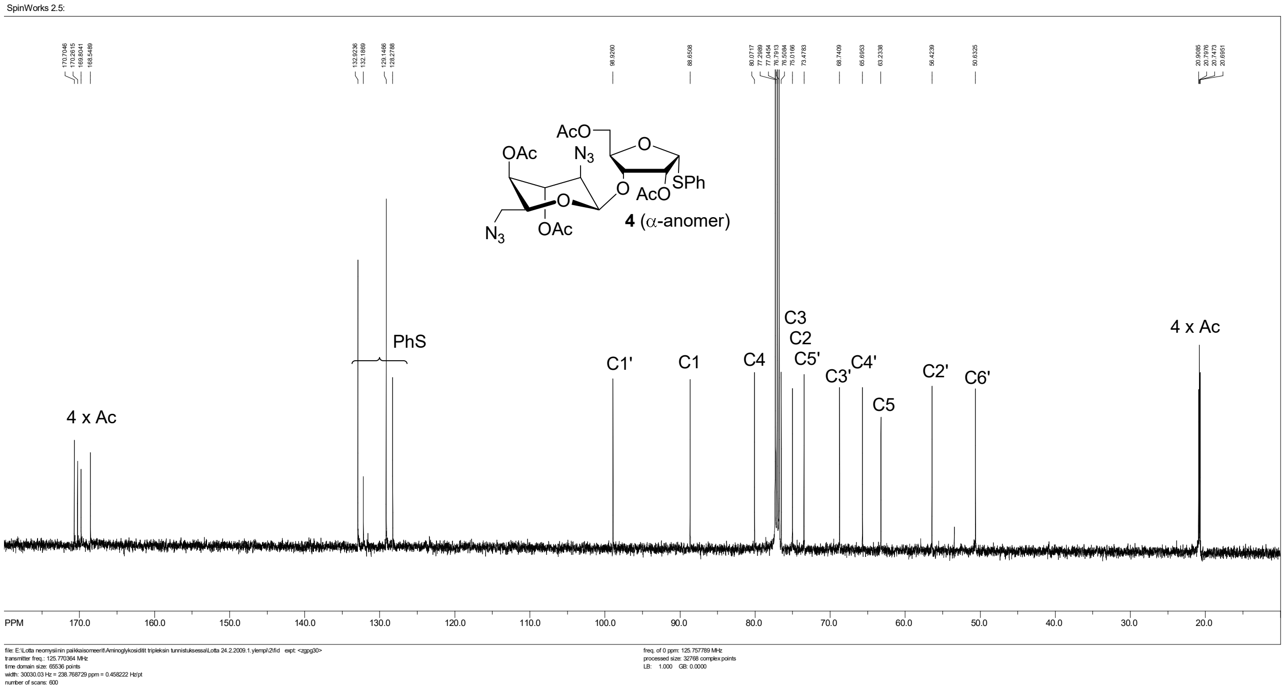

Figure S6. ^{13}C NMR (125 MHz, $CDCl_3$) spectrum of **4** (α-anomer).

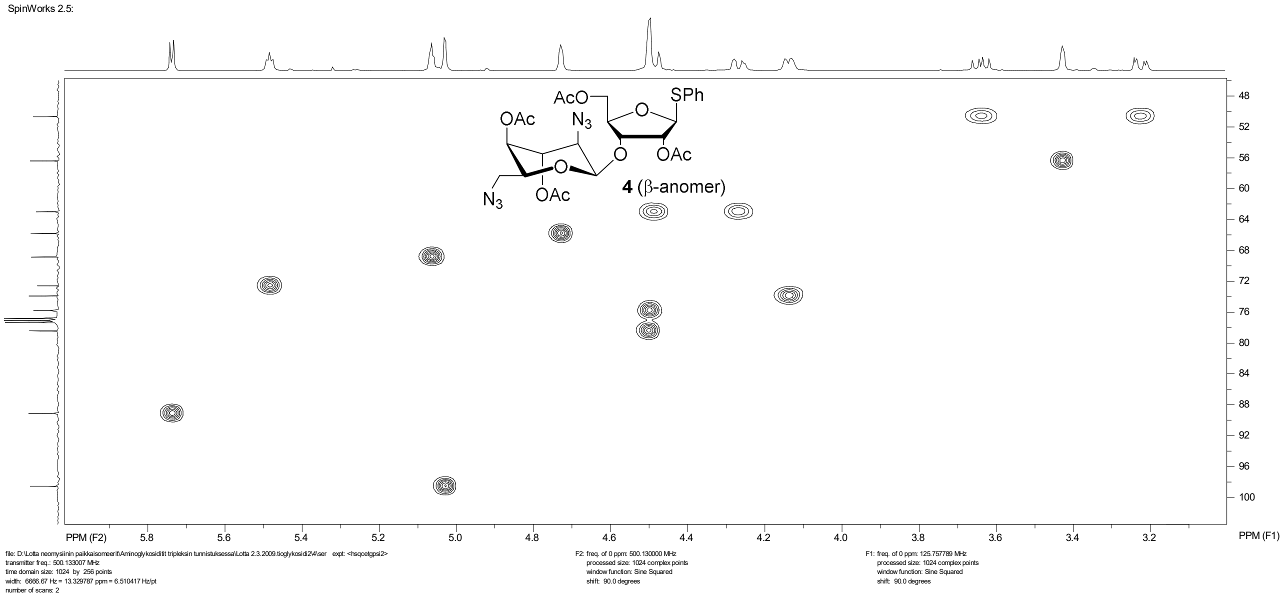

Figure S7. HSQC spectrum of **4** (β-anomer).

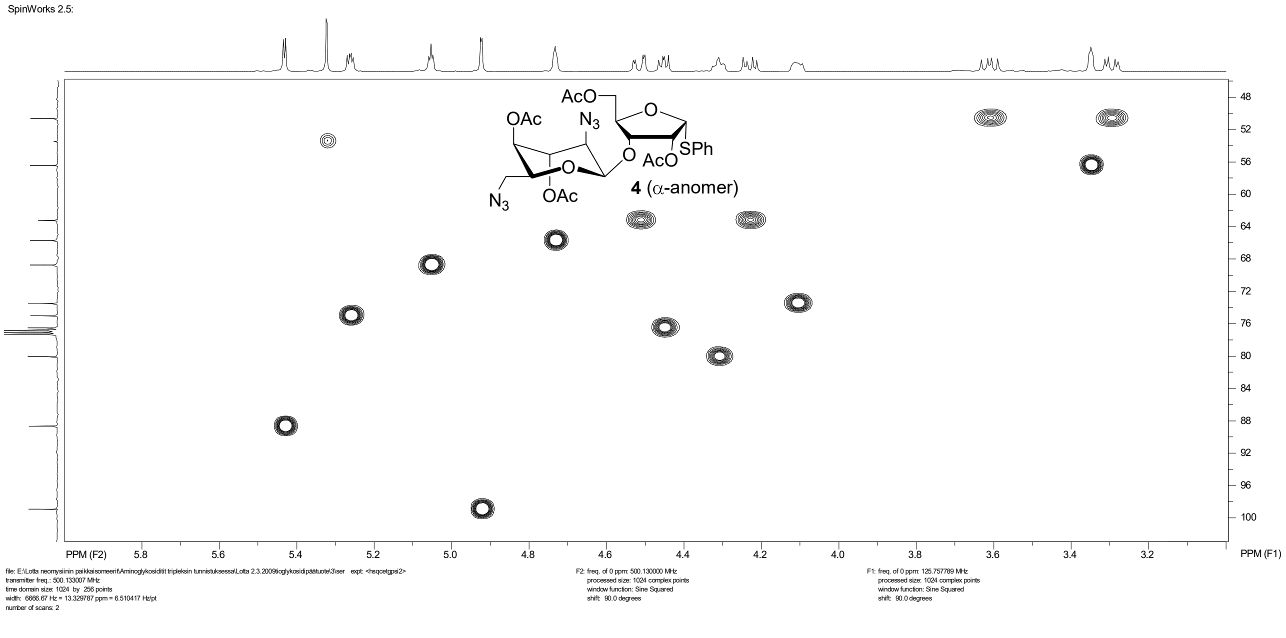

Figure S8. HSQC spectrum of **4** (α-anomer).

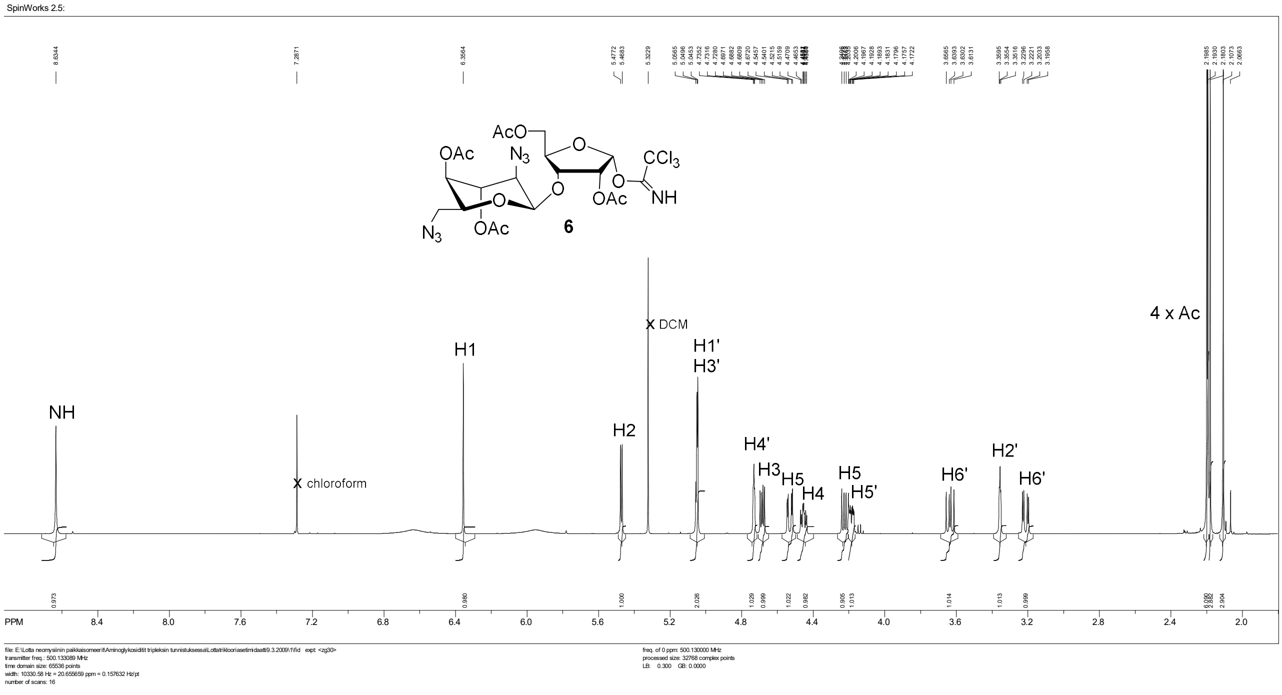

Figure S9. ^{1}H NMR (500 MHz, $CDCl_3$) spectrum of **6**.

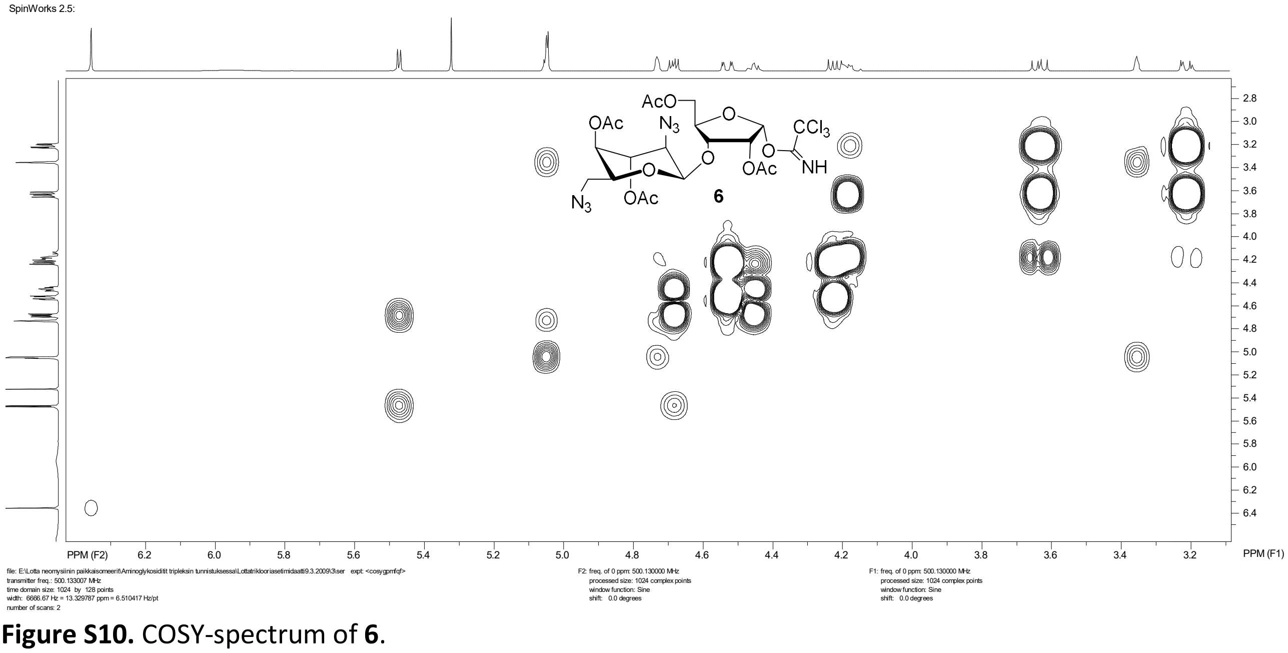

Figure S10. COSY-spectrum of **6**.

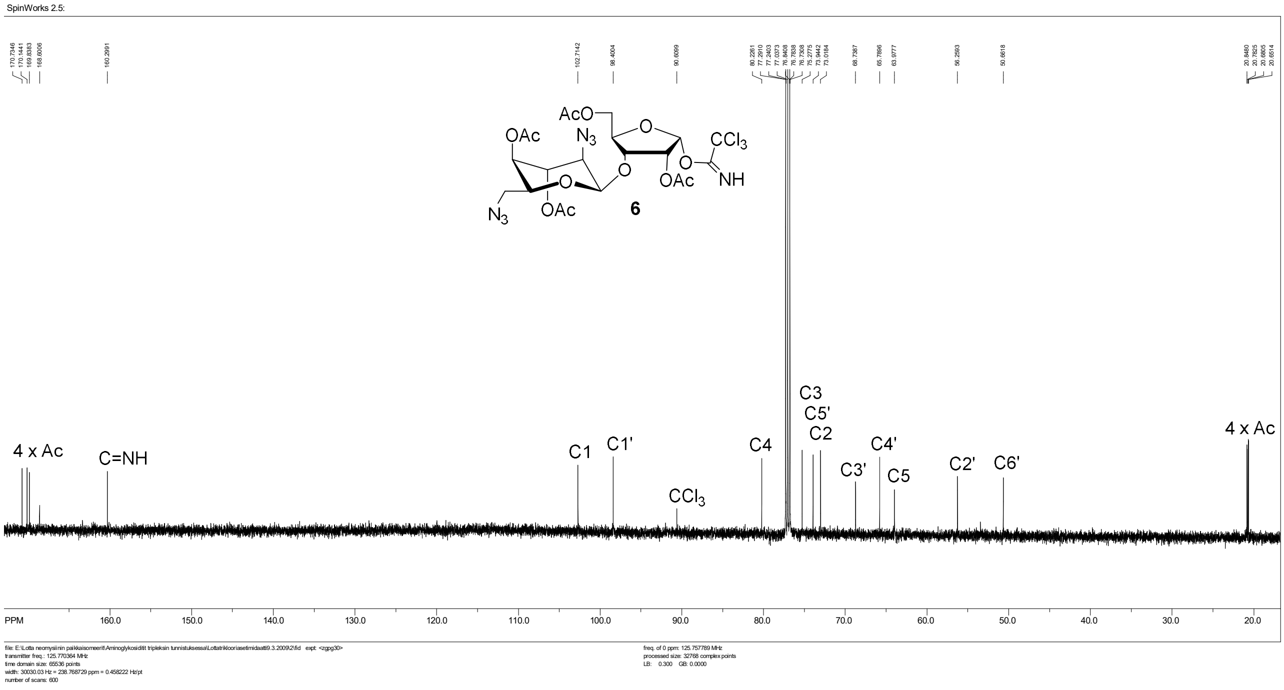

Figure S11. ^{13}C NMR (125 MHz, $CDCl_3$) spectrum of **6**.

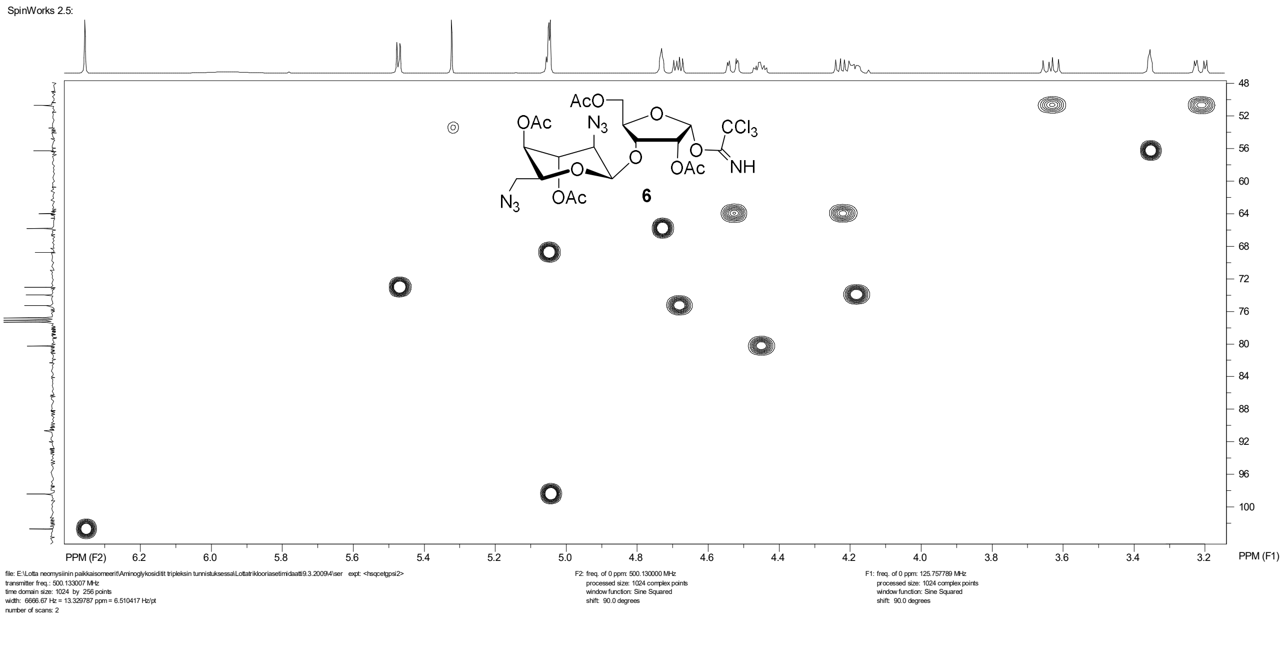

Figure S12. HSQC spectrum of **6**.

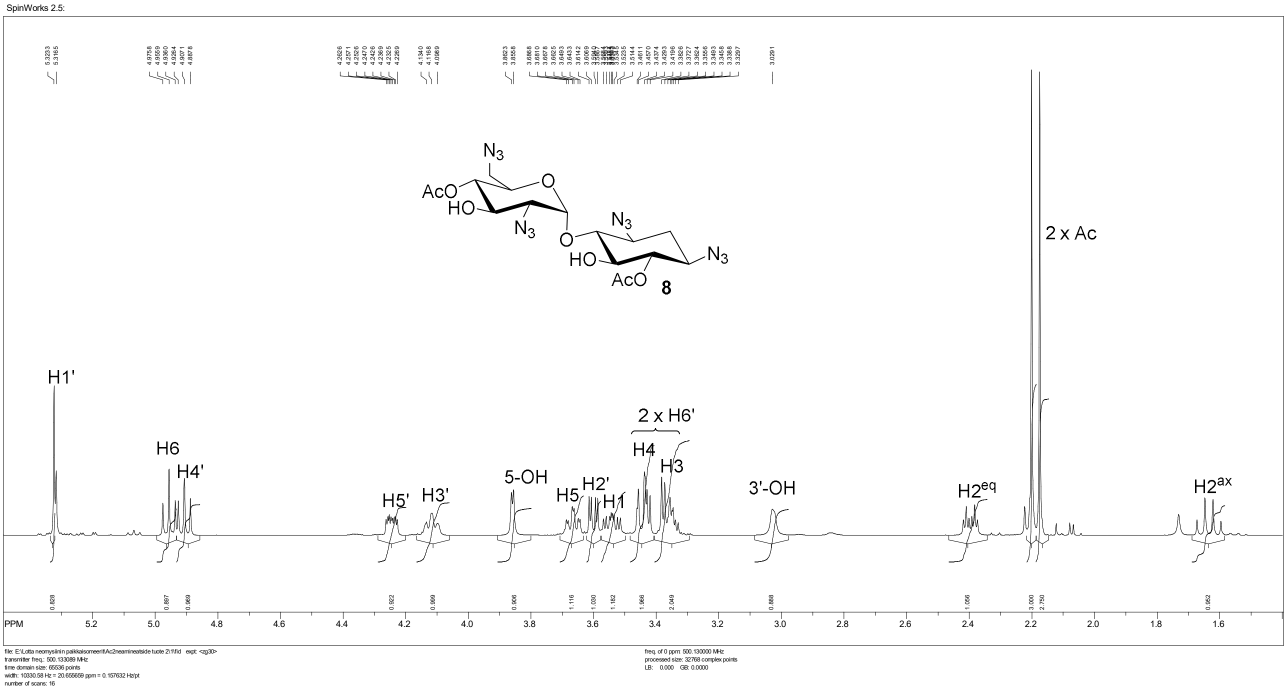

Figure S13. ^{1}H NMR (500 MHz, $CDCl_3$) spectrum of **8**.

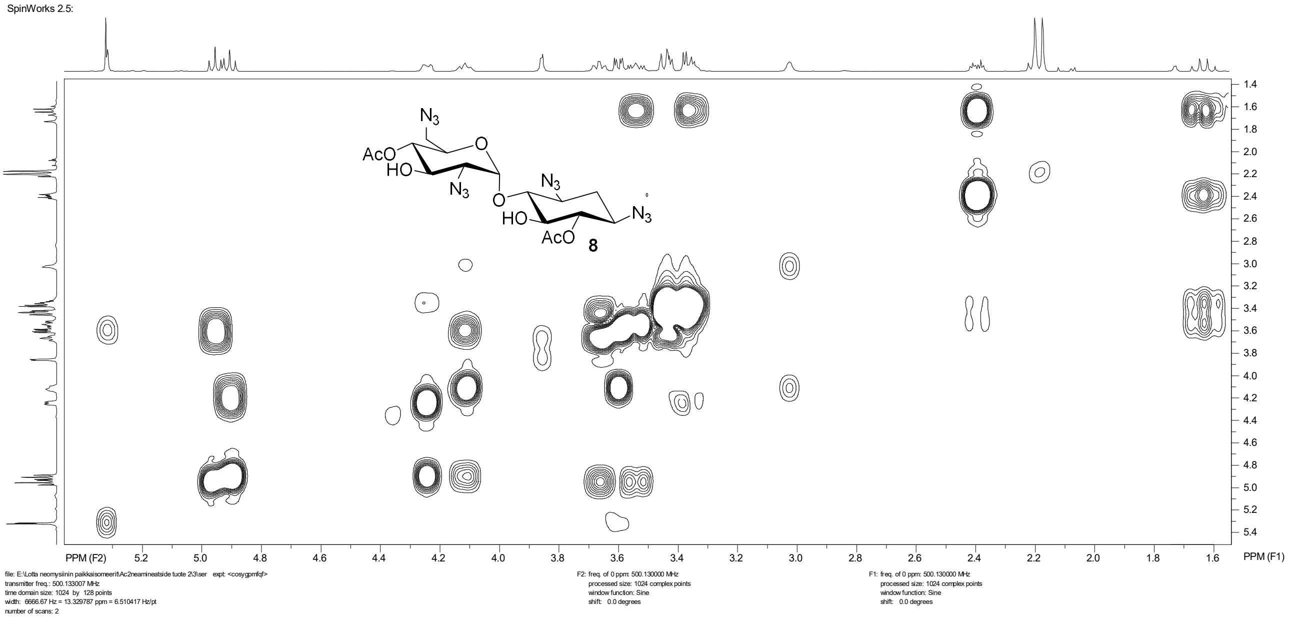

Figure S14. COSY spectrum of **8**.

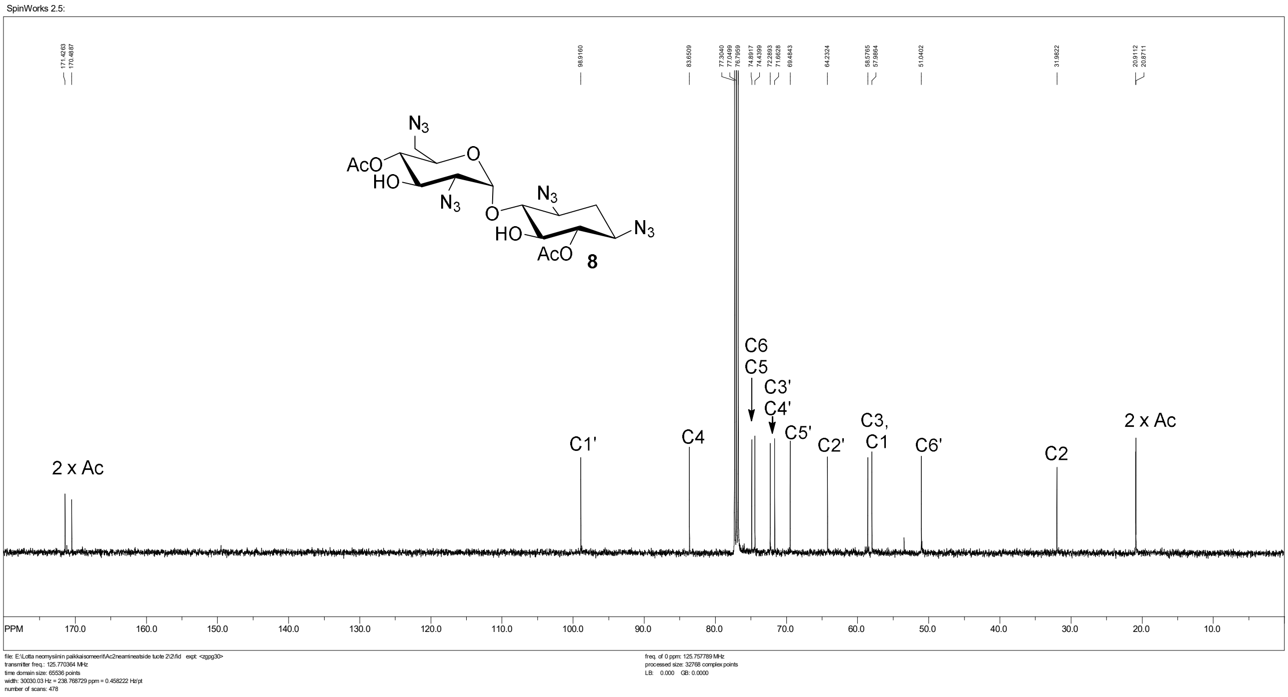

Figure S15. ^{13}C NMR (125 MHz, $CDCl_3$) spectrum of **8**.

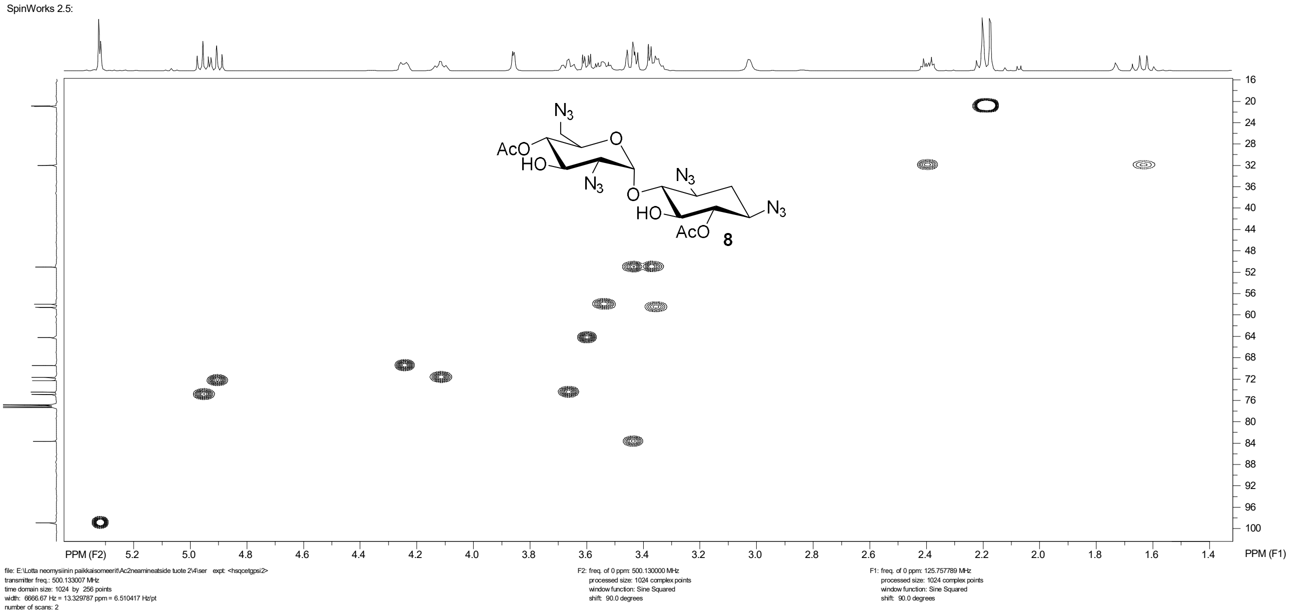

Figure S16. HSQC spectrum of **8**.

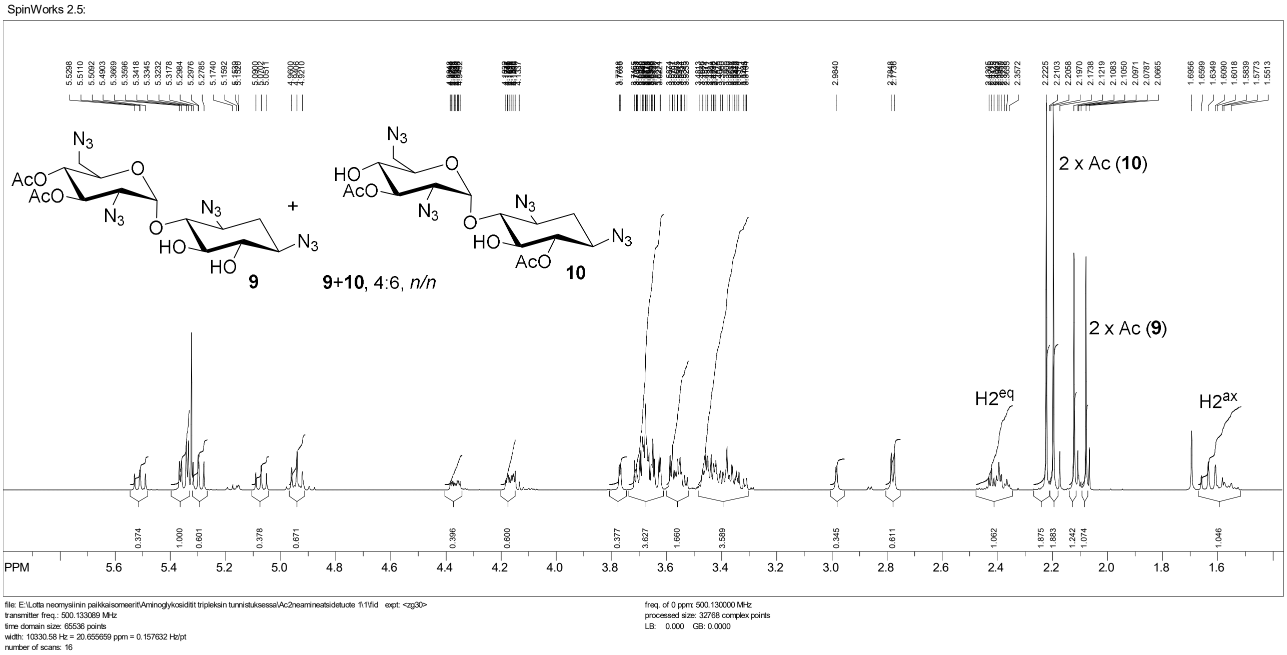

Figure S17. ^{1}H NMR (500 MHz, $CDCl_3$) spectrum of **9** + **10**.

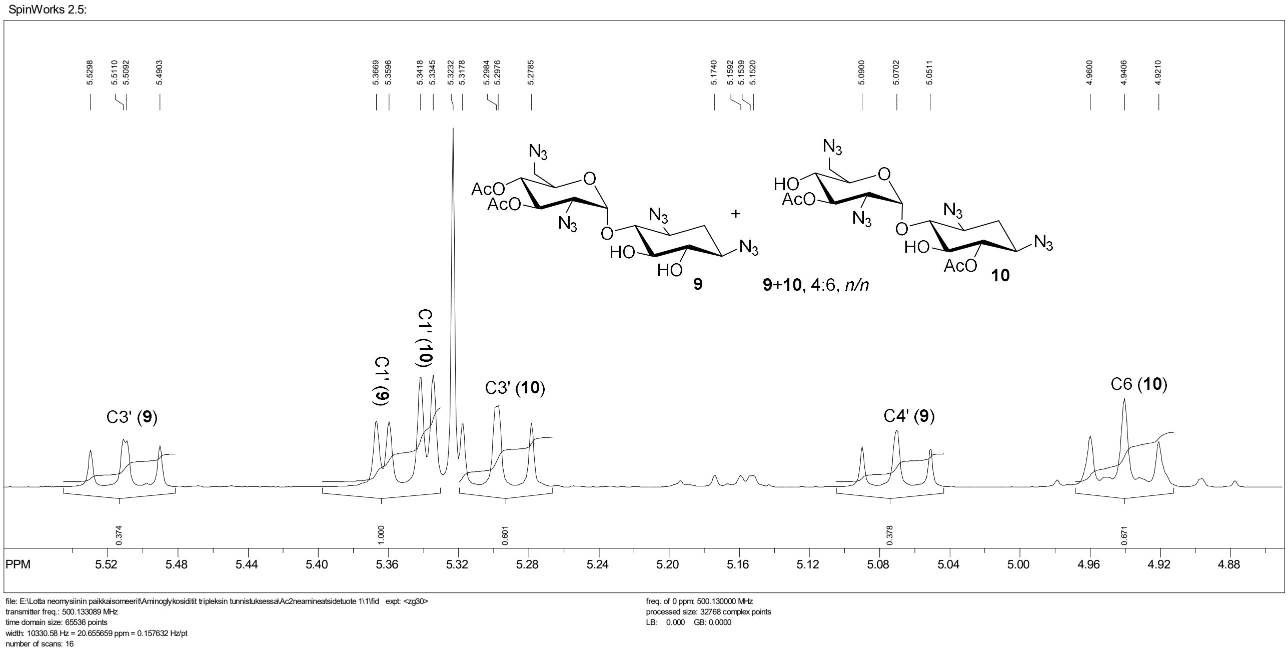

Figure S18. ^{1}H NMR (500 MHz, $CDCl_3$) spectrum of **9** + **10**.

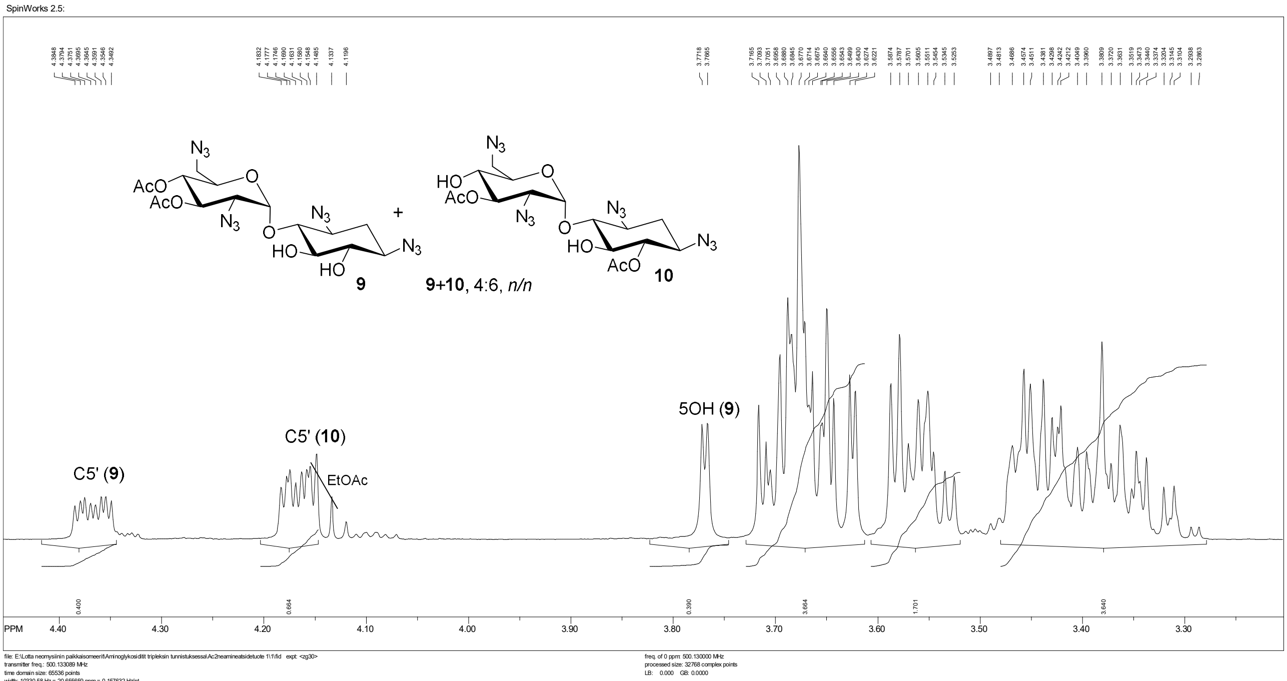

Figure S19. ^{1}H NMR (500 MHz, $CDCl_3$) spectrum of **9** + **10**.

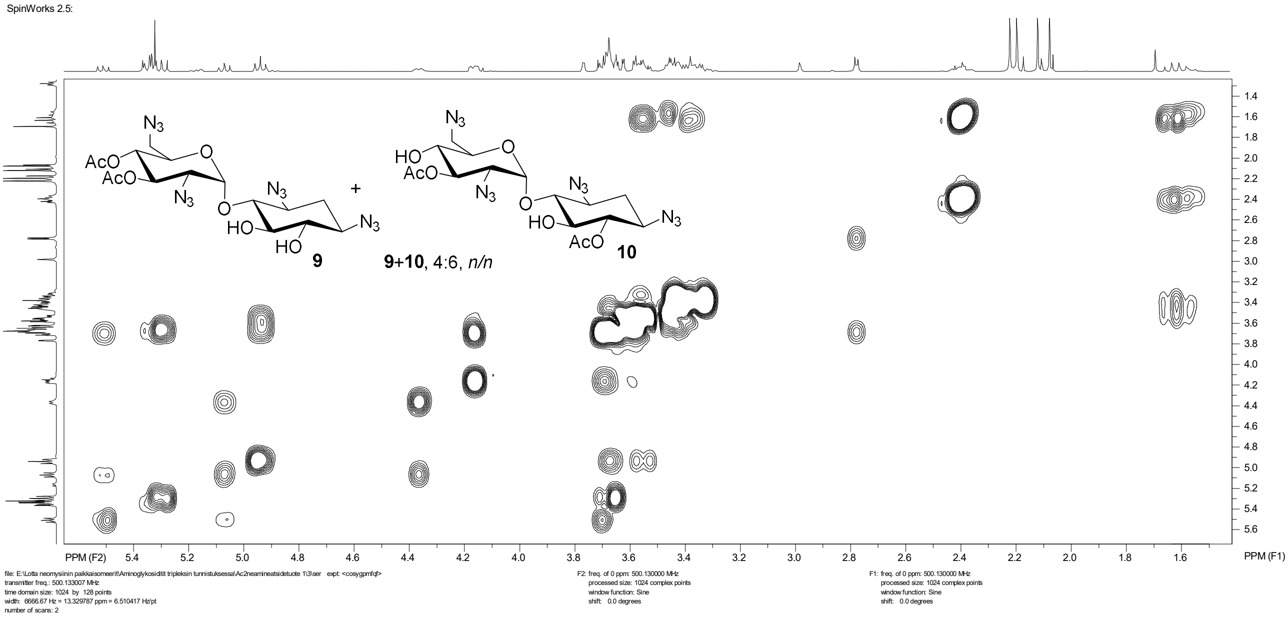

Figure S20. COSY spectrum of **9** + **10**.

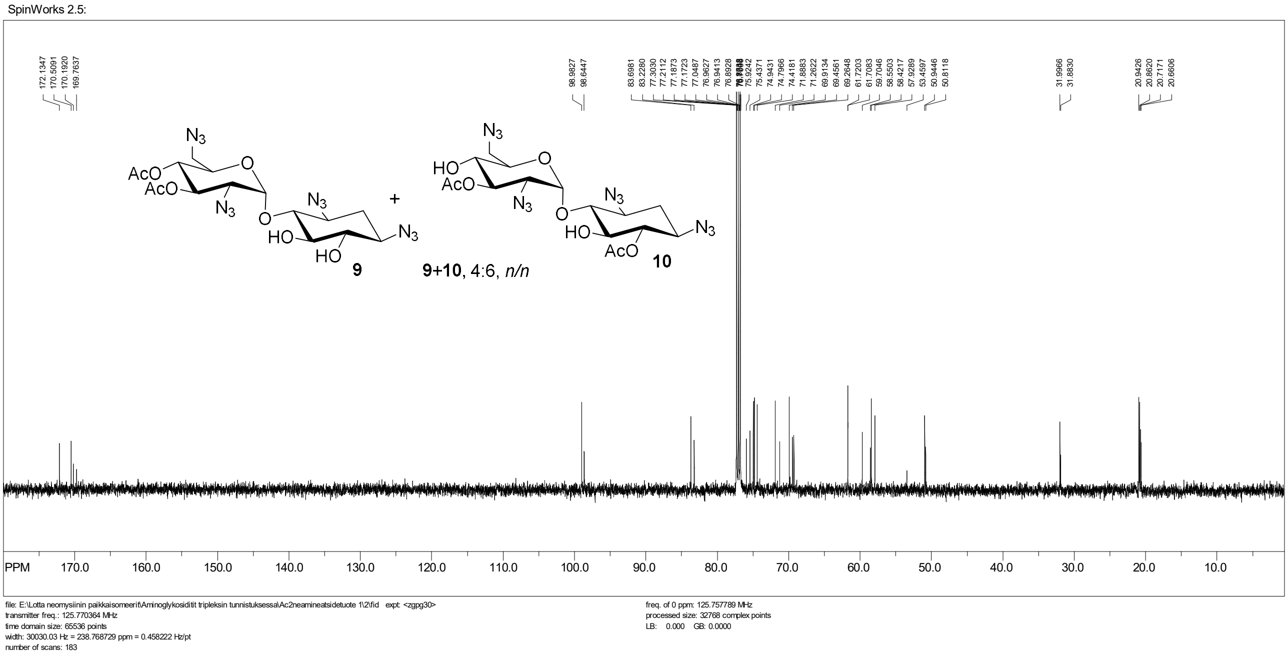

Figure S21. ^{13}C NMR (125 MHz, $CDCl_3$) spectrum of **9** + **10**.

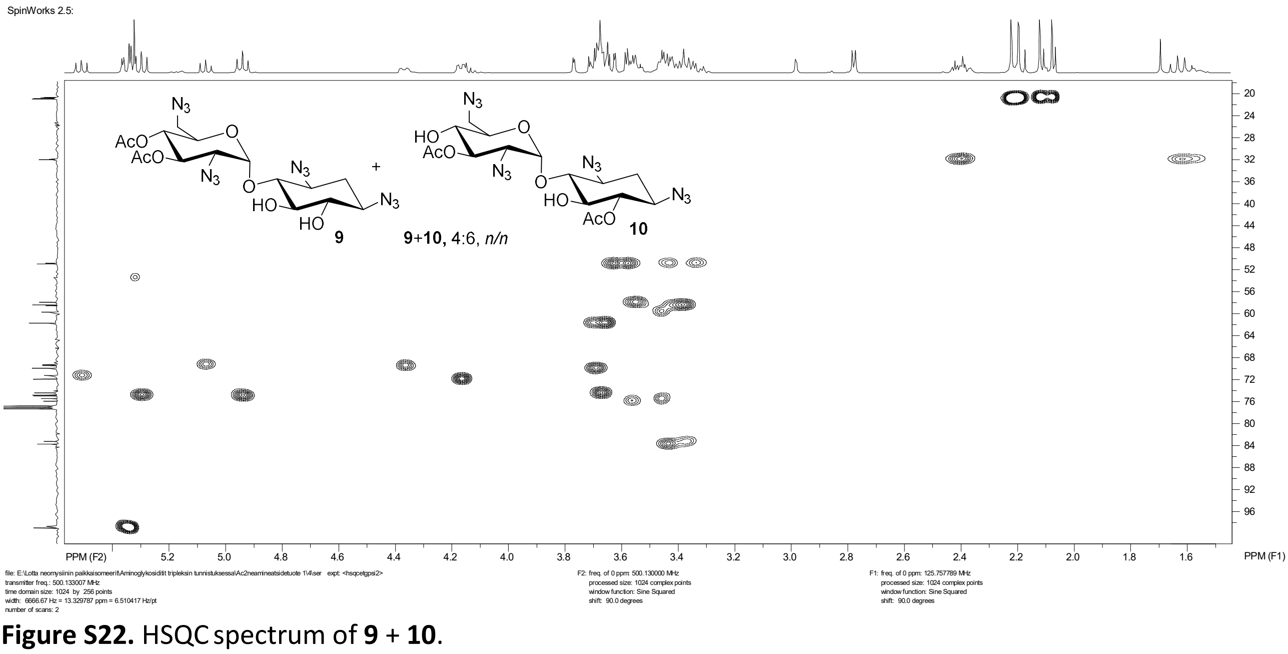

Figure S22. HSQC spectrum of **9** + **10**.

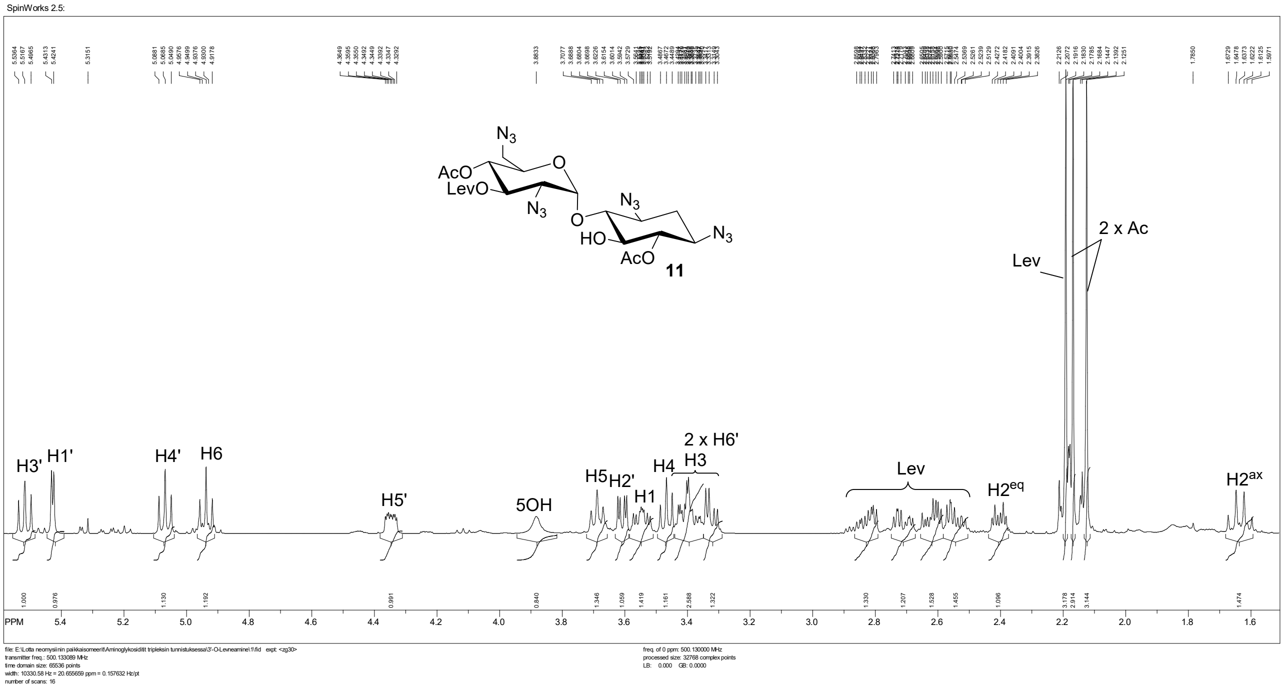

Figure S23. ^{1}H NMR (500 MHz, $CDCl_3$) spectrum of **11**.

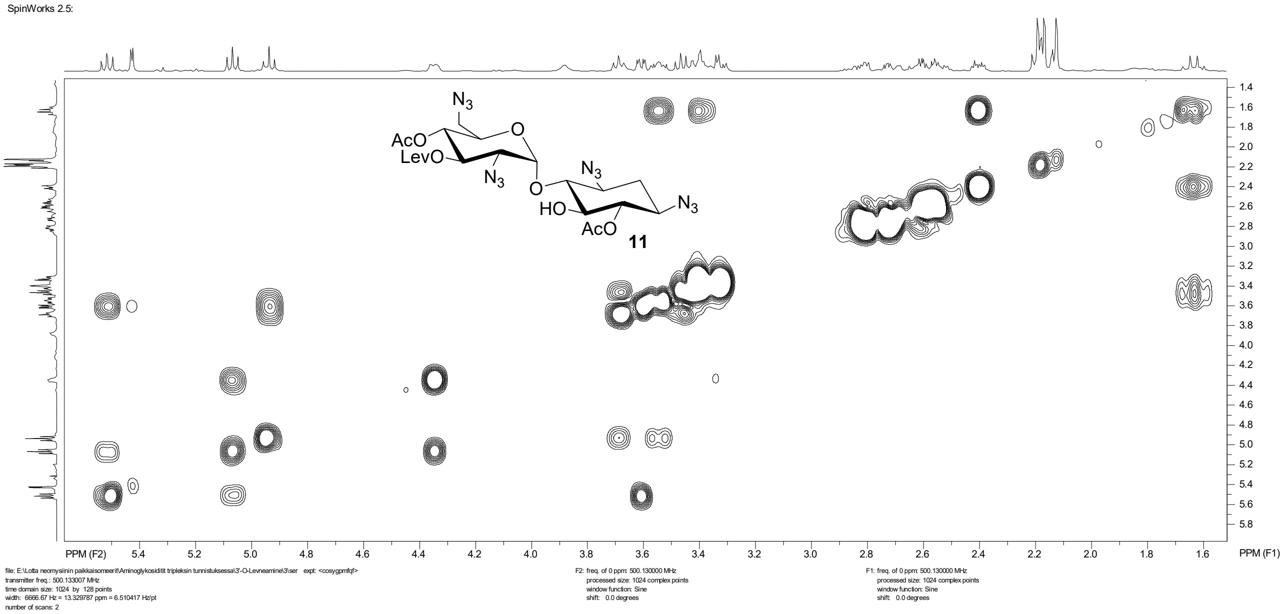

Figure S24. COSY pectrum of **11**.

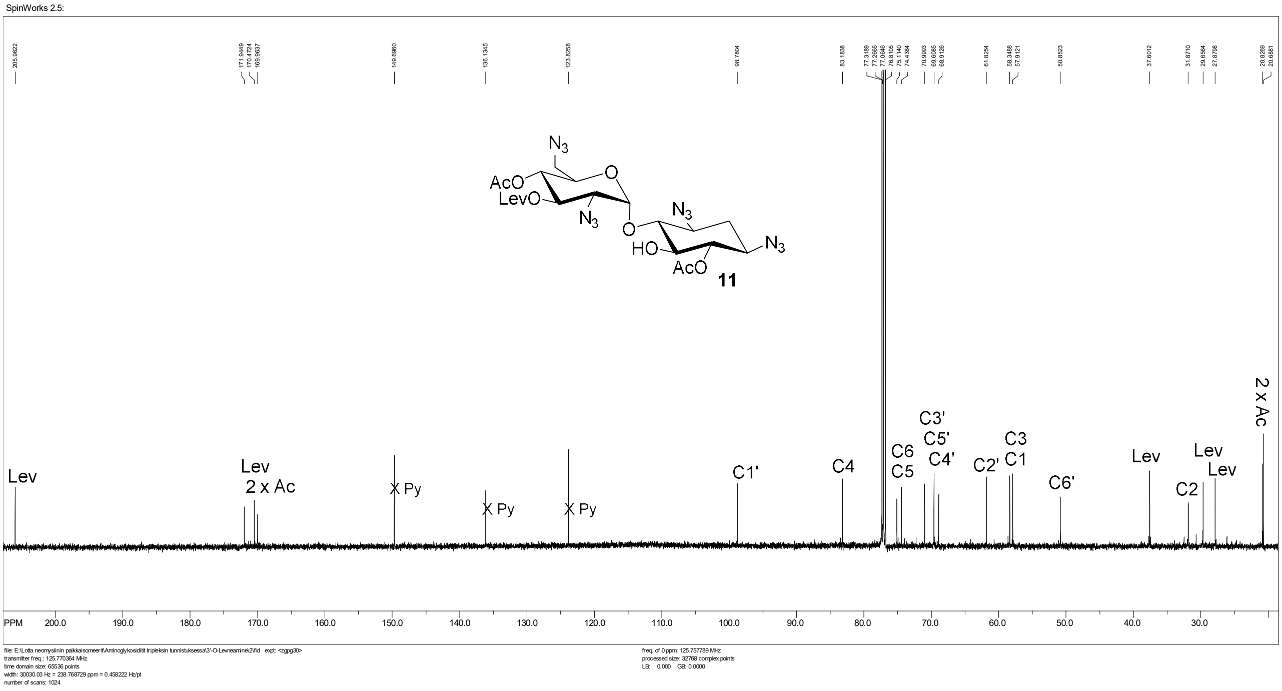

Figure S25. ^{13}C NMR (125 MHz, $CDCl_3$) spectrum of **11**.

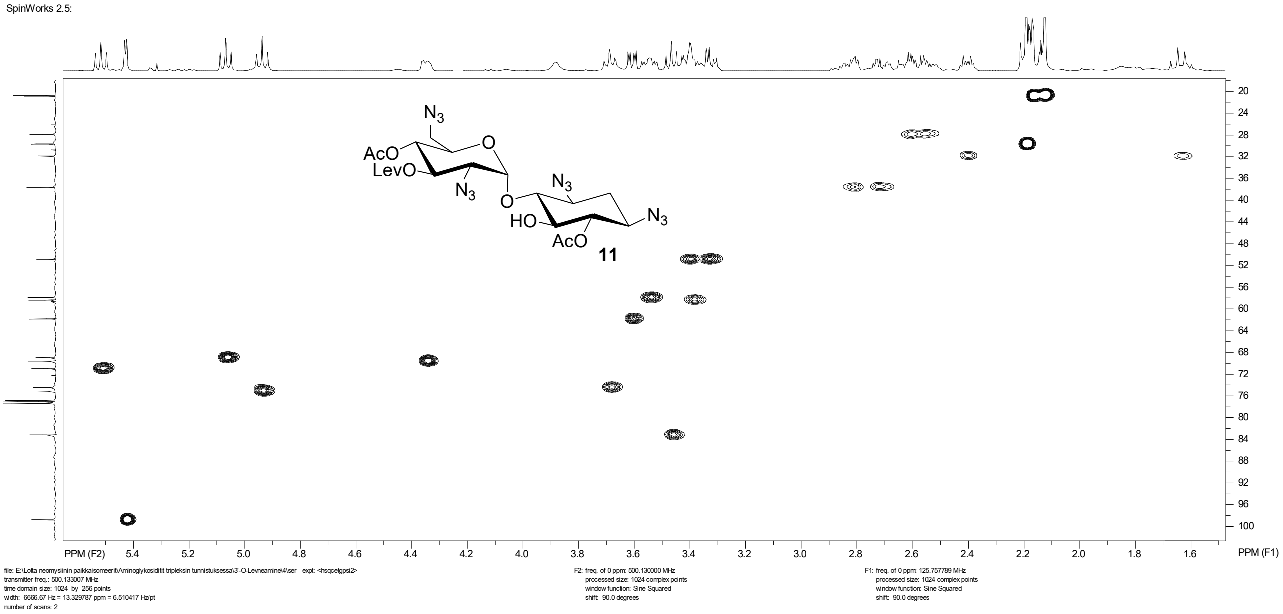

Figure S26. HSQC spectrum of **11**.

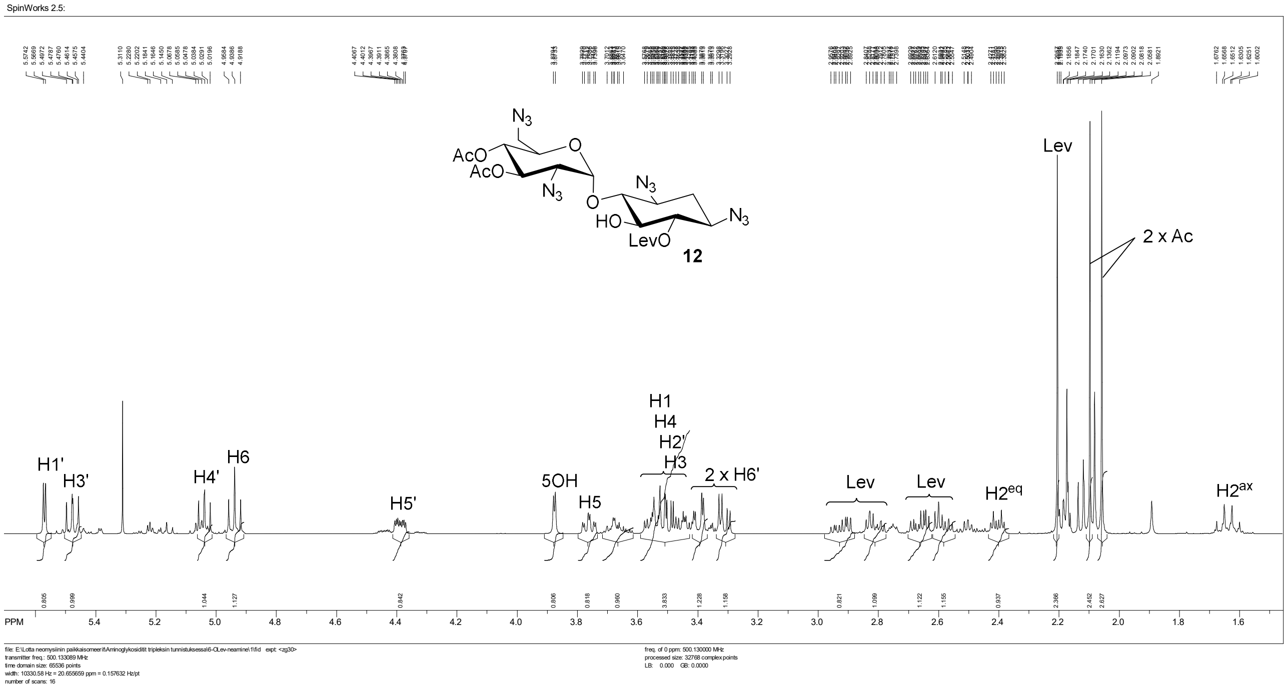

Figure S27. ^{1}H NMR (500 MHz, $CDCl_3$) spectrum of **12**.

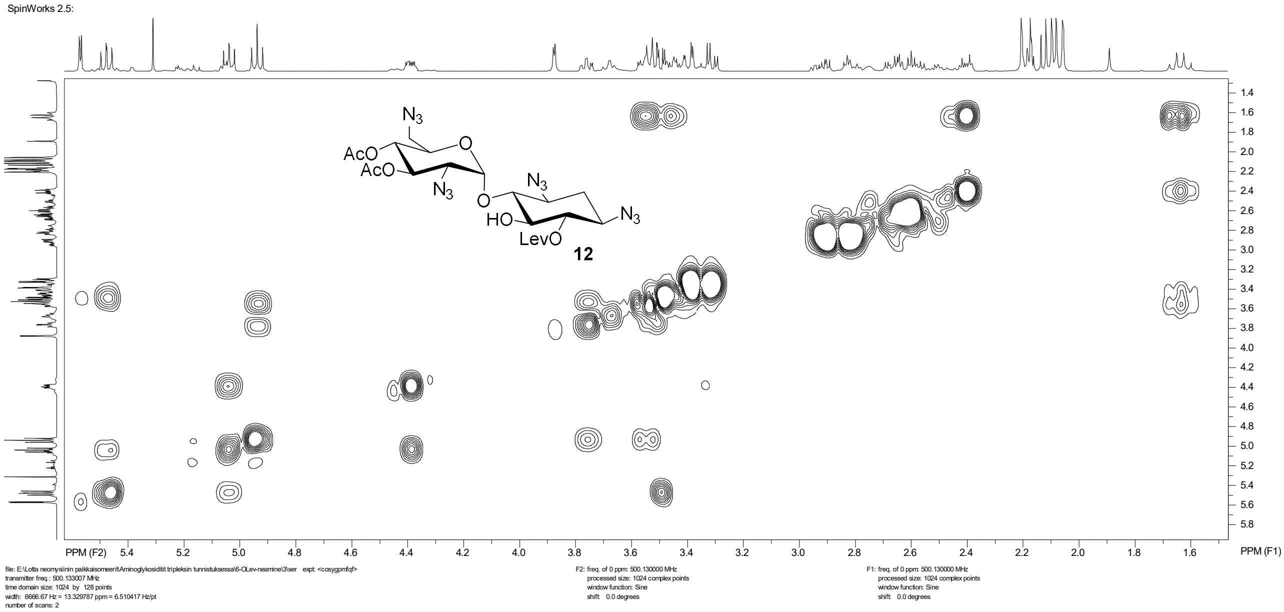

Figure S28. COSY spectrum of **12**.

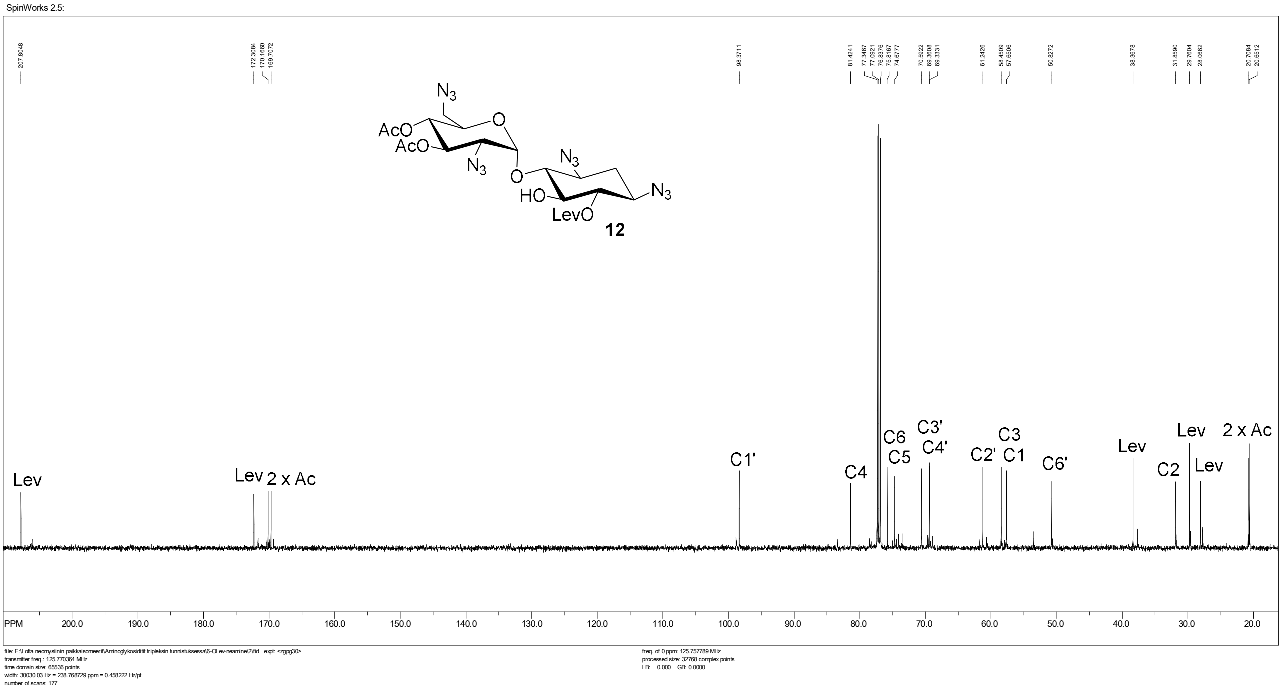

Figure S29. ^{13}C NMR (125 MHz, $CDCl_3$) spectrum of **12**.

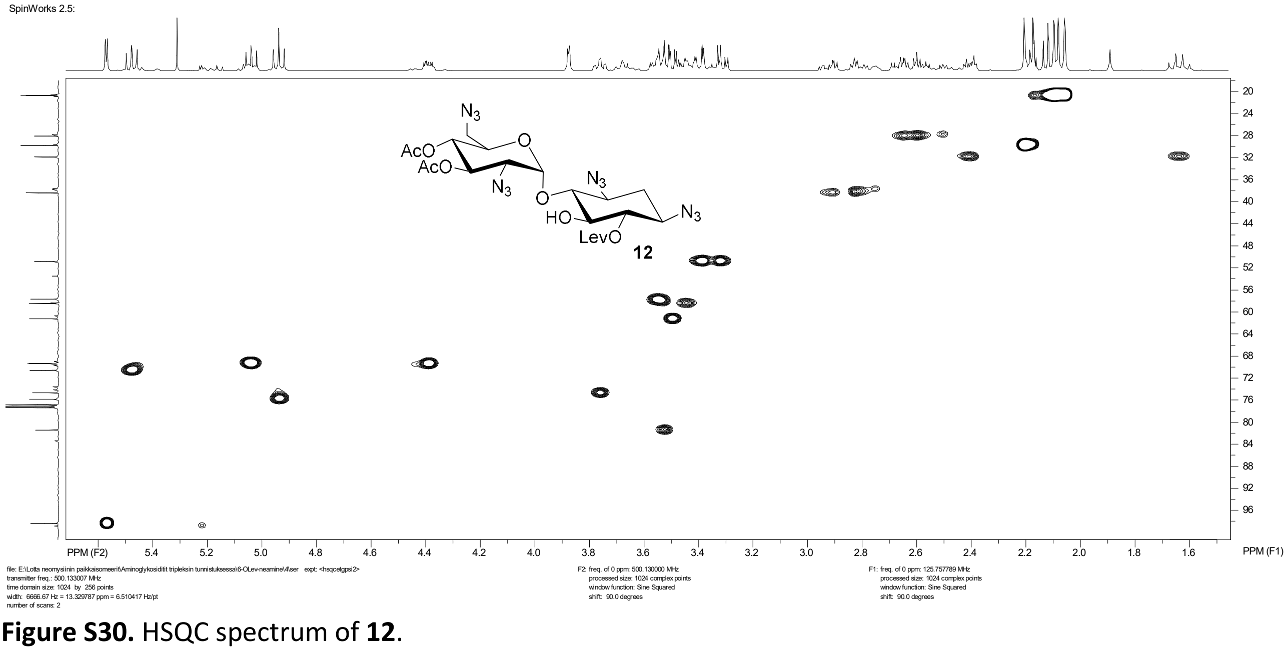

Figure S30. HSQC spectrum of **12**.

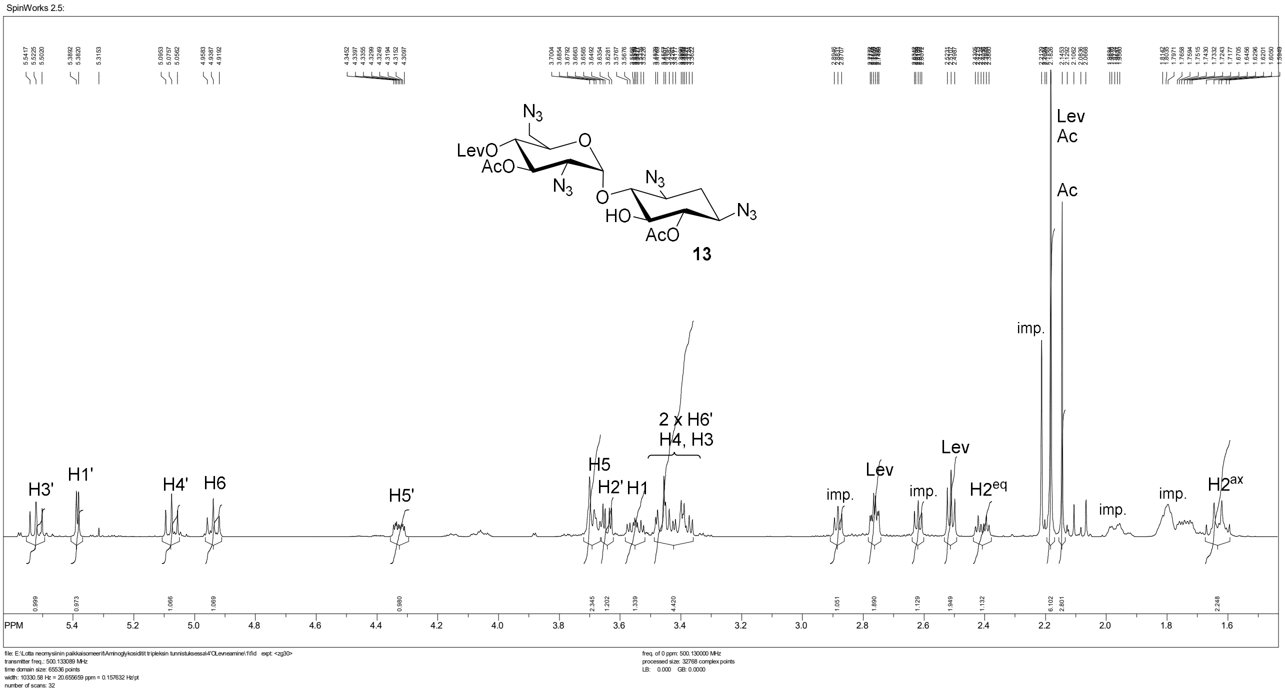

Figure S31. ^{1}H NMR (500 MHz, $CDCl_3$) spectrum of **13**.

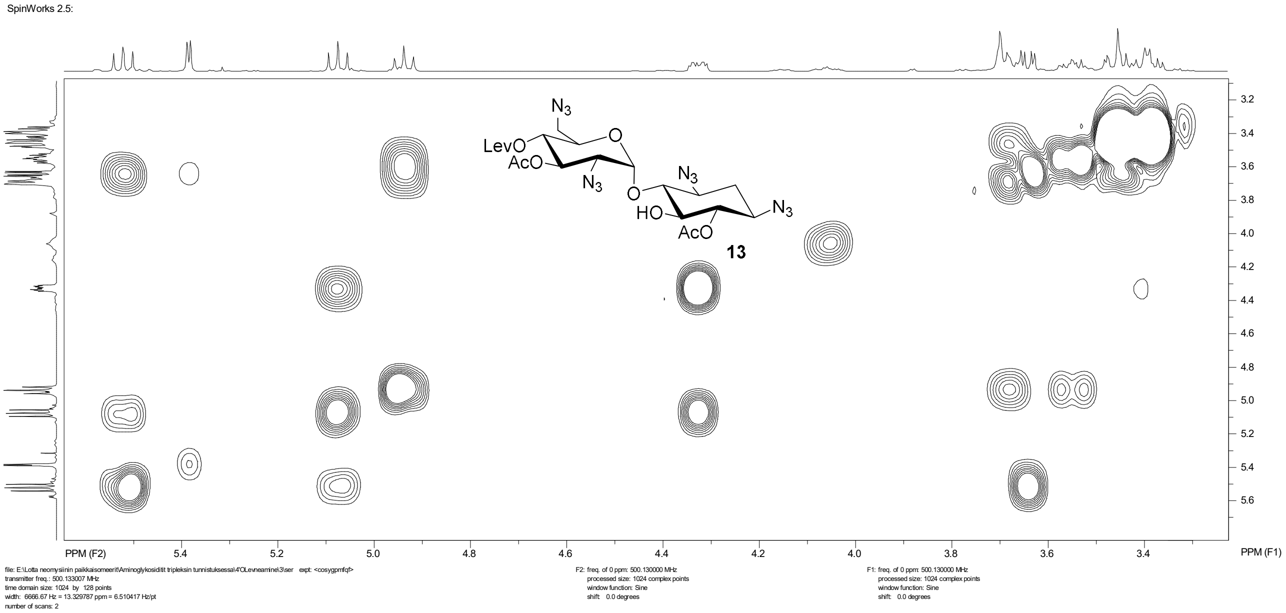

Figure S32. COSY spectrum of **13**.

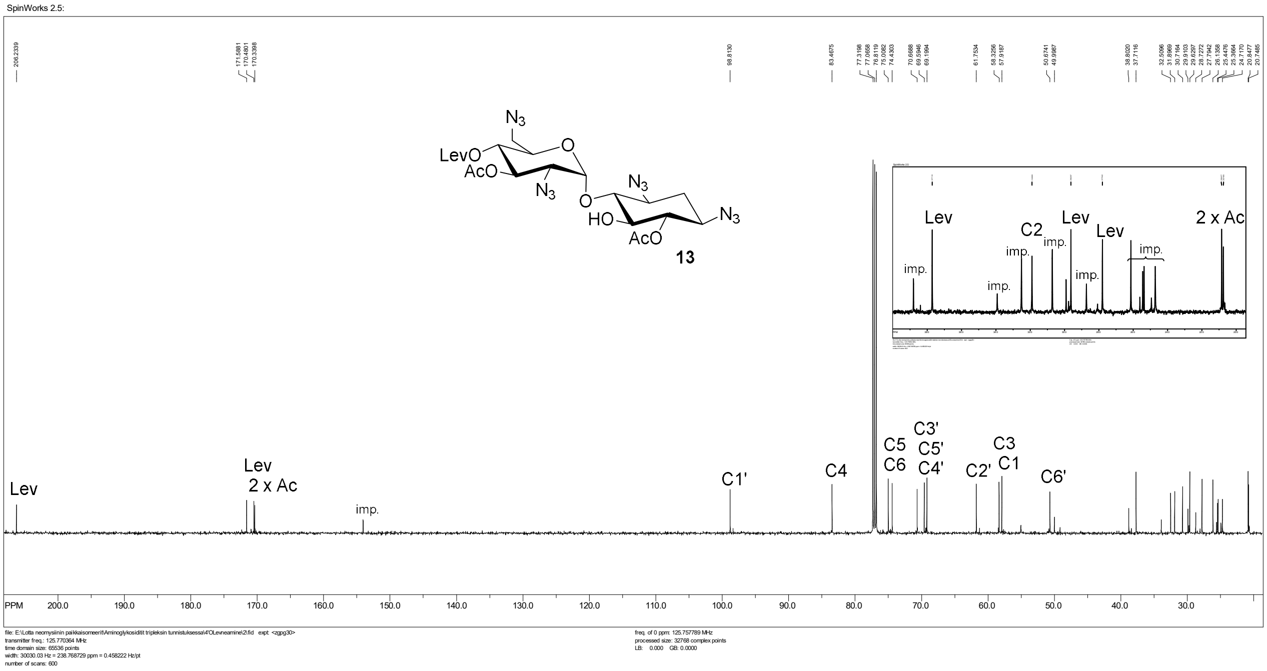

Figure S33. ^{13}C NMR (125 MHz, $CDCl_3$) spectrum of **13**.

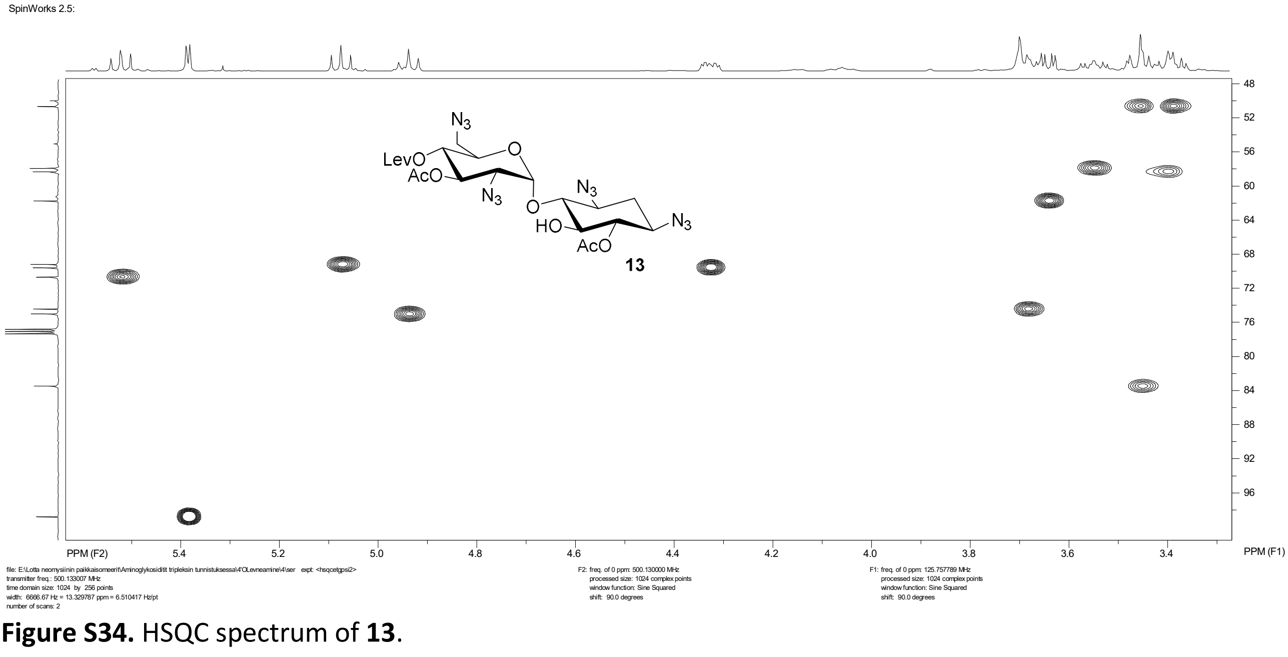

Figure S34. HSQC spectrum of **13**.

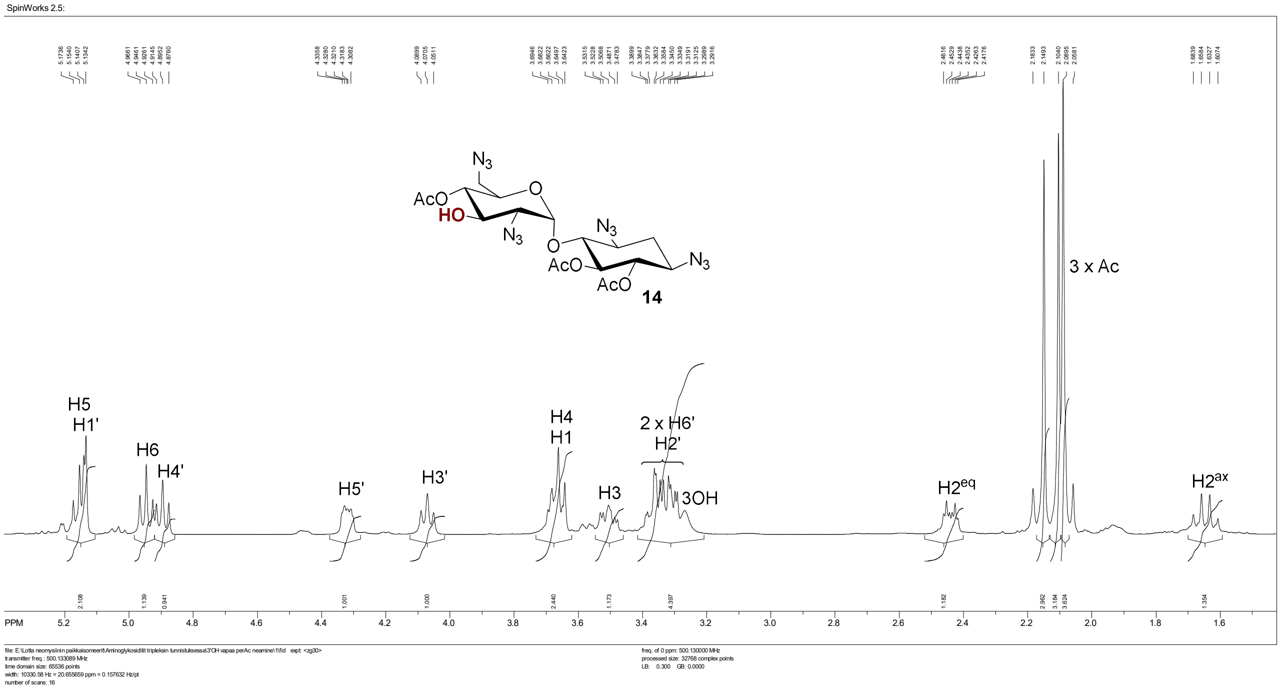

Figure S35. ^{1}H NMR (500 MHz, $CDCl_3$) spectrum of **14**.

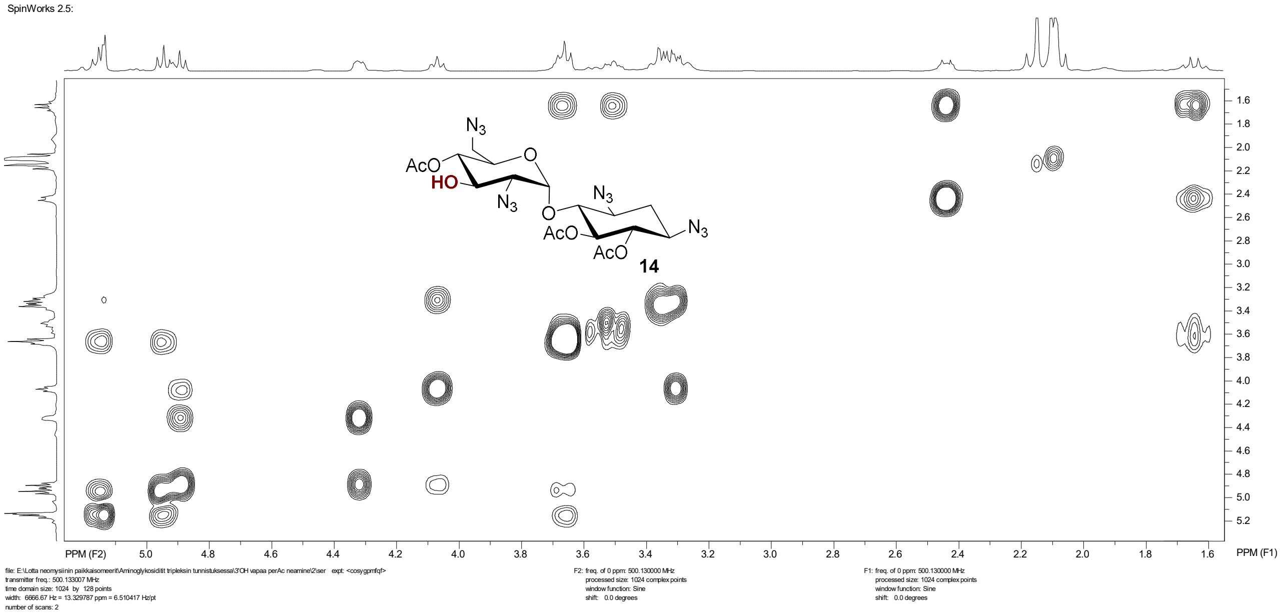

Figure S36. COSY spectrum of **14**.

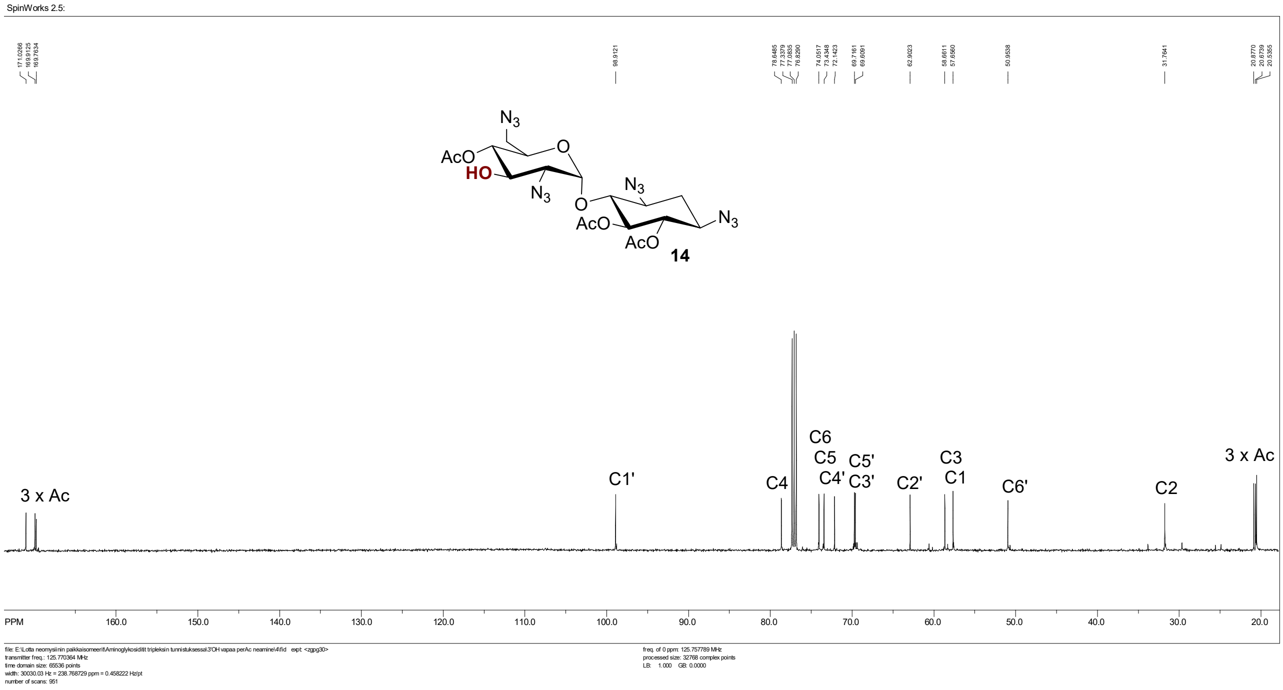

Figure S37. ^{13}C NMR (125 MHz, $CDCl_3$) spectrum of **14**.

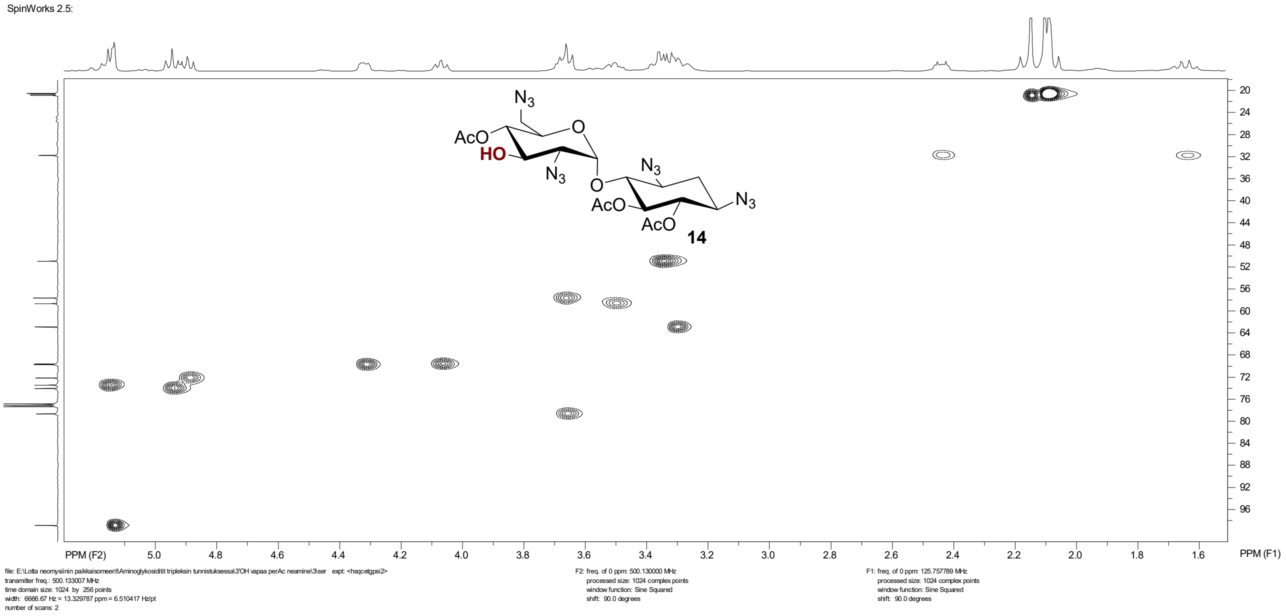

Figure S38. HSQC spectrum of **14**.

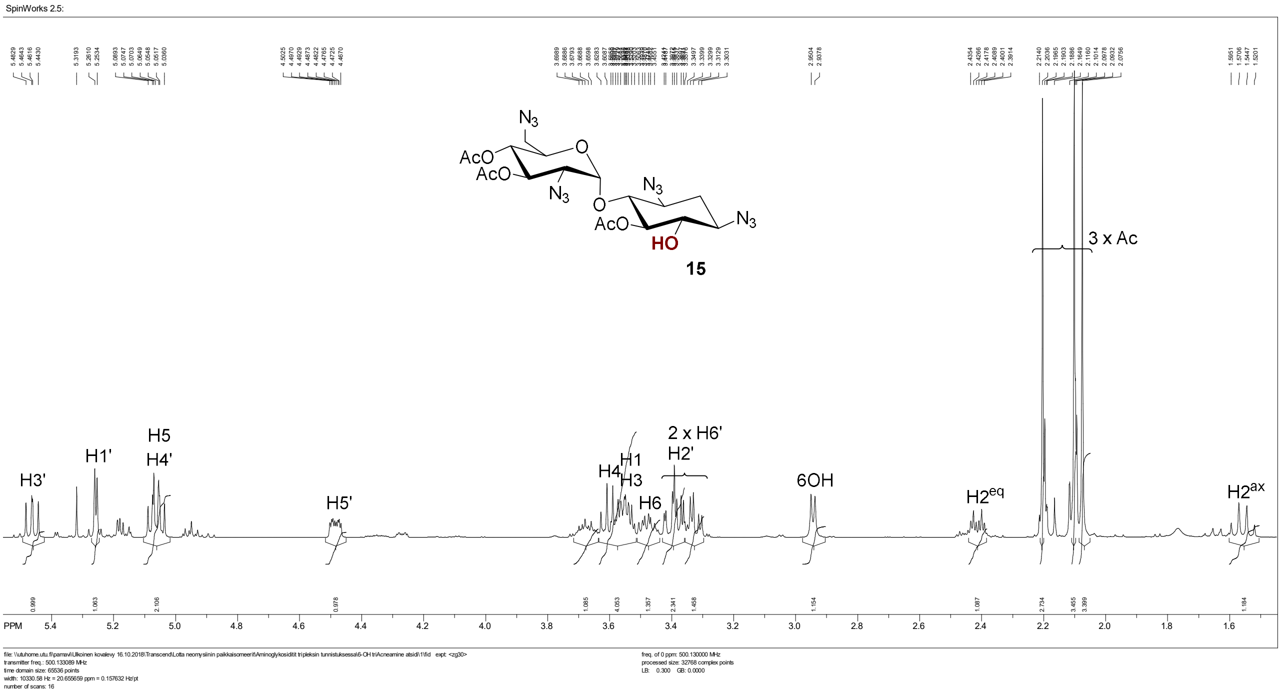

Figure S39. ^{1}H NMR (500 MHz, $CDCl_3$) spectrum of **15**.

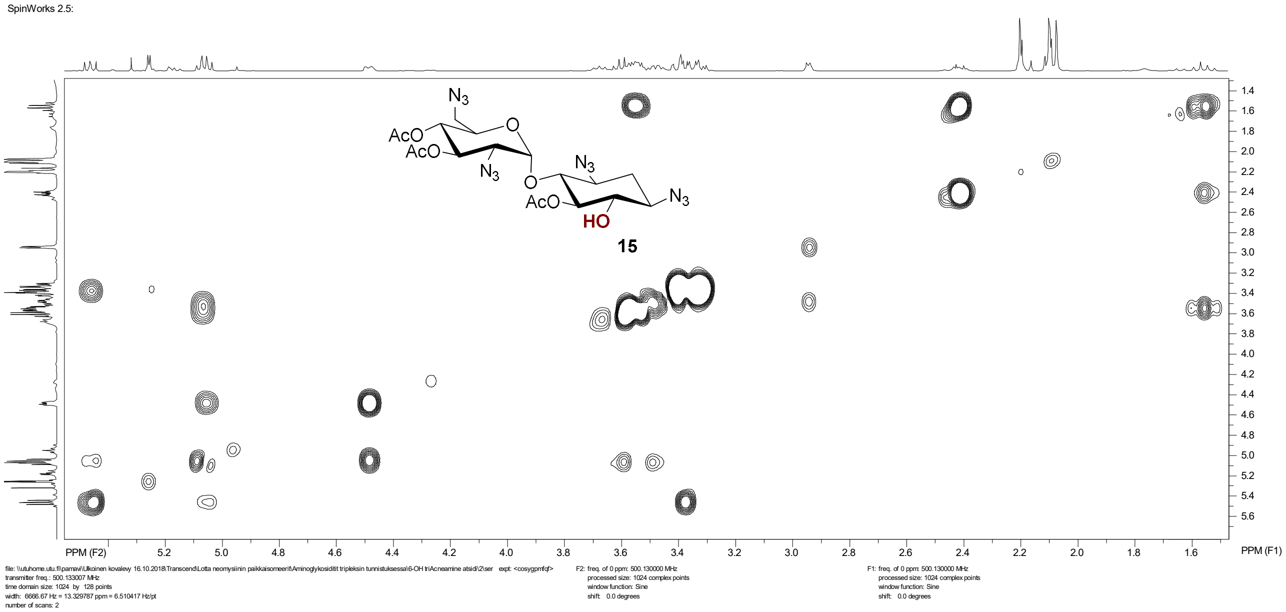

Figure S40. COSY spectrum of **15**.

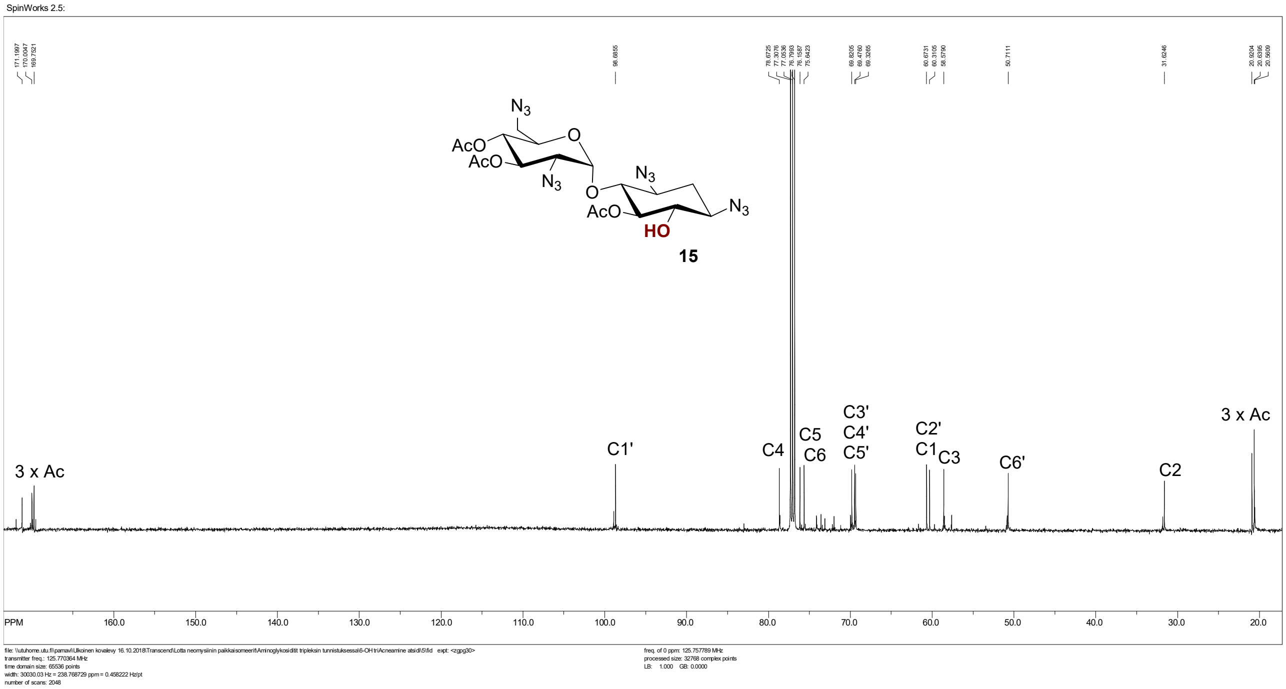

Figure S41. ^{13}C NMR (125 MHz, $CDCl_3$) spectrum of **15**.

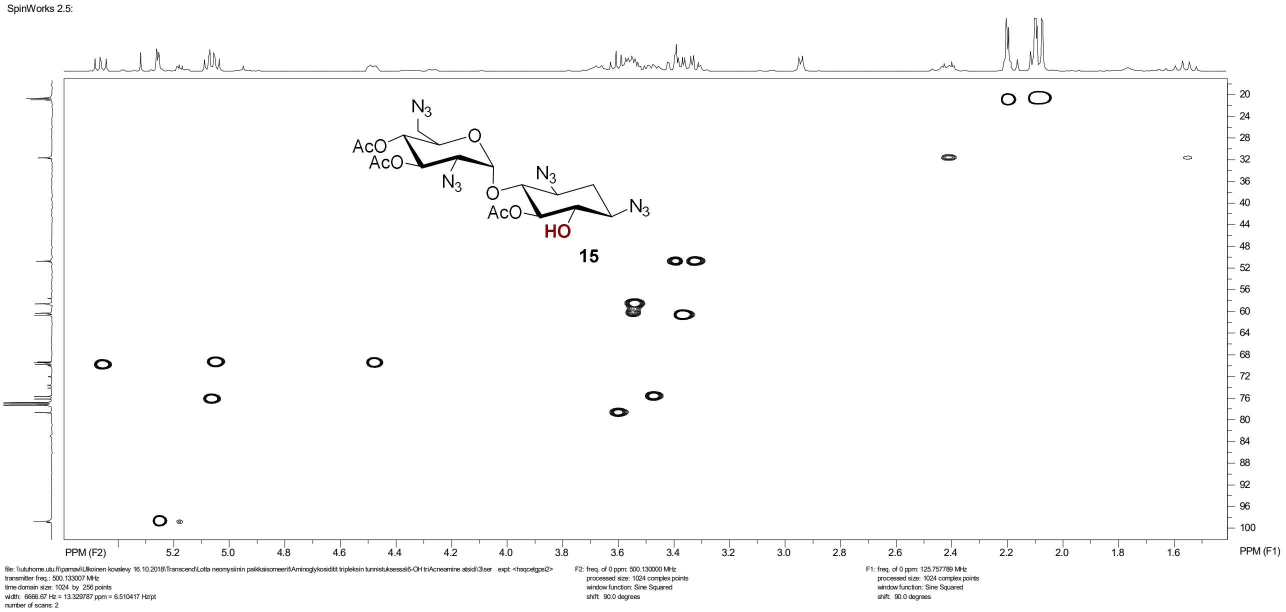

Figure S42. HSQC spectrum of **15**.

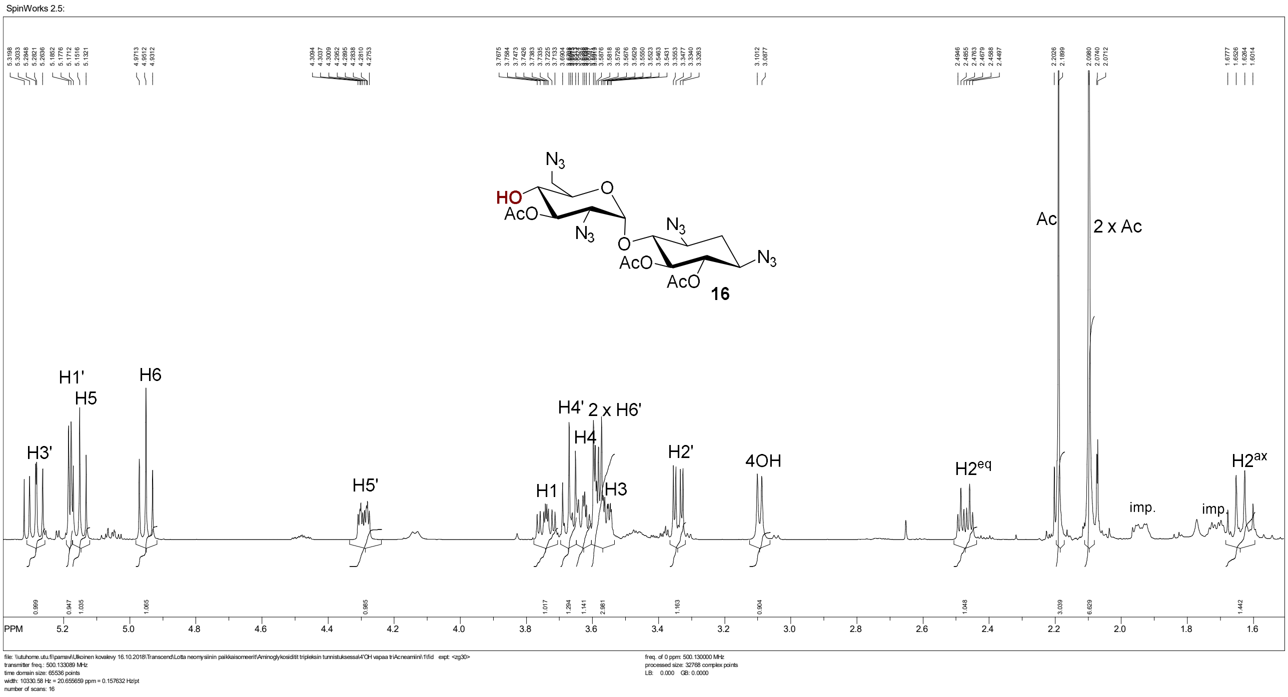

Figure S43. ^{1}H NMR (500 MHz, $CDCl_3$) spectrum of **16**.

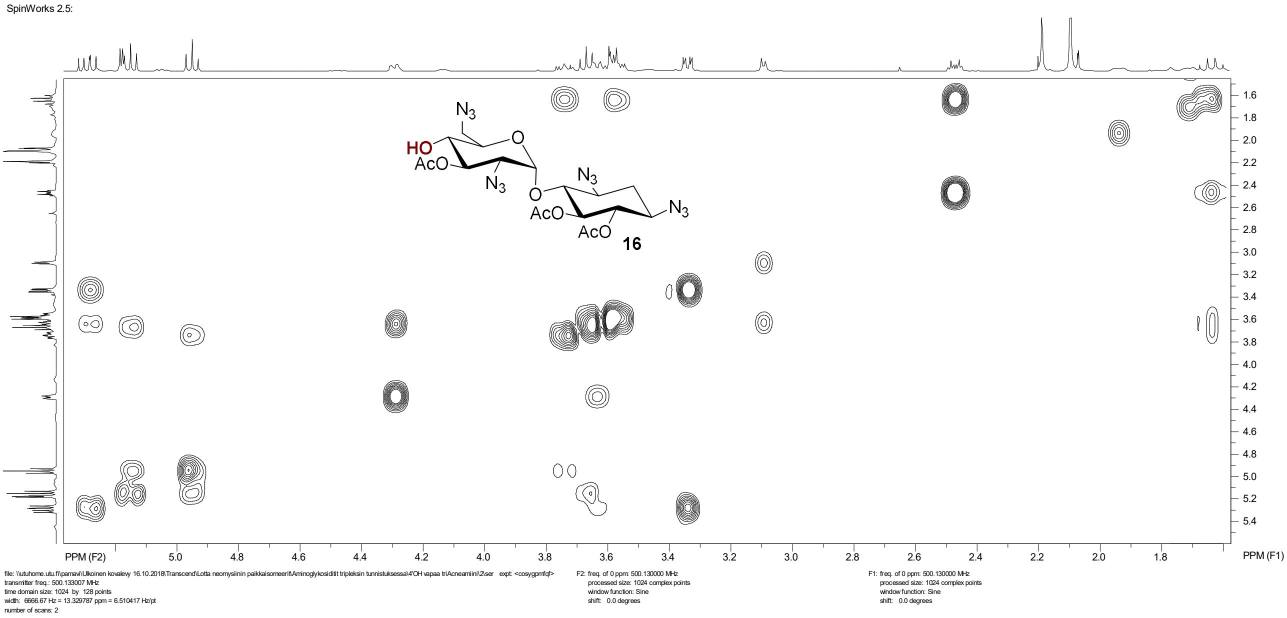

Figure S44. COSY spectrum of **16**.

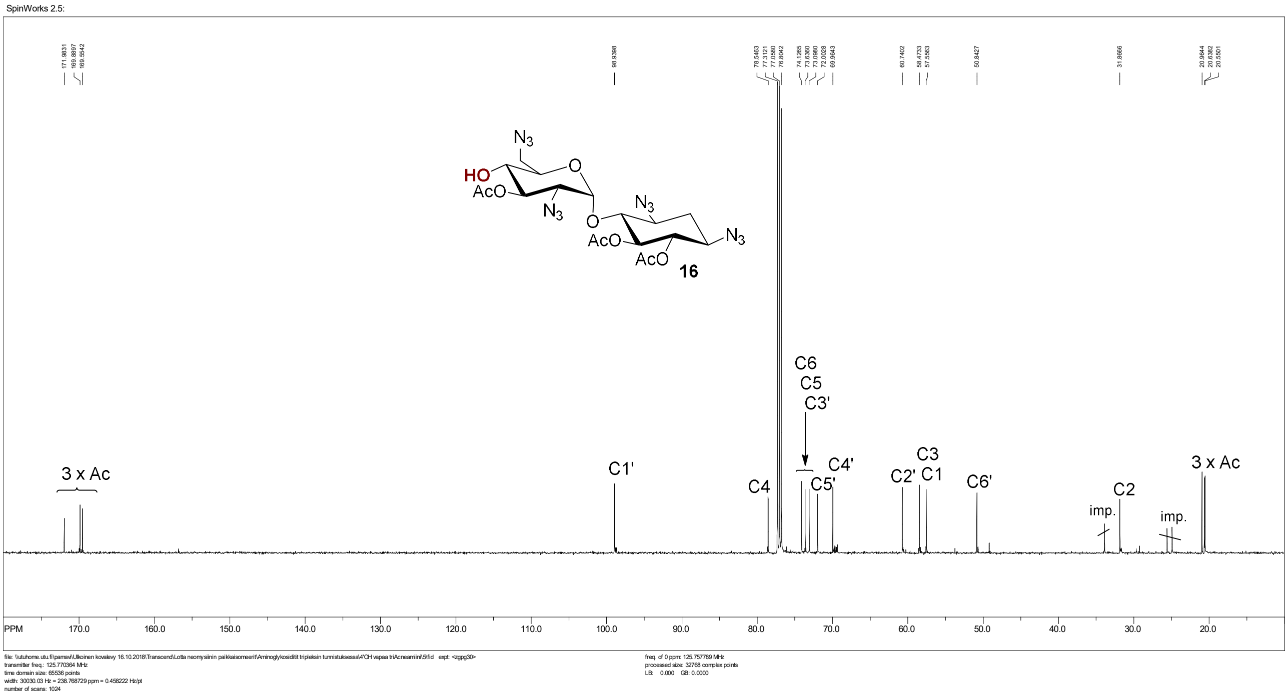

Figure S45. ^{13}C NMR (125 MHz, $CDCl_3$) spectrum of **16**.

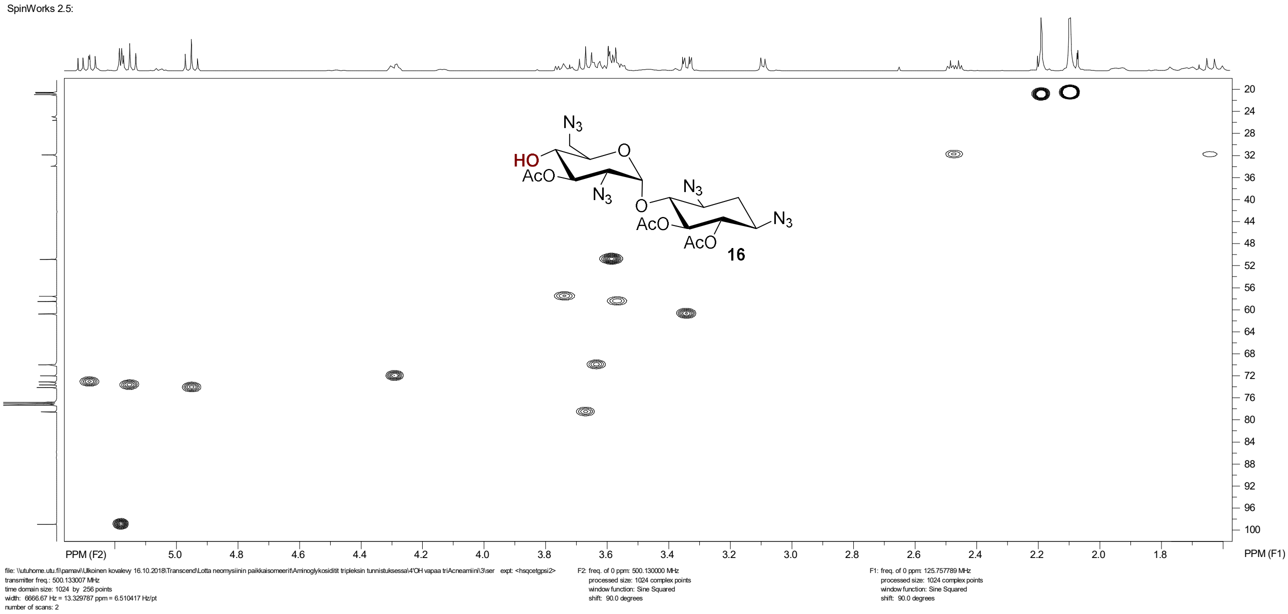

Figure S46. HSQC spectrum of **16**.

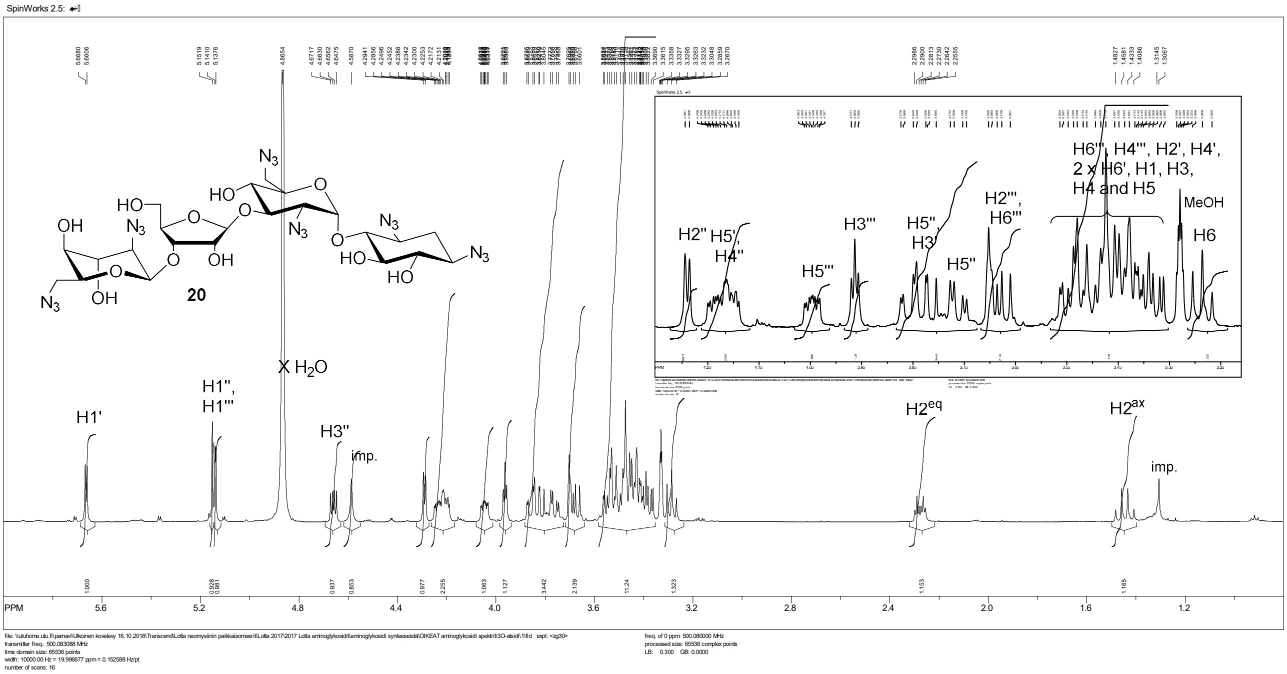

Figure S47. ^{1}H NMR (500 MHz, $CDCl_3$) spectrum of **20**.

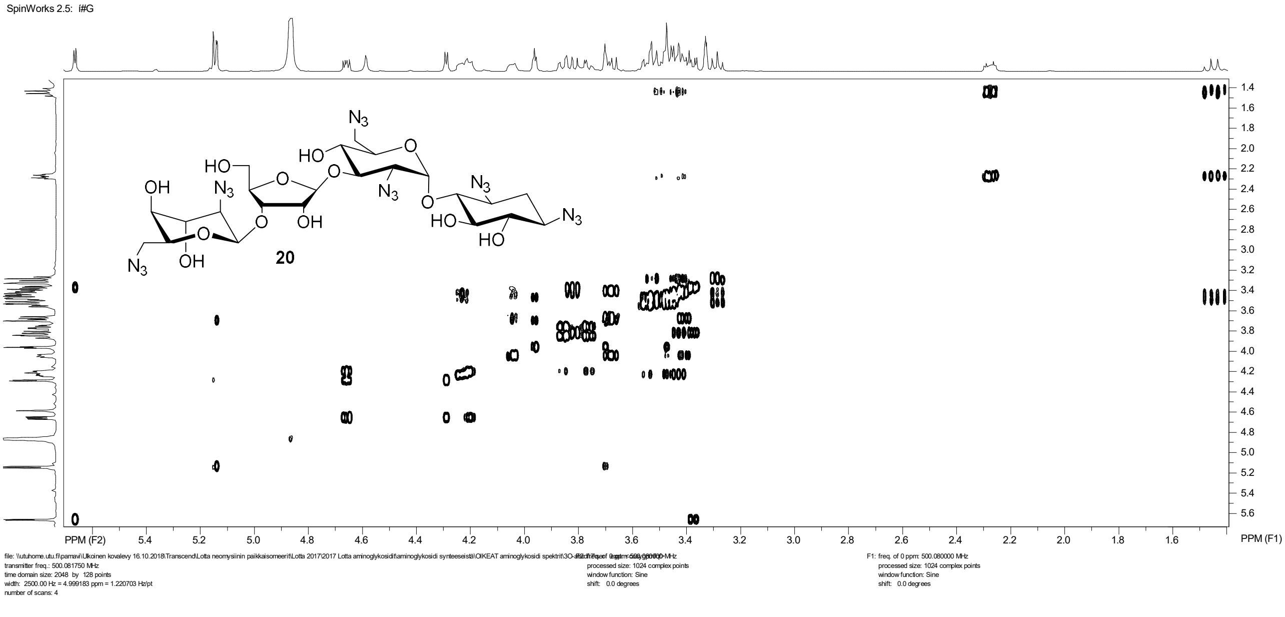

Figure S48. COSY spectrum of **20**.

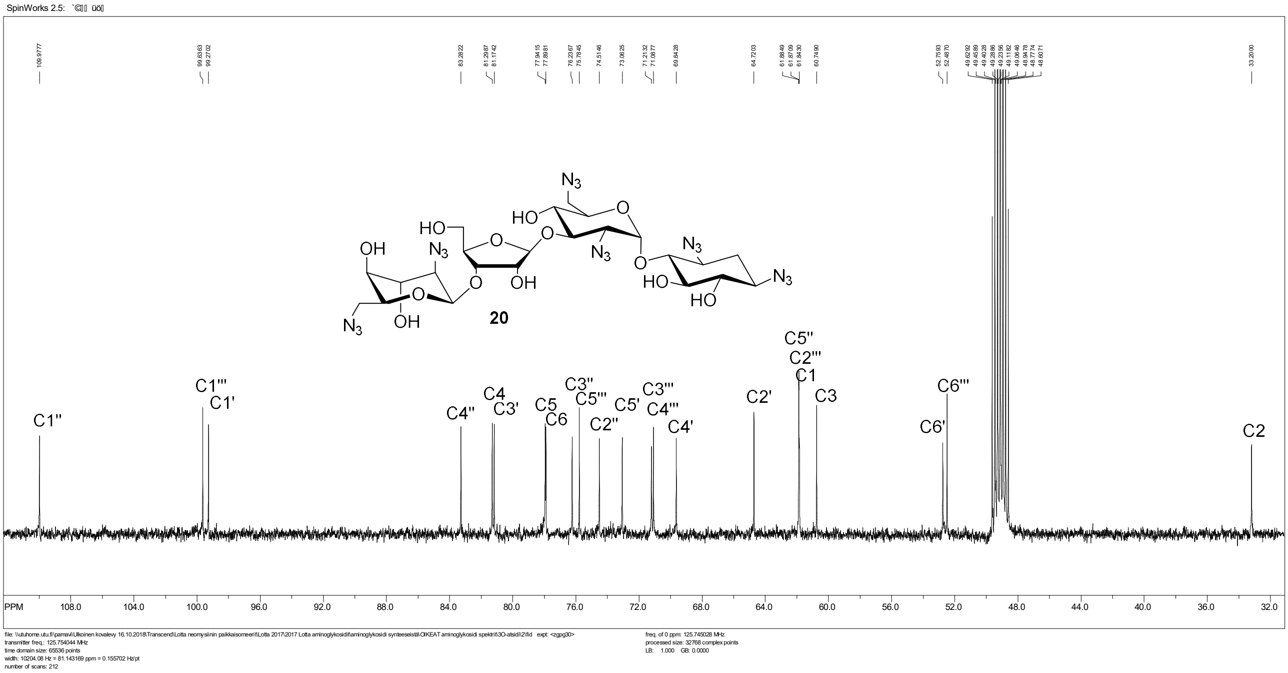

Figure S49. ^{13}C NMR (125 MHz, $CDCl_3$) spectrum of **20**.

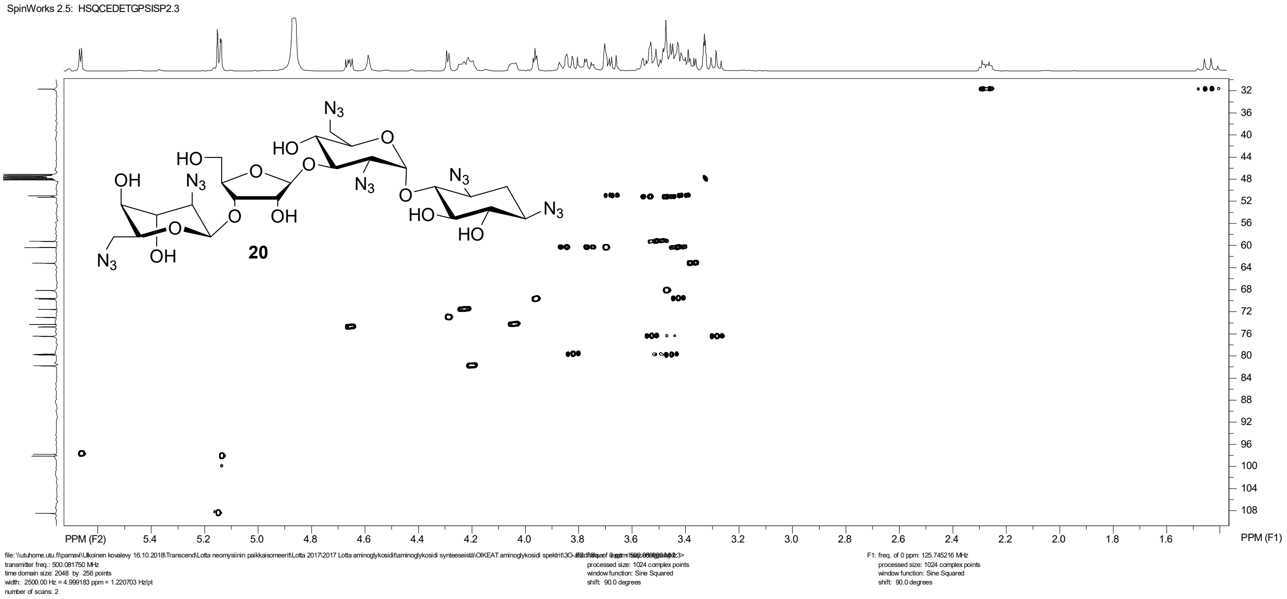

Figure S50. HSQC spectrum of **20**.

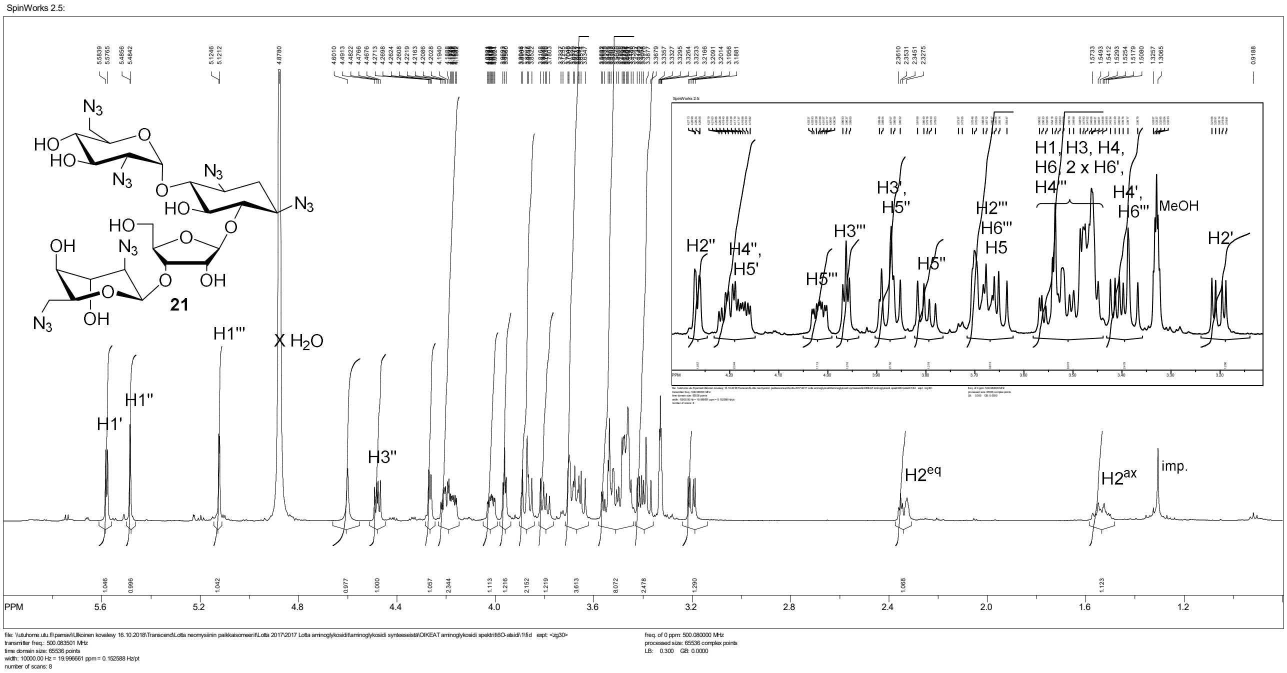

Figure S51. ^{1}H NMR (500 MHz, $CDCl_3$) spectrum of **21**.

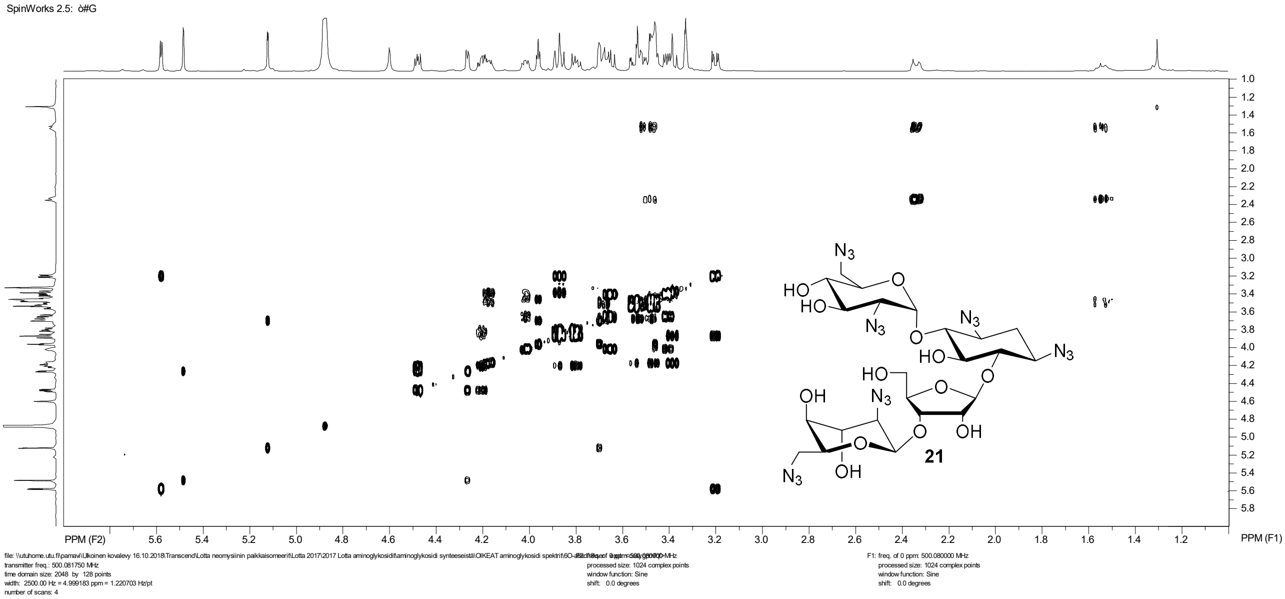

Figure S52. COSY spectrum of **21**.

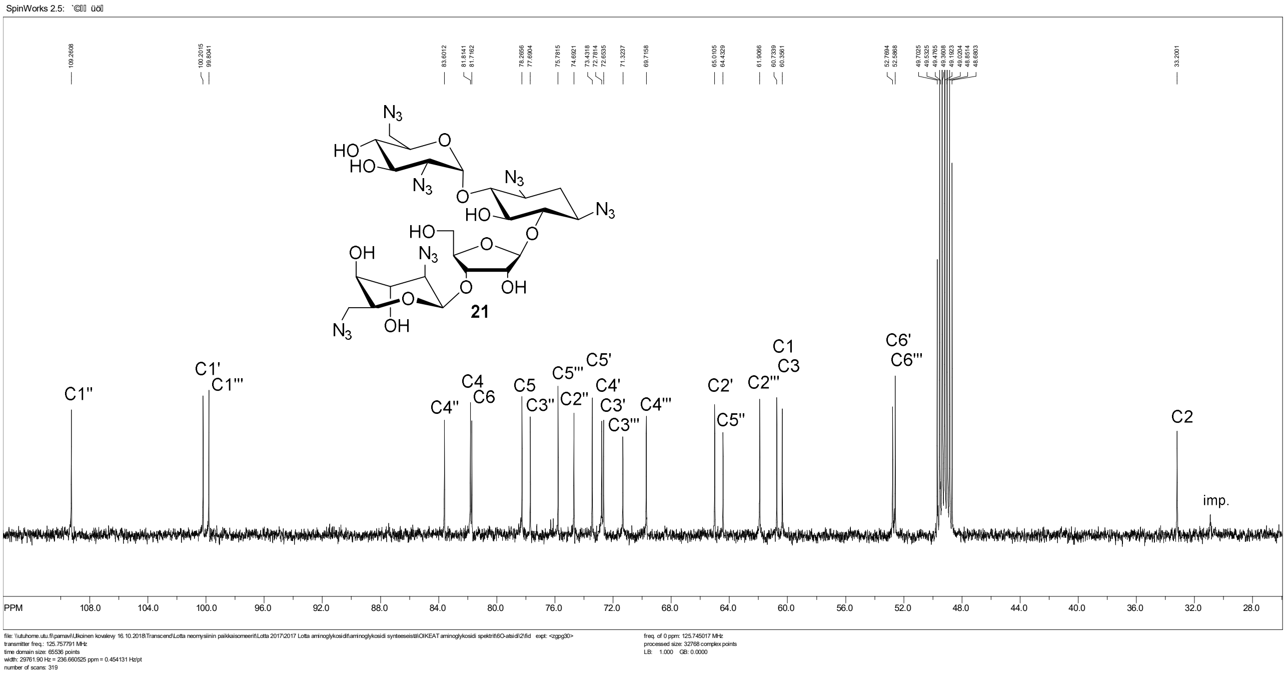

Figure S53. ^{13}C NMR (125 MHz, $CDCl_3$) spectrum of **21**.

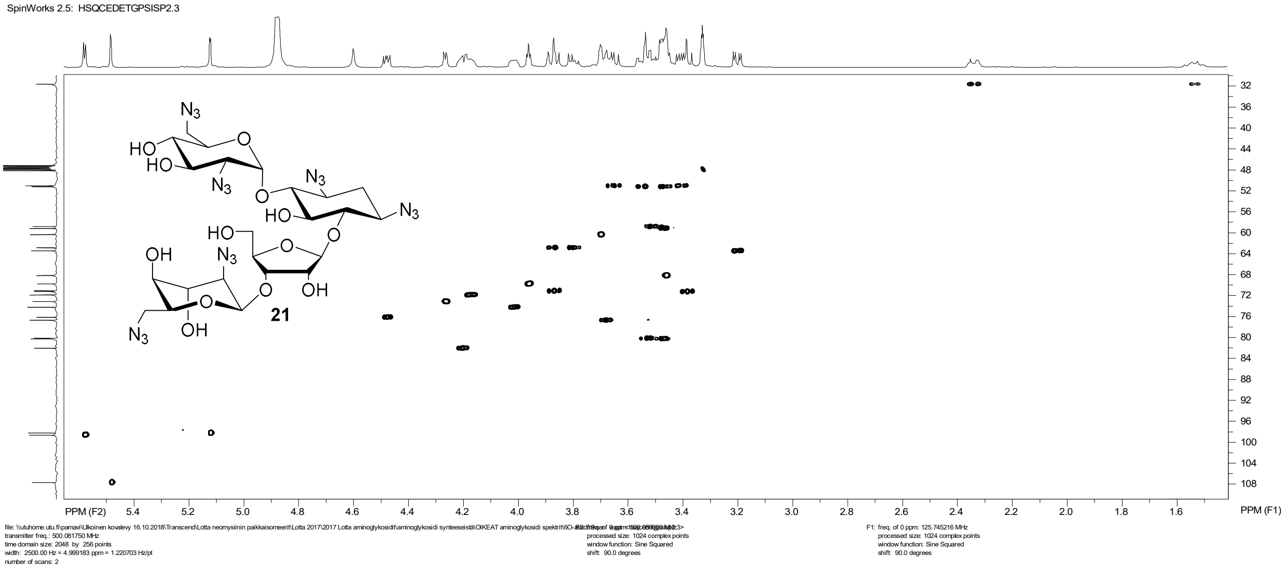

Figure S54. HSQC spectrum of **21**.

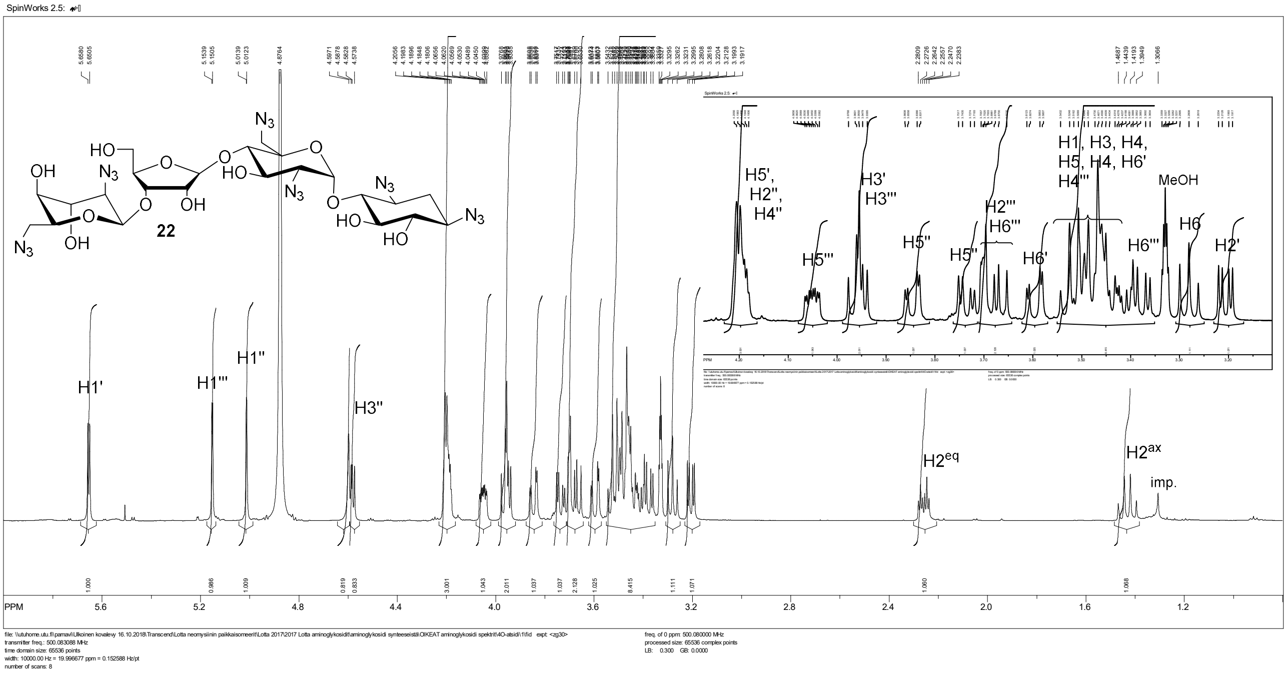

Figure S55. ^{1}H NMR (500 MHz, $CDCl_3$) spectrum of **22**.

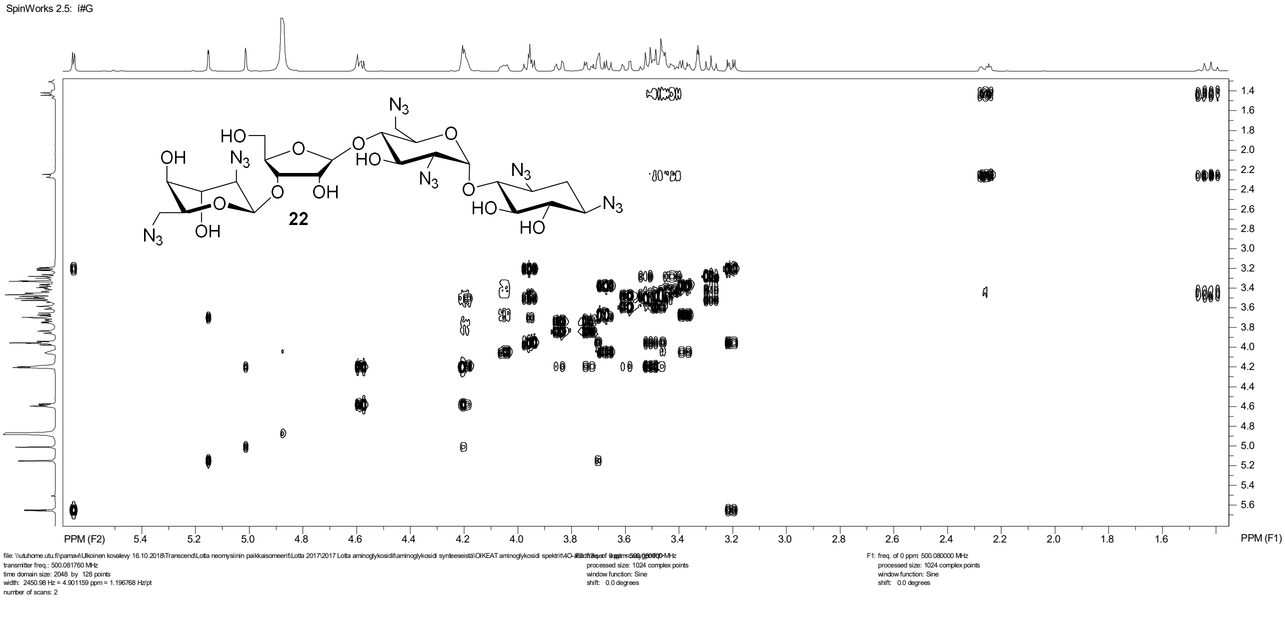

Figure S56. COSY spectrum of **22**.

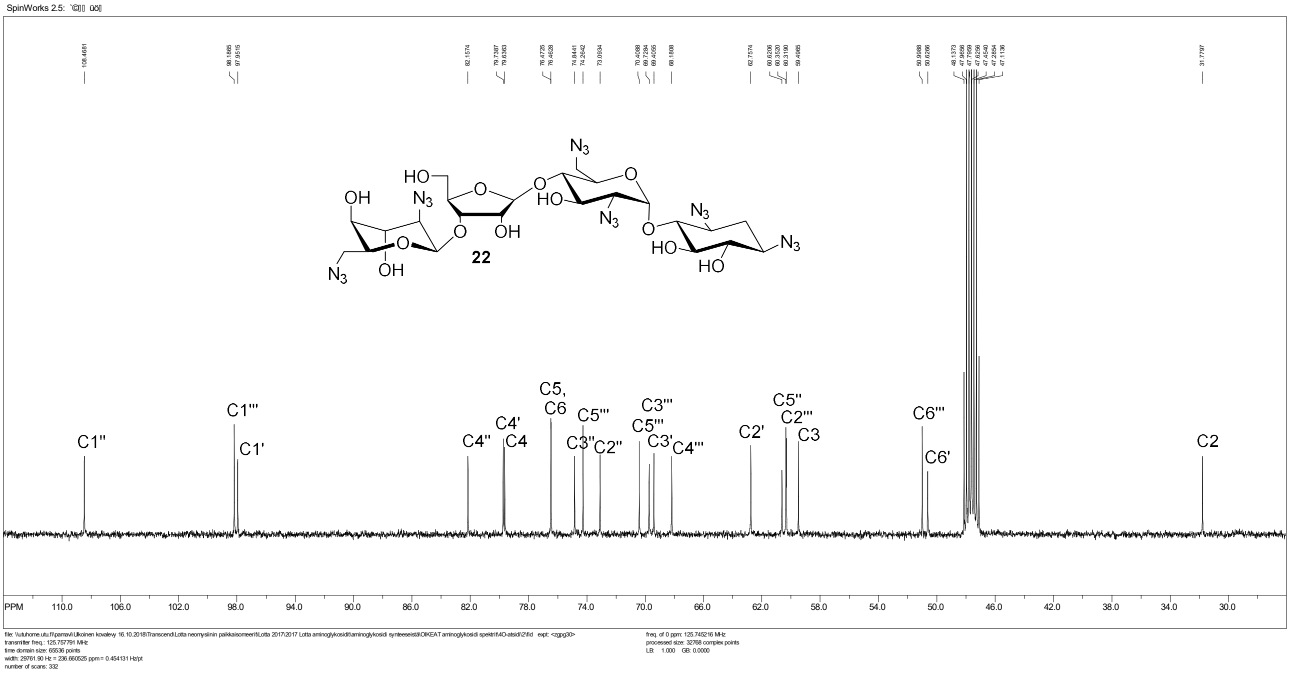

Figure S57. ^{13}C NMR (125 MHz, $CDCl_3$) spectrum of **22**.

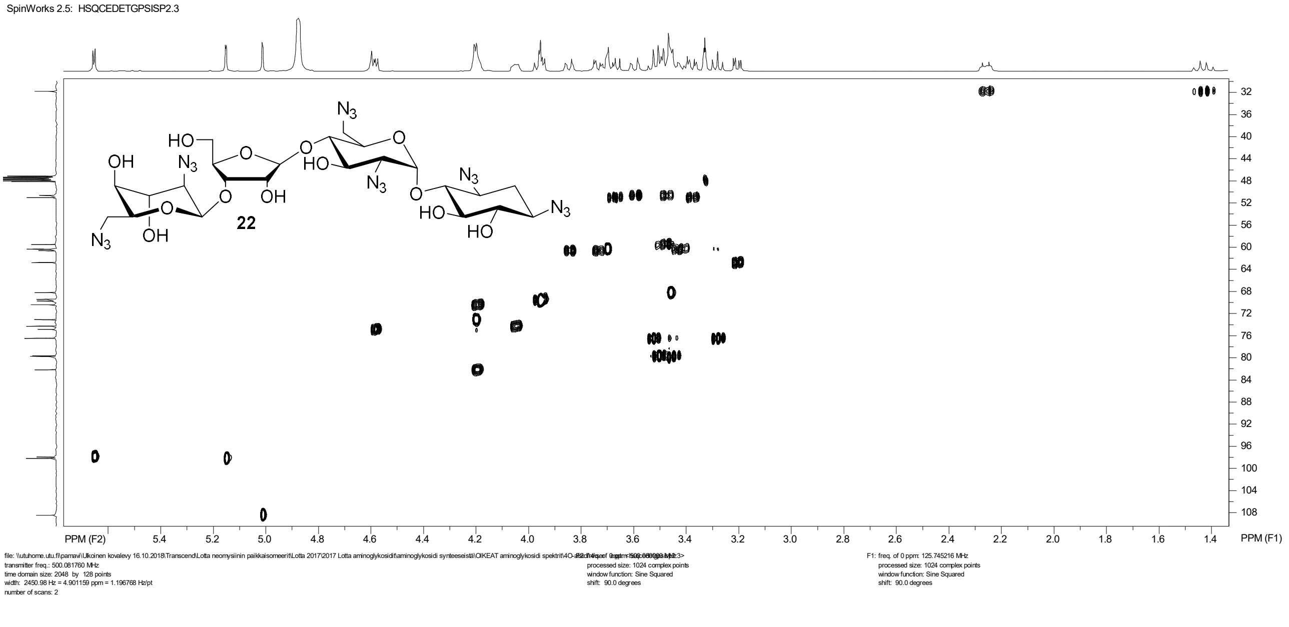

Figure S58. HSQC spectrum of **22**.

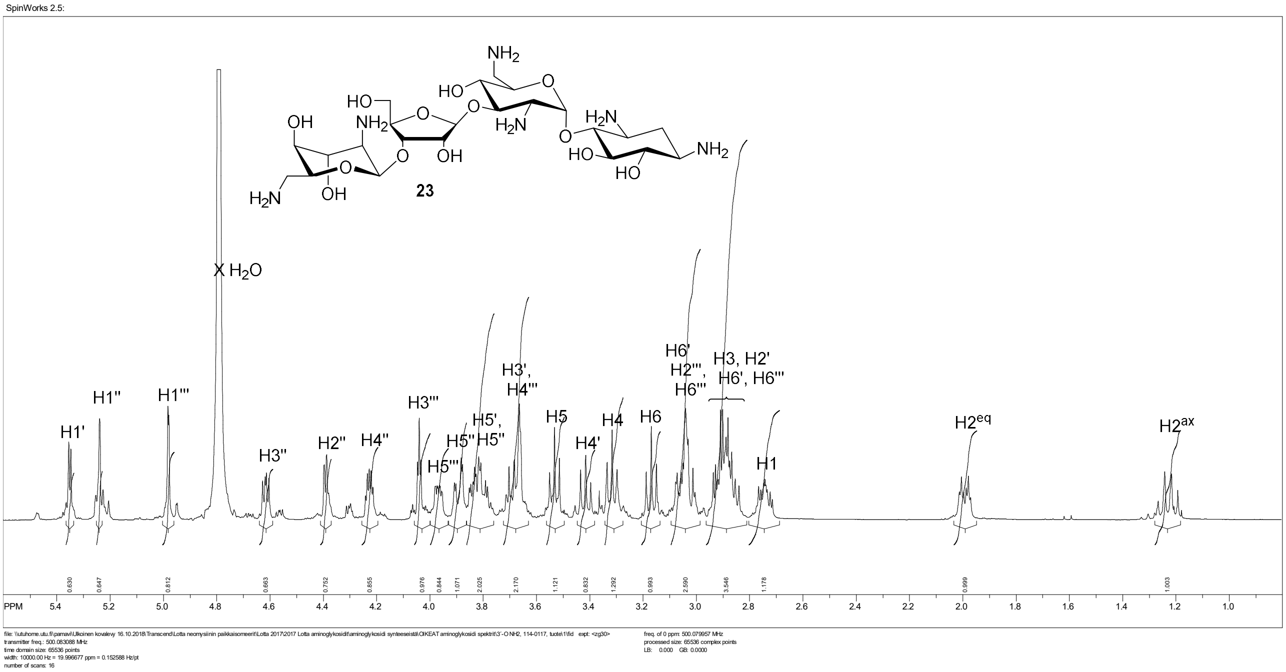

Figure S59. ^{1}H NMR (500 MHz, D_2O) spectrum of **23**.

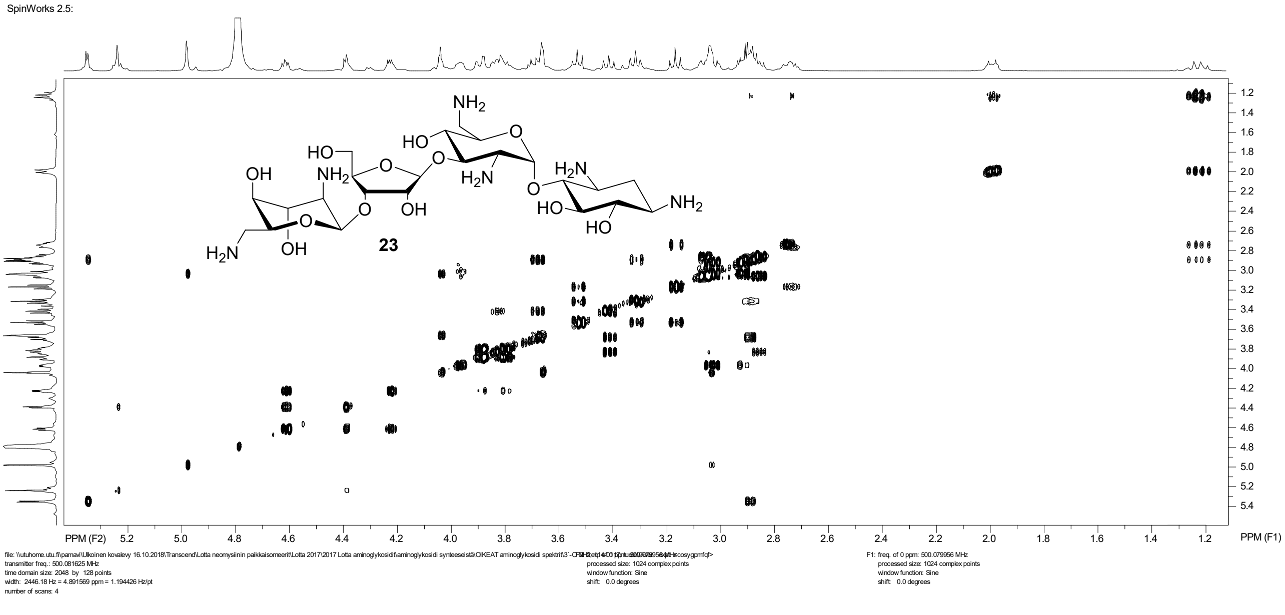

Figure S60. COSY spectrum of **23**.

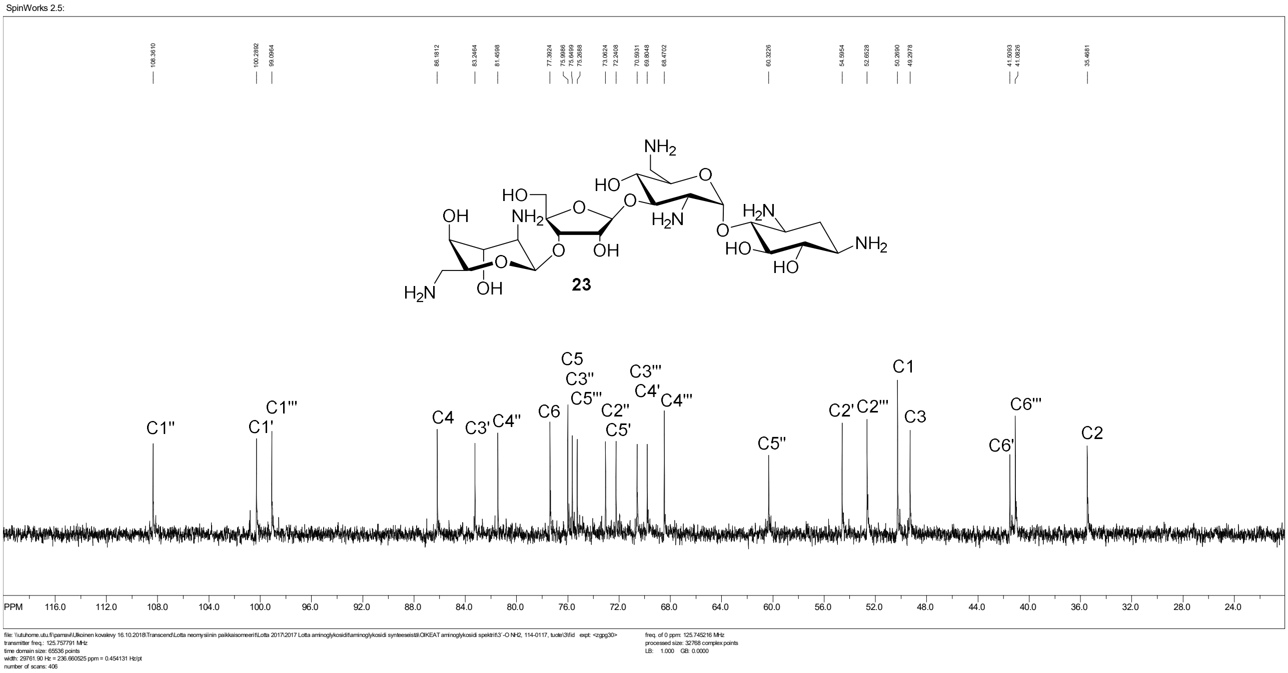

Figure S61. ^{13}C NMR (125 MHz, D_2O) spectrum of **23**.

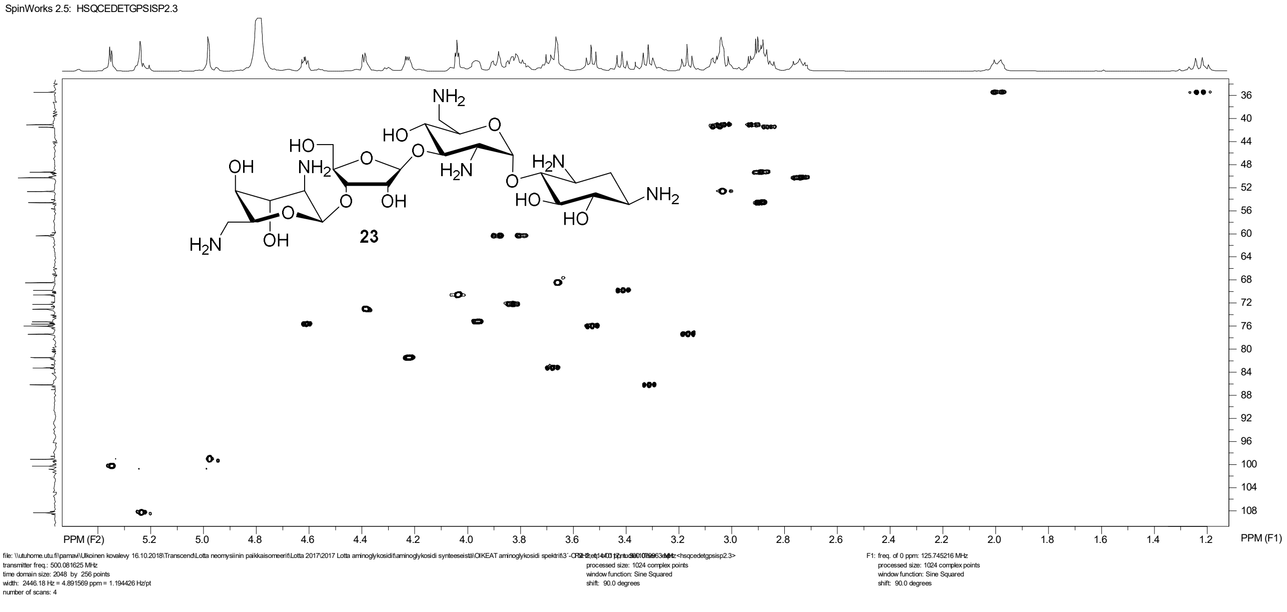

Figure S62. HSQC spectrum of **23**.

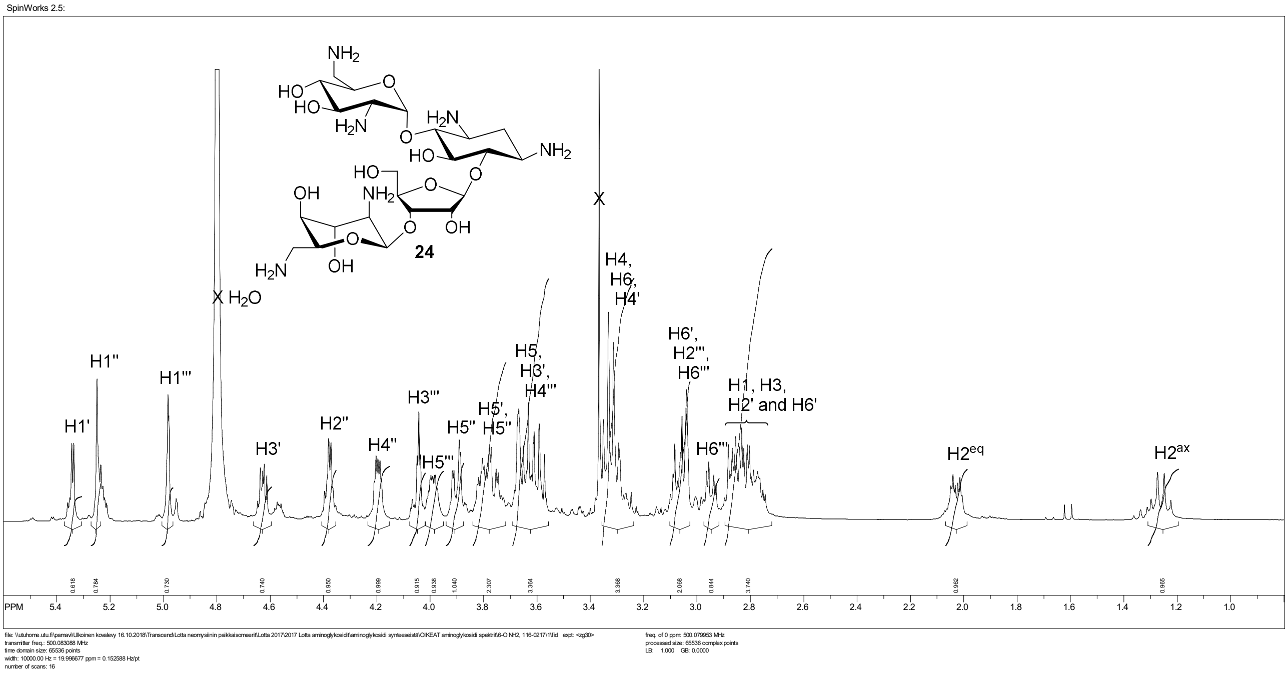

Figure S63. ^{1}H NMR (500 MHz, D_2O) spectrum of **24**.

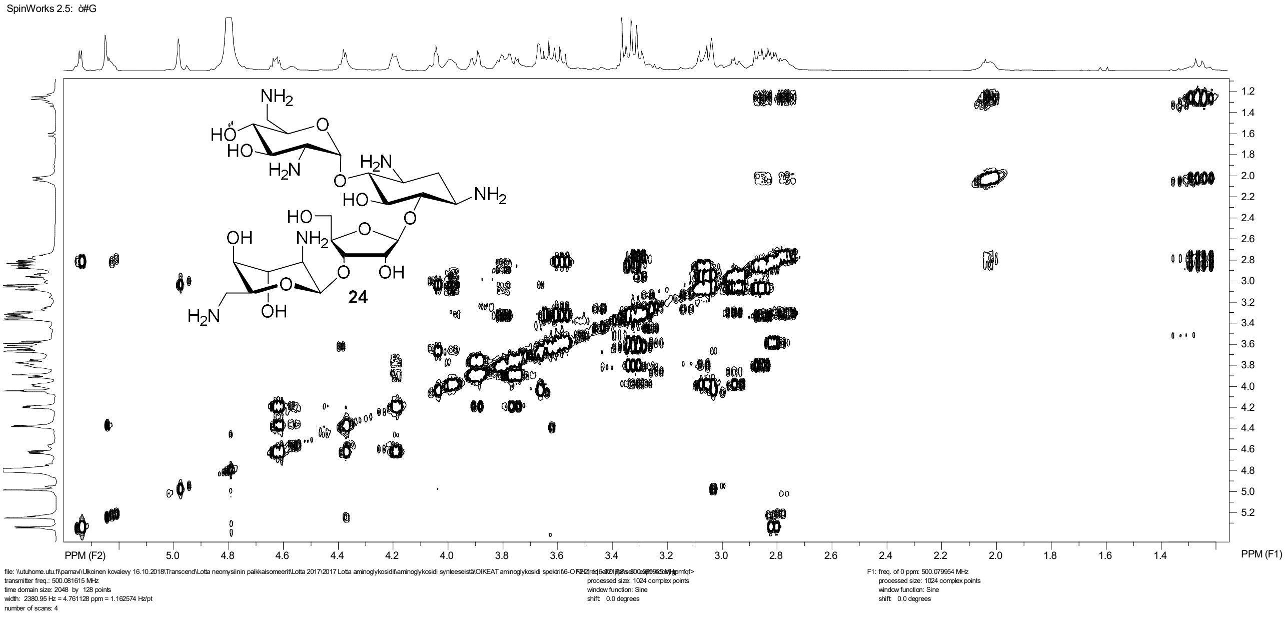

Figure S64. COSY spectrum of **24**.

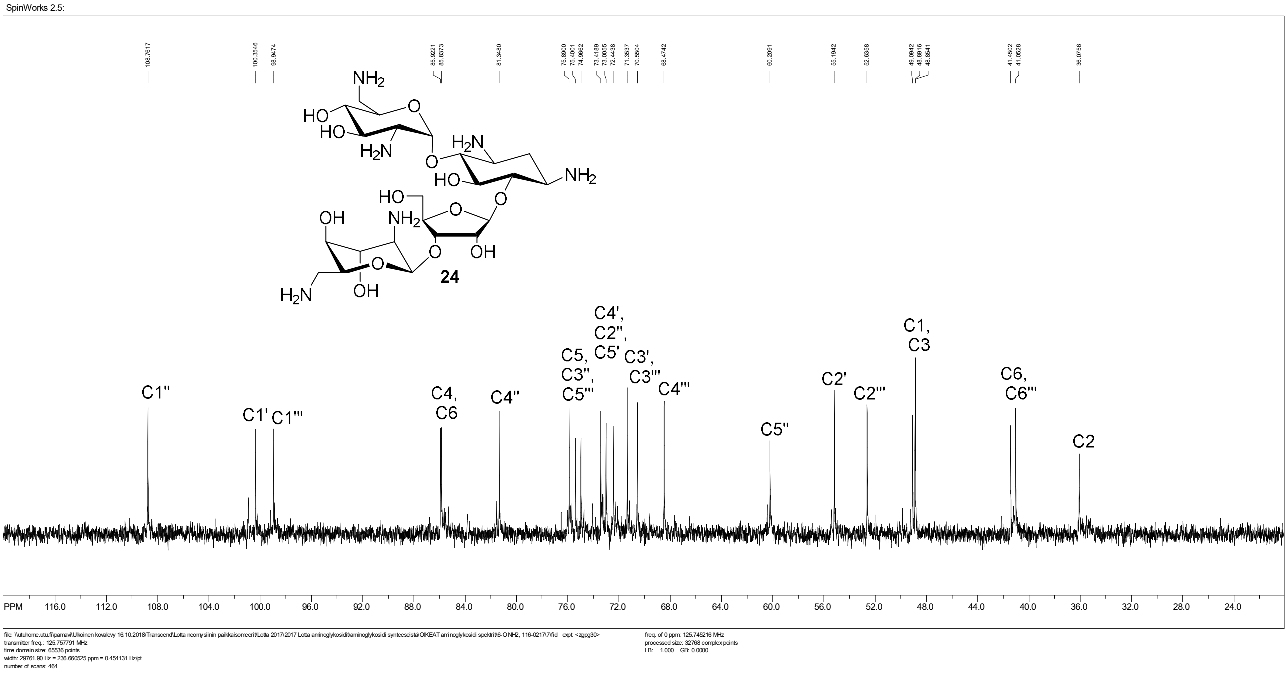

Figure S65. ^{13}C NMR (125 MHz, D_2O) spectrum of **24**.

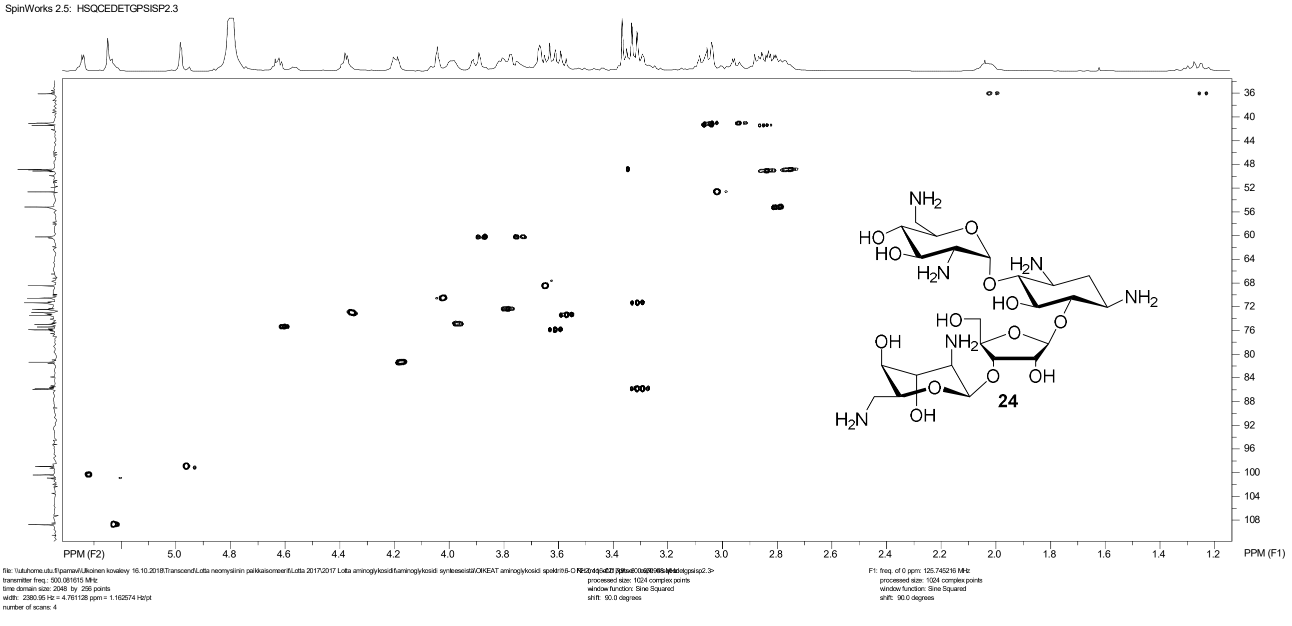

Figure S66. HSQC spectrum of **24**.

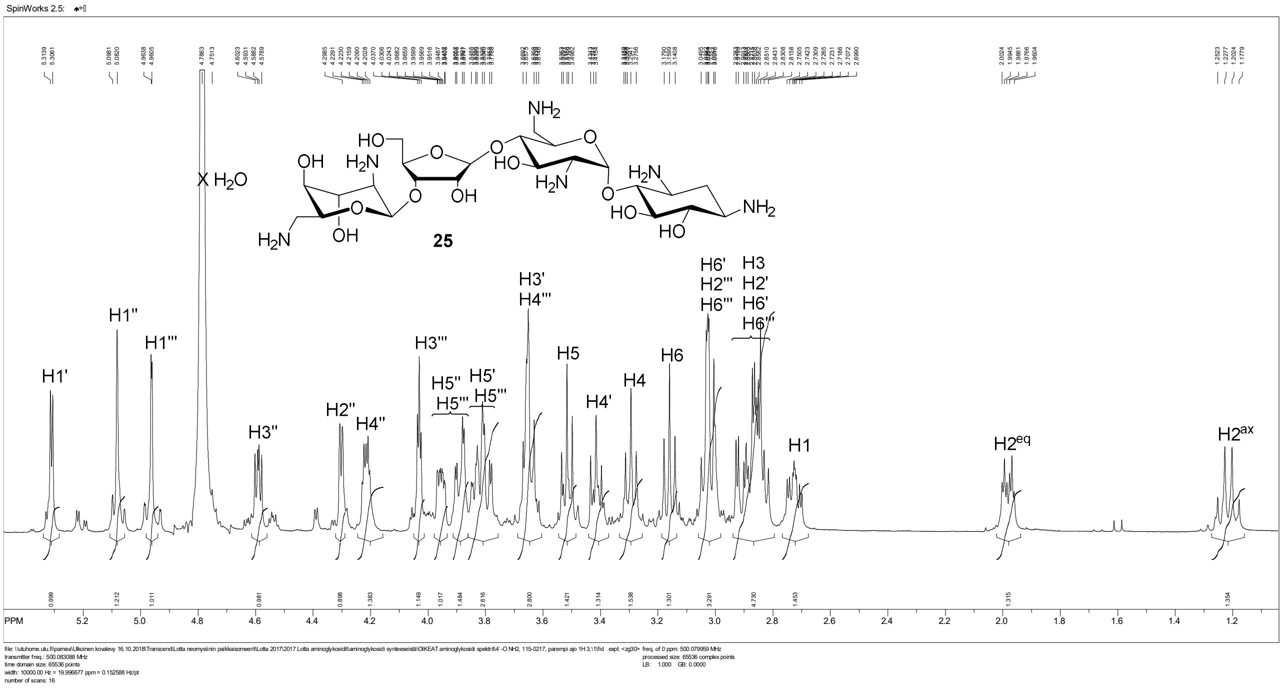

Figure S67. ^{1}H NMR (500 MHz, D_2O) spectrum of **25**.

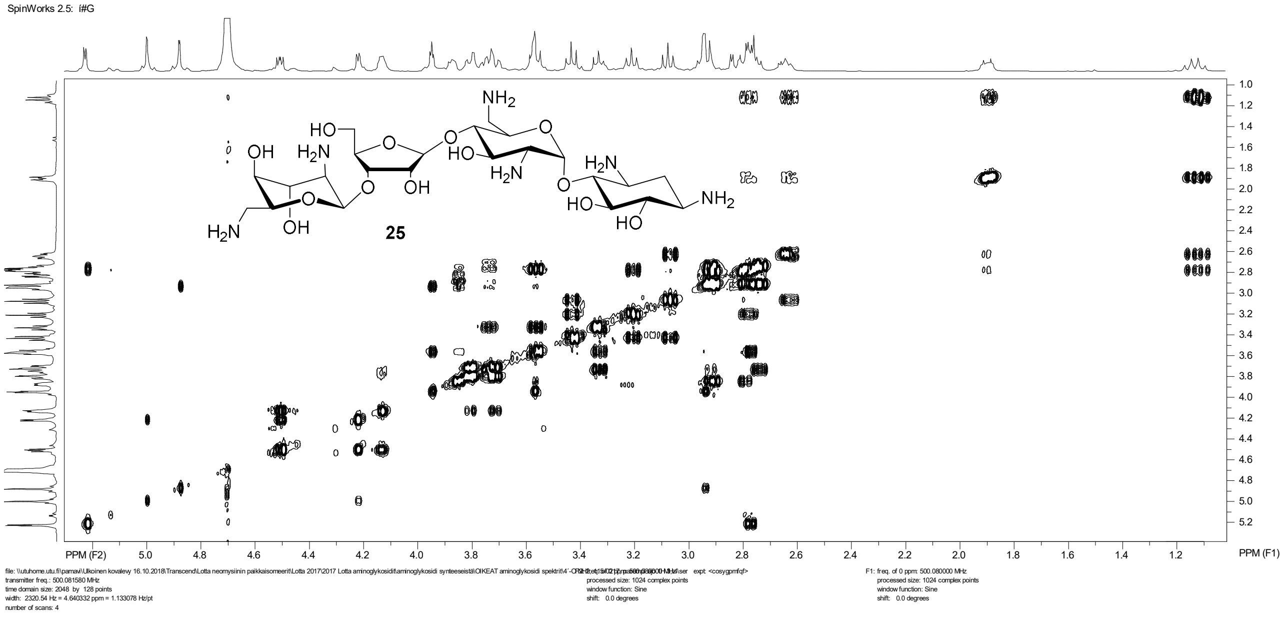

Figure S68. COSY pectrum of **25**.

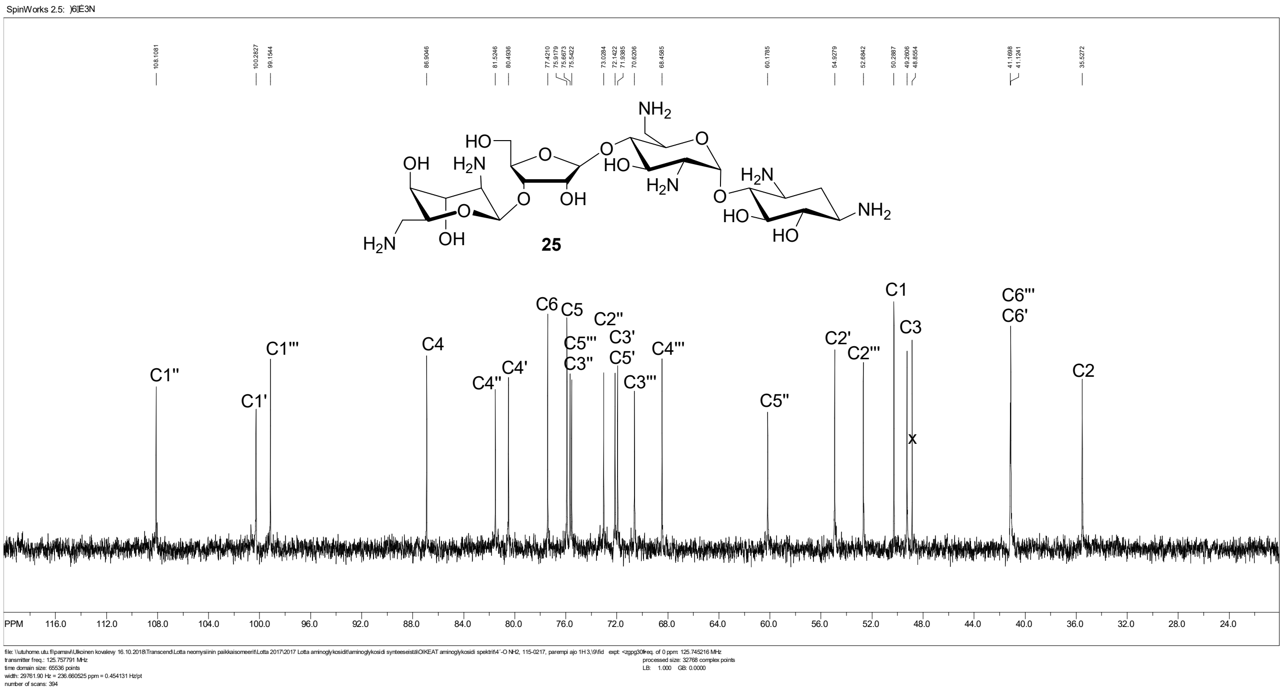

Figure S69. ^{13}C NMR (125 MHz, D_2O) spectrum of **25**.

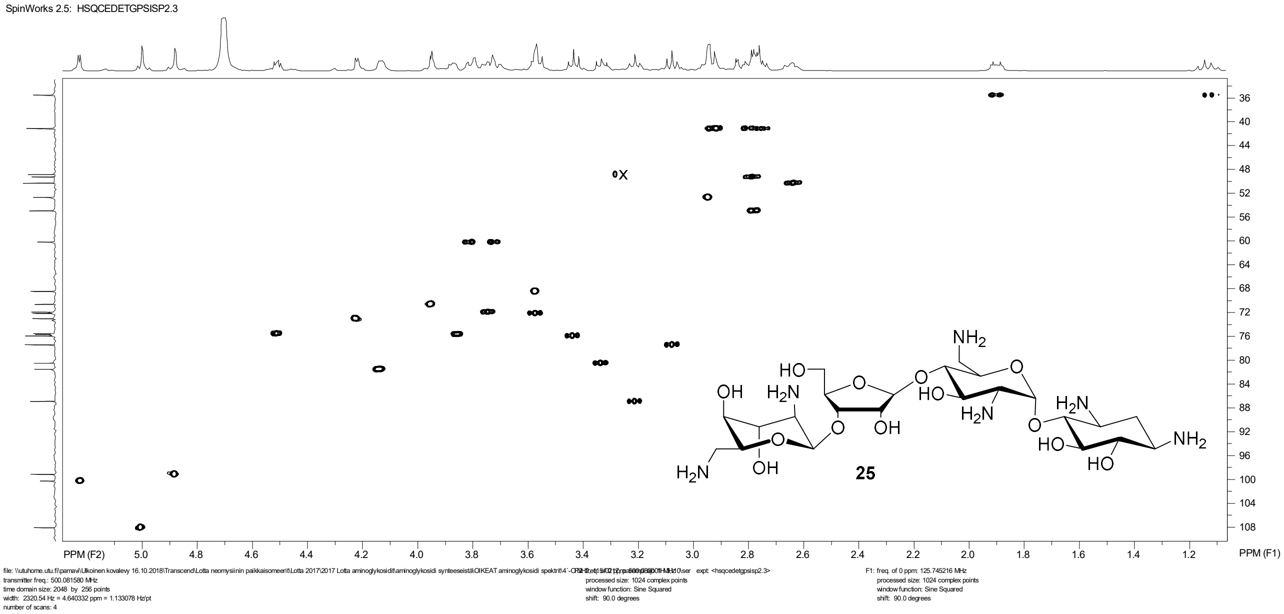

Figure S70. HSQC spectrum of **25**.
